# Safety of drug use in patients with a primary mitochondrial disease: An international Delphi‐based consensus

**DOI:** 10.1002/jimd.12196

**Published:** 2020-02-07

**Authors:** Maaike C. De Vries, David A. Brown, Mitchell E. Allen, Laurence Bindoff, Gráinne S. Gorman, Amel Karaa, Nandaki Keshavan, Costanza Lamperti, Robert McFarland, Yi Shiau Ng, Mar O'Callaghan, Robert D. S. Pitceathly, Shamima Rahman, Frans G. M. Russel, Kristin N. Varhaug, Tom J. J. Schirris, Michelangelo Mancuso

**Affiliations:** ^1^ Radboudumc Amalia Children's Hospital Radboud Center for Mitochondrial Medicine Nijmegen The Netherlands; ^2^ Department of Human Nutrition, Foods, and Exercise and the Virginia Tech Center for Drug Discovery Virginia Tech Blacksburg Virginia; ^3^ Department of Clinical Medicine University of Bergen Bergen Norway; ^4^ Department of Neurology Haukeland University Hospital Bergen Norway; ^5^ Wellcome Centre for Mitochondrial Research, Institute of Neuroscience Newcastle University Newcastle upon Tyne UK; ^6^ The Newcastle upon Tyne Hospitals NHS Foundation Trust Newcastle upon Tyne UK; ^7^ Genetics Unit, Massachusetts General Hospital Harvard Medical School Boston Massachusetts; ^8^ Mitochondrial Research Group UCL Great Ormond Street Institute of Child Health London UK; ^9^ Metabolic Unit Great Ormond Street Hospital NHS Foundation Trust London UK; ^10^ Unit of Medical Genetics and Neurogenetics Fondazione IRCCS Istituto Neurologico Carlo Besta Milan Italy; ^11^ Department of Neurology, Metabolic Unit Hospital Sant Joan de Déu Barcelona Spain; ^12^ CIBERER Instituto de Salud Carlos III Barcelona Spain; ^13^ Department of Neuromuscular Diseases UCL Queen Square Institute of Neurology and The National Hospital for Neurology and Neurosurgery London UK; ^14^ Department of Pharmacology and Toxicology Radboud Institute for Molecular Life Sciences, Radboud Center for Mitochondrial Medicine, Radboudumc Nijmegen The Netherlands; ^15^ Department of Clinical and Experimental Medicine, Neurological Institute University of Pisa Pisa Italy

**Keywords:** drugs, in vitro studies, in vivo studies, mitochondrial diseases, mitochondrial toxicity, safety

## Abstract

Clinical guidance is often sought when prescribing drugs for patients with primary mitochondrial disease. Theoretical considerations concerning drug safety in patients with mitochondrial disease may lead to unnecessary withholding of a drug in a situation of clinical need. The aim of this study was to develop consensus on safe medication use in patients with a primary mitochondrial disease. A panel of 16 experts in mitochondrial medicine, pharmacology, and basic science from six different countries was established. A modified Delphi technique was used to allow the panellists to consider draft recommendations anonymously in two Delphi rounds with predetermined levels of agreement. This process was supported by a review of the available literature and a consensus conference that included the panellists and representatives of patient advocacy groups. A high level of consensus was reached regarding the safety of all 46 reviewed drugs, with the knowledge that the risk of adverse events is influenced both by individual patient risk factors and choice of drug or drug class. This paper details the consensus guidelines of an expert panel and provides an important update of previously established guidelines in safe medication use in patients with primary mitochondrial disease. Specific drugs, drug groups, and clinical or genetic conditions are described separately as they require special attention. It is important to emphasise that consensus‐based information is useful to provide guidance, but that decisions related to drug prescribing should always be tailored to the specific needs and risks of each individual patient. We aim to present what is current knowledge and plan to update this regularly both to include new drugs and to review those currently included.

## 1 INTRODUCTION

Mitochondrial diseases are a group of inherited metabolic disorders that can present at any age and often exhibit multisystem involvement and high morbidity and mortality.^1^ The prevalence is conservatively estimated at 1 in 4300 live births.^2^ The natural course of these diseases is progressive and currently no disease‐modifying therapies are available for the vast majority. Important considerations in the management of patients with mitochondrial diseases include early treatment of organ‐specific complications and avoidance of potential triggers of decompensation including catabolic stressors (eg, fasting, intercurrent illness, pyrexia, trauma, or surgery) or medications that are toxic to mitochondrial function.^3^

For pharmacological treatment of patients risk‐benefit considerations and a search for safer, better tolerated or more effective alternatives are part of normal clinical practice. Prescribing drugs to patients with mitochondrial disease is associated with the additional consideration of the drug's potential to negatively influence mitochondrial function.^4^ Due to the heterogeneity in manifestations of mitochondrial disease, reported effects of a pharmacologic agent in an individual patient do not automatically account for all mitochondrial patients.

Mechanisms of drug‐induced mitochondrial toxicity observed in pre‐clinical models include: (a) inhibition of one or more of the electron transport chain complexes (ETCs); (b) uncoupling of mitochondrial oxidative phosphorylation (OXPHOS) by dissipation of the membrane potential and thus disconnecting the ETC from ATP synthase; (c) inhibition of mitochondrial OXPHOS by binding to ATP synthase; (d) inhibition of mitochondrial protein synthesis and biogenesis by affecting mitochondrial DNA (mtDNA) replication and/or fusion of mitochondria; and (e) formation of reactive oxygen species (ROS) as a consequence of any or all of the four above‐mentioned mechanisms.^5^

Clinical guidelines on the safe use of medications in patients with mitochondrial disease are available but substantial practice variation is a potential source of outcome disparity. Reasons for this have included the paucity of high‐quality evidence and the historical lack of screening for mitochondrial toxicity in drug development. For the vast majority of existing licensed drugs, mitochondrial toxicity is unknown. Thus, when evaluating drug safety in patients with a mitochondrial disease, we must rely on information from in vitro and in vivo pre‐clinical studies and published case reports. Many studies have investigated drug side effects by analysing mitochondrial function in healthy cell lines exposed to high doses of these compounds. It is very difficult to extrapolate these results to what would happen in cells from patients, or what the clinical consequences might be in vivo for patients with mitochondrial disease.

Conscious of the lack of available evidence, we convened a workshop aimed at developing a consensus about safe medication use in patients with a primary mitochondrial disease. Consensus was based on a review of the literature and the clinical experience of paediatricians, internists and neurologists experienced in treating affected patients. The results of this Delphi workshop were used to develop guidance for physicians prescribing drugs for patients with mitochondrial diseases. This article does not contain any studies with human or animal subjects performed by any of the authors.

## 2 METHODS

A modified Delphi‐based technique was used to develop a consensus view of drug safety in patients with a mitochondrial disease by a group of internationally acknowledged experts. The Delphi technique was developed by the RAND (research and development) Corporation in 1953 to elicit and process value judgments.^6^ The technique consists of a structured and repetitive survey of at least two rounds, which continues until consensus is reached among panellists. Between each round, feedback is provided to the panellists.

### 2.1 Formation of panellists

Clinicians and researchers with expertise in mitochondrial medicine and pharmacology were invited. Potential participants were selected based on their known experience in the field of mitochondrial medicine and pharmacology. To broaden the panel of experts, we also asked invitees to provide names of other potential participants. Since drug prescription could vary from country to country and from continent to continent, we endeavoured to achieve geographical balance in the selection of panellists. Candidate panellists were invited by e‐mail outlining the study aims and the Delphi process. Patient representatives from the International Mito Patients advocacy group IMP (https://www.mitopatients.org/) were also invited. The consensus panel finally consisted of 16 participants and two patient representatives from IMP. All the participants are experts in mitochondrial medicine, including 13 clinicians, two pharmacologists and a basic scientist. Participants were from five different European countries and from the USA.

### 2.2 Selection of drugs

Due to the limited duration of the workshop (2 days), only a finite number of drugs could be considered and drugs to be studied were, therefore, selected by using a previously published list on the website of the patient advocacy group IMP (https://www.mitopatients.org/mitodisease/potentially-harmful-drugs) supplemented with a few drugs commonly prescribed to patients affected by mitochondrial disease (eg, anaesthetic agents, analgesics, antibiotics, antiepileptic drugs). Two facilitators (MM and MdV) formulated a list of 32 drugs and drug classes and circulated this to the panellists. Subsequently, panellists had the opportunity to add drugs which were in their view most needed a consensus opinion. Due the limited time, the total number of drugs/drug classes was limited to 46. The decision to study some drugs as a group was based on the fact that the class of drugs have a common pharmacological mode of action or similar off‐target side effects.

### 2.3 Delphi‐based process

**Figure 1:**
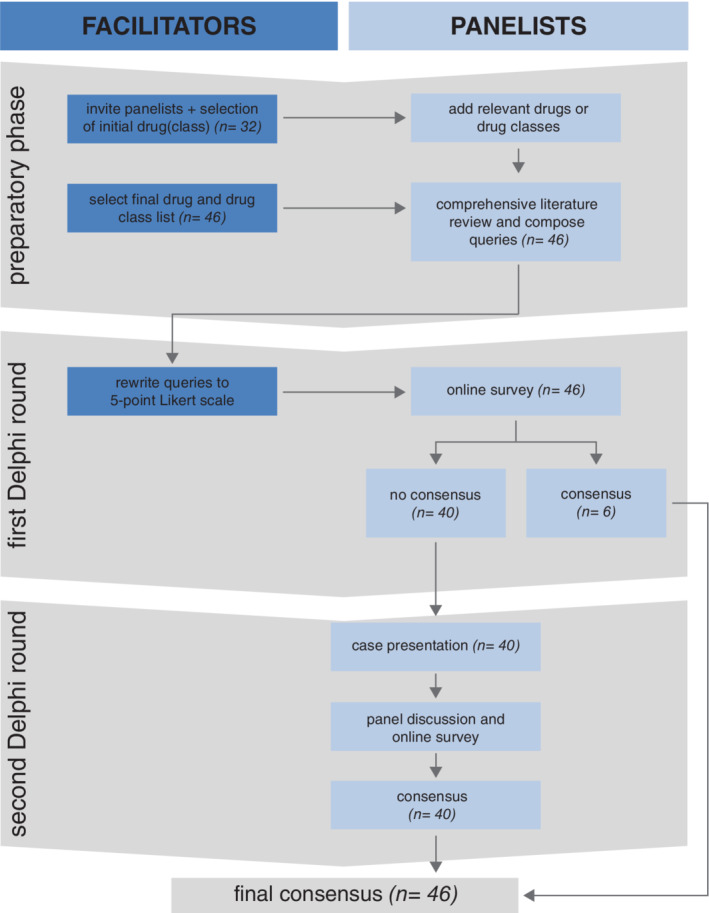
Flowchart of the Delphi‐based process showing the activities and results of the three stages. n = number of drugs/drug groups

The process was performed in three stages (Figure 1).

1. *Preparatory phase*: Potential panellists were invited and the list of drugs and drug classes to be studied was compiled. Each panellist was assigned 2 to 4 drugs or drug classes and was asked to perform a thorough literature search from recognised medical databases. Participants were requested to send a one page summary of their results, including the following items: mode of action; theoretical effect on mitochondrial function; known effects on mitochondrial function; effects observed in patients with mitochondrial disease, cell lines from patients with mitochondrial disease or control cell lines; what type of study (eg, case report, in vitro) and a comprehensive list of references consulted during the review process. The panellists were also asked to submit one or two statements about the drugs they had investigated, including a statement about the safety of the drug in patients with mitochondrial disease.
2. *First Delphi round*: The literature reviews of the drugs were summarised. All panellists were provided with this bundle of drug summaries, as background information, before they were requested to give their opinion in the first voting round. The statements were rewritten by the facilitators and sent to all panellists by an online survey (ie, Google Form), who had the opportunity to indicate their agreement anonymously on a linear five‐point scale anchored at each end by ‘Score 1: Absolutely disagree’ and ‘Score 5: Absolutely agree’. Opportunity was provided for participants to add comments in order to correct areas of ambiguity or suggestions for improvement. Strong consensus agreement was predefined as the mean result ≥4 and ≥70% agreement among the panellists (scores of 4 or 5 on the linear scale). If only one of these two criteria was met, good consensus was considered. If neither consensus criteria was met, then the statement was considered to lack consensus agreement. Statements that did not achieve consensus in the initial round were scheduled for discussion at the workshop as pre‐agreed.
3. *Second Delphi round*: This phase took place during a two‐day workshop. During the workshop, panellists were provided with feedback regarding the drugs for which the statements had not reached consensus. The feedback consisted of presentations of the literature search. After each presentation, there was an opportunity to ask questions, to comment and to exchange personal experiences in prescribing the specific drug in question. At the end of each session during which seven to nine drugs had been discussed, the panellists were asked to re‐rate the statements concerning these drugs by the online survey, that is, the second voting round. Results were analysed to evaluate the level of agreement for each statement. Consensus was pre‐defined as in the first Delphi round.

## 3 RESULTS

### 3.1 Preparatory phase

In the preparatory phase the participants performed and summarised a literature review regarding the drugs or drug groups they were asked to study (see Supplementary data for the summaries of these literature reviews). Furthermore, a total of 55 statements for the voting rounds were composed by the participants to cover the selected 46 drugs/drug groups.

### 3.2 Delphi rounds

All the participants responded in the first Delphi round, all of them participated in the workshop meeting and all of them responded in the second voting round. During the first voting round, prior to the workshop meeting, consensus was not achieved for 40 drugs owing to a low level of agreement (<70% and/or <4). Good or strong consensus was reached for six drugs or drug groups; enalapril, paracetamol, midazolam, carbamazepine, oxcarbazepine, and haloperidol.

According to our pre‐agreed Delphi process, the levels of evidence in the available literature pertaining to drugs that did not reach consensus were discussed during the meeting. The main point of discussion for each drug was the knowledge gap between pre‐clinical drug studies and how these relate to the patient with a mitochondrial disease. Drug effects measured in vitro or in vivo were either not, or not fully, translatable to clinical consequences for the patient for several reasons. Most studies were designed to investigate toxicity of the drug, and consequently examined the effects of extremely high drug doses (ie, to certify the occurrence of a toxic response and associated off‐target mechanisms). Therapeutic drug levels would plausibly show less or no negative effects on mitochondrial function. Moreover, cell lines used in the majority of studies did not have a primary mitochondrial defect and thus none of the compensatory processes associated with mitochondrial dysfunction. Investigating the effects of drugs in cell lines from patients with primary mitochondrial disease was therefore considered worthwhile. In several studies, the formation of ROS was used as an outcome parameter; however, in many cases, the source of the increased ROS concentration, mitochondrial or extra‐mitochondrial, was not demonstrated. After discussing and weighing the pre‐clinical data, the expert team discussed their own clinical experiences of observed safety or side effects of the drug(s) in question in patients with mitochondrial disease.

**Figure 2:**
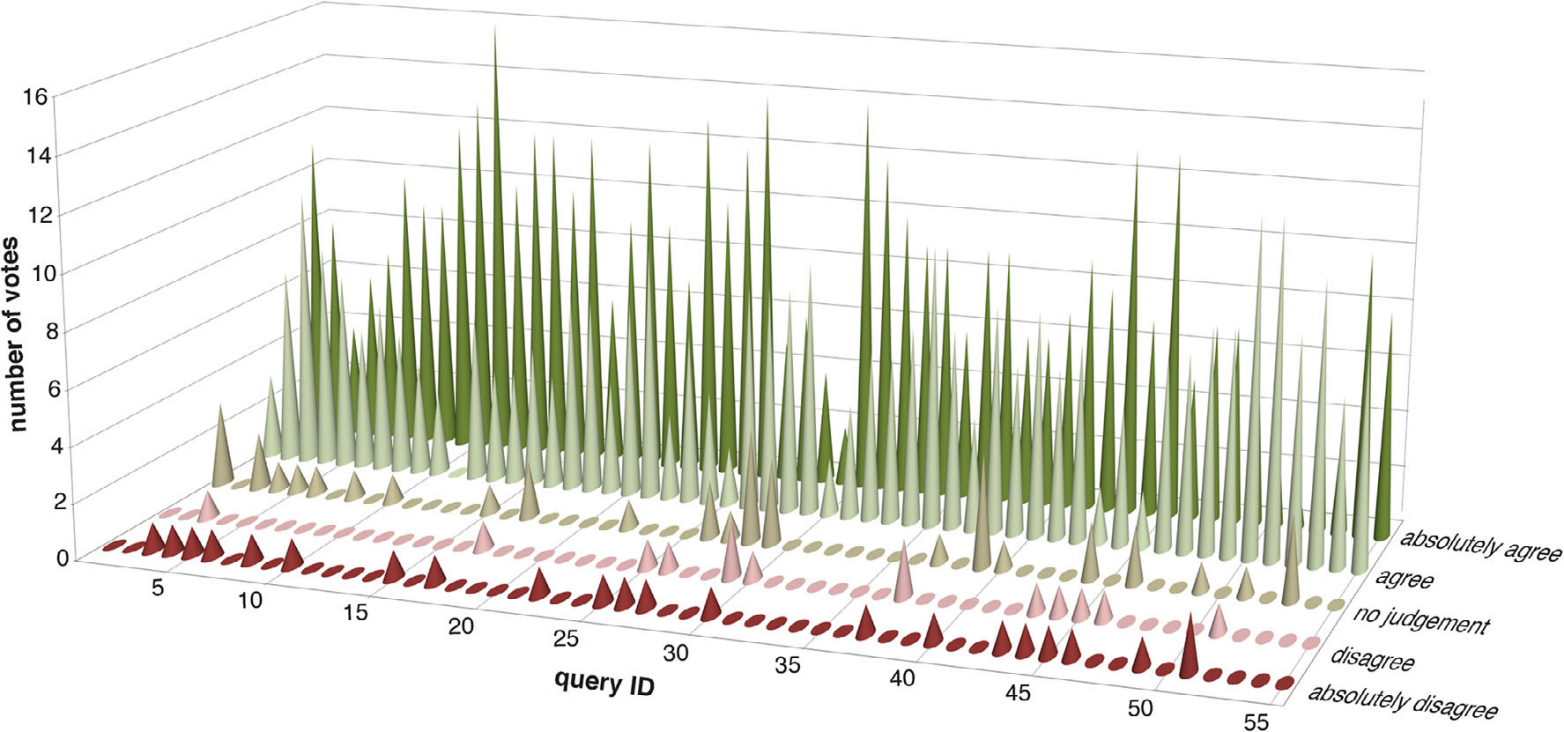
Voting results of the final Delphi round, showing the distribution of votes per statement

This process was followed by the second voting round. Good or strong consensus was achieved for all the drugs or drug groups. The consensus results for all the statements are shown in Table 1 and Figure 2. Immediately following the workshop, it was discussed that one of the statements could have been (for the panellists) or could be (future use of the information by colleagues) phrased misleadingly. It concerned the statement about the use of steroids in patients with primary mitochondrial disease. Therefore, a more clearly defined statement was formulated and the panellists were asked to vote on this statement via an online survey. The result of this voting showed strong consensus.

**Table 1:** Voting results of the final Delphi round per statement

Question	Mean	% of people voting 4 or 5	Consensus
General			
We need to update the IMP table of potentially harmful drugs for mitochondrial patients version 3 (https://www.mitopatients.org/mitodisease/potentially‐harmful‐drugs)	4.87	100	Strong
Good clinical practice including general indications, contraindications, clinical monitoring and side effects for all drugs must always kept in mind (with or without mitochondrial genetic defect). They will not be discussed in this consensus	4.94	100	Strong
For all drugs where clear evidence in vivo of mitochondrial toxicity is absent or poor, they can be used with careful monitoring in the first few days of treatment for potential side effects and measurement of blood lactate	4.19	93.7	Strong
There is a great need for further studies to determine a) the criteria for drug mitochondrial toxicity in humans, and b) which specific drugs are toxic for mitochondria and must be avoided	4.25	75	Strong
Analgesics‐Antipyretics‐NSAIDs‐Corticosteroids			
Paracetamol is not contraindicated in primary mitochondrial disease (PMD)	4.56	87.5	Strong
Do you consider that steroids are safe to use in acutely ill patients with PMD?	4.46	100	Strong
It is safe to use steroids in patients with Kearns‐Sayre syndrome	4.13	86.6	Strong
NSAIDs can be safely used in PMD	4.31	87.5	Strong
It is reasonable to avoid NSAIDs for long periods in PMD with renal or hepatic or gastrointestinal involvement	4.31	93.75	Strong
Use of aspirin is safe in PMD	4.56	93.75	Strong
Alcohol			
Alcohol in large amounts (above recommended daily intake) is generally toxic and should be avoided	4.37	93.75	Strong
Alcohol consumption within the limits recommended by national guidelines appears non‐toxic in PMD	5	100	Strong
Anaesthetics			
It is safe to use articaine in PMD	4.75	100	Strong
It is safe to use bupivacaine in PMD	4.81	100	Strong
It is safe to use lidocaine in PMD	5	100	Strong
It is safe to use volatile anaesthetics in PMD	4.62	100	Strong
It is safe to use fentanyl in PMD	4.75	100	Strong
Ketamine is safe in general anaesthesia for patients with PMD	4.75	100	Strong
Barbiturates are safe in general anaesthesia for patients with PMD	4.56	93.75	Strong
Propofol is safe in induction anaesthesia in PMD	3.81	81.25	Consensus
Extra caution and monitoring should be considered for patients with PMD manifesting predominantly with myopathic phenotype when neuromuscular blockade is required for general anaesthesia and surgery	4.25	87.5	Strong
Non depolarizing neuromuscular blocking agents are safe for general anaesthesia in patients with PMD	4.56	100	Strong
Antibiotics			
As a general approach, short term (< 7 days) antibiotic treatment is unlikely to be a problem in PMD. Infection is a much greater risk than short term antibiotics	4.75	100	Strong
If indicated, linezolid could be used in mitochondrial disease, with careful lactate monitoring, particularly in children and other patients with pre‐existent lactic acidaemia	4.56	100	Strong
It is safe to use quinolones in PMD	4.44	100	Strong
Aminoglycosides should be avoided in patients with predisposing mitochondrial DNA mutations (eg, m.1555A > G and m.1494C > T) for ototoxicity	4.81	100	Strong
Topical chloramphenicol use is safe in PMD	4.62	100	Strong
It is safe to use tetracyclines in PMD	4.75	100	Strong
It is safe to use ceftriaxone in PMD	4.87	100	Strong
Antidepressant‐Neuroleptic drugs			
The use of antipsychotics medications when they are clinically indicated is not contraindicated in PMD	4.19	87.5	Strong
Quetiapine can be safely used in PMD despite some studies in rodents or cell lines indicate potential mitochondrial toxicity	4.31	93.75	Strong
Fluphenazine could be safely used in PMD	4	75	Strong
Haloperidol can be safely used in PMD despite some studies in rodents or cell lines indicate potential mitochondrial toxicity	3.81	75	Consensus
It is safe to use tricyclic antidepressants in PMD	4.87	100	Strong
It is safe to use chlorpromazine in PMD	4.75	100	Strong
It is safe to use clozapine in PMD	4.56	100	Strong
It is safe to use risperidone in PMD	4.56	100	Strong
Antidiabetic drugs			
It is safe to use metformin in PMD	4.56	100	Strong
It is safe to use glitazone in PMD	4.37	100	Strong
Antiepileptic drugs			
Since there are no descriptions of toxicity of midazolam or other benzodiazepines (BDZ) in PMD, it is correct to assume that midazolam or other BDZ could be used in acute seizure in PMD, or be used as anaesthetic	4.56	100	Strong
Valproic acid should be avoided only in POLG patients	4.25	81.25	Strong
In non‐POLG patients with mitochondrial disease, without liver disease, valproic acid could be used to manage refractory epilepsy and refractory mood disorders	4.4	100	Strong
Carbamazepine is safe in PMD	4.12	75	Strong
Oxcarbazepine is not contraindicated in PMD	4.37	93.75	Strong
Oral phenobarbital is safe in patients with PMD	4.6	100	Strong
In refractory mitochondrial status epilepticus, barbiturates in appropriate settings could be used for long duration infusion	4.53	100	Strong
It is safe to use gabapentin in PMD	4.86	100	Strong
It is safe to use phenytoin in PMD	4.33	86.66	Strong
It is safe to use levetiracetam in PMD	4.86	100	Strong
It is safe to use perampanel in PMD	4.13	80	Strong
It is safe to use topiramate in PMD	4.46	100	Strong
In refractory mitochondrial status epilepticus, propofol is safe for long duration infusion (up to 48 hours)	4.47	100	Strong
Ketamine is safe for long duration infusion (eg, refractory status epilepticus) in PMD	4.31	93.75	Strong
Bisphosphonates			
It is safe to use bisphosphonates in PMD	4.25	100	Strong
Cardiovascular drugs			
It is safe to use amiodarone in PMD	4.06	93.3	Strong
It is safe to use beta‐blockers in PMD	4.46	100	Strong
Enalapril is safe in PMD	4.06	81.25	Strong
Fibrate drugs‐Statins			
It is safe to use fibrate in PMD	4.62	100	Strong
It is safe to use statins in PMD as long as guidelines concerning monitoring of CK and symptoms are followed	4.5	100	Strong

Abbreviation: PMD, primary mitochondrial disease.

The general conclusion of the Delphi process, all based on consensus (level 4 evidence), was that all the 46 drugs or drug groups studied are considered to be generally safe for patients with mitochondrial disease although some specific restrictions were considered for certain molecular defects and particular clinical situations. These specific conditions will be reviewed in the Discussion.

## 4 DISCUSSION

In this study, 16 experts in mitochondrial medicine, pharmacology and basic science provided their professional opinion concerning the safe use of medications in patients with primary mitochondrial disease, to assist clinician and patient decision‐making. During the Delphi‐based process, it became apparent that for many drugs clinical experience conferred most weight, since these observations reflected the effect of the drug in actual patients with mitochondrial diseases. It is important to emphasise that consensus‐based information is useful to provide guidance, but that decisions related to drug prescribing should always be tailored to the specific needs and risks of each individual patient. Recognising the need for a personalised drug prescription is essential to avoid the pitfalls of one‐size‐fits‐all clinical standards, especially in patients with a mitochondrial disease, a group characterised by enormous clinical heterogeneity. Moreover, good clinical practice, including general indications, contraindications, clinical monitoring and side effects for all drugs must always be considered, whether it is a patient with mitochondrial disease or not.

Some of the statements devised were genotype‐specific. However, the majority could be applied generically to the disease group (ie, mitochondrial disease) as a whole. Caveats for certain drugs or class of drugs were noted, as follows (Table 2).

1. Screening for mtDNA mutations associated with predisposition to aminoglycoside susceptibility should be considered before treatment with aminoglycosides.^7^ It is strongly recommended to screen for these mtDNA mutations before elective long‐term treatment with aminoglycosides is planned. In some emergency situations aminoglycosides provide very effective broad‐spectrum antibiotic treatment and should be prescribed until immediate danger has passed or microbial sensitivities have been established and a suitable alternative antibiotic can be administered. The benefits of the drug outweigh the risks in these situations. The mitochondrial 12S rRNA is a hot spot for mutations associated with both aminoglycoside‐induced and non‐syndromic hearing loss. Of those, the homoplasmic m.1555A > G and m.1494C > T mutations in the highly conserved coding region of the 12S rRNA have been associated with hearing loss worldwide.^8^
2. Valproic acid should be used only in exceptional circumstances. In most national guidelines for the treatment of status epilepticus, valproic acid is not the first choice drug. Furthermore, the drug is absolutely contraindicated in patients with mitochondrial disease due to *POLG* mutations. Additionally, valproic acid should not be used in patients with known liver disease and/or clinical signs suspicious for *POLG* disease, such as epilepsia partialis continua, explosive onset of focal epilepsy or rhythmic high amplitude delta with superimposed spikes (RHADS) on EEG.^9^
3. The last drug group that deserves particular attention is the group of neuromuscular blocking agents. Extra caution and monitoring should be performed for patients manifesting a predominantly myopathic phenotype.
4. Although historically there have been largely theoretical concerns around general anaesthetic use in patients with mitochondrial disease, adverse events are exceptionally rare. Consensus was unanimous that these drugs and drug classes were deemed safe. General surgery is a potentially risky procedure for any patient with mitochondrial disease. Catabolism should be prevented by minimising preoperative fasting and administering intravenous glucose perioperatively during prolonged anaesthesia, unless the patient is on a ketogenic diet.^3^
5. The duration of drug administration may also play a role in whether not side effects develop. Short term use of midazolam is considered safe, for example, in the acute management of epileptic seizures or for a short anaesthetic procedure. In specific clinical situations, a longer duration of administration of a certain drug can be justified, despite an increased risk of side effects or disease progression. This accounts for situations in which no alternative treatment options are available and the absence of treatment could have a more detrimental effect of disease progression. Examples include the use of propofol or barbiturate infusions in the management of refractory status epilepticus. Duration of treatment should be guided by individual patient needs and their response to specific treatments.
6. Many patients with a mitochondrial disease have renal impairment, including patients with the m.3243A > G mtDNA mutation or genetic defects in *RMND1*.^10^, ^11^, ^12^ Drug dose adjustment should be considered particularly when active drug moieties are renally cleared, for example, levetiracetam.
7. Metabolic acidosis (lactic acidosis) may occur in patients with mitochondrial disease; therefore, drugs that can cause acidosis should be prescribed with caution, with advice to report symptoms of metabolic acidosis and regular clinical review and monitoring of acid‐base status in blood.

**Table 2:** Points of attention regarding drug prescription in patients with a mitochondrial disease (detailed description in Section 4)

Specific drug/drug group/clinical condition/genotype	Points of attention
*Specific drug/drug group/genotype*	
Aminoglycosides	The mitochondrial 12S rRNA is a hot spot for mutations associated with both aminoglycoside‐induced and non‐syndromic hearing loss. Screening for these mtDNA mutations is strongly recommended before elective long‐term treatment is planned. The benefits of the drug in emergency treatment, as a very effective broad‐spectrum antibiotic, outweigh the risks in these situations.
Valproic acid	Should be used only in exceptional circumstances. The drug is absolutely contraindicated in patients with mitochondrial disease due to *POLG* mutations. Valproic acid should not be used in patients with known liver disease and/or clinical signs suspicious for *POLG* disease.
Neuromuscular blocking agents	Extra caution and monitoring should be performed for patients manifesting a predominantly myopathic phenotype.
*Specific clinical condition*	
General anaesthesia and surgery	Catabolism should be prevented by minimising preoperative fasting and administering intravenous glucose perioperatively during prolonged anaesthesia, unless the patient is on a ketogenic diet.
Duration of treatment	The duration of drug administration may play a role in whether or not side effects develop. Duration of treatment should be guided by individual patient needs and their response to specific treatments.
Renal impairment	Many patients with a mitochondrial disease have renal impairment; drug dose adjustment should be considered particularly when active drug moieties are renally cleared.
Metabolic acidosis (lactic acidosis)	Metabolic acidosis (lactic acidosis) may occur in patients with mitochondrial disease, therefore drugs that can cause acidosis should be prescribed with caution. Regular clinical review and monitoring of acid‐base status in blood is recommended.

Abbreviation: PMD, primary mitochondrial disease.

The present study has limitations. Although consensus methods are widely used to inform clinical practice in the absence of empirical data, expert judgement ranks low in the hierarchy of evidence. However, we felt compelled to give transparent opinions based on the available preclinical studies and clinical evidence, to prevent unnecessary withholding of important drugs from patients with primary mitochondrial disease.

A recurrent issue throughout our discussions was the lack of translation between pre‐clinical studies and the clinical situation, where physicians experience that patients appear to tolerate most drugs. Multiple factors will be responsible for this lack of translation, for example, exposure time. We analysed the compounds comparing the *C*_max_ values (ie, in vivo) or concentration ranges (ie, in vitro) of the pre‐clinical exposures employed alongside the *C*_max_ of the clinical dose used and human toxic concentrations. The head‐to‐head comparisons can be seen in Table 3. For only 18 out of 44 drugs or drug classes was the drug concentration employed in rodent or cell models comparable with the therapeutic levels found in clinical populations. This finding demonstrates that the lack of translation to the clinical situation is due to the use of higher or toxic drug levels in the majority of pre‐clinical studies.

**Table 3:** Head‐to‐head comparisons pre‐clinical and clinical drug concentrations of the evaluated drugs

Drug (Class)						Matching preclinical and clincial levels?	References
	Pre‐clinical data			Clinical data			
	Pre‐clinical *C*_max_ or concentration range (μg⋅mL^−1^)	Highest level of complexity evaluated	Model system	*C*_max_ (μg⋅mL^−1^)	Toxicity plasma level (μg⋅mL^−1^)		
*Aminoglycosides* a Gentamycin Tobramycin Amikacin Streptomycin Neomycin	24	In vitro	Primary rat cochlear cells	15‐20	12 12 30 40	Yes	Schulz and Schmoldt^13^; Quan et al^14^; Hodiamont et al^15^
Amiodarone^b^	1.0	In vivo	Isolated liver mitochondria from treated Wistar rats	1.0‐2.5	2.5	Yes	Schulz and Schmoldt^13^
Articaine	28‐284	In vitro	Human leukocytes	0.58		No^c^	Oertel et al^16^; Günaydin and Demiryürek^17^
*Barbiturates* a Amobarbital Pentobarbital Phenobarbital Secobarbital	5.8‐232	In vitro	Isolated liver Mitochondria	2.2‐4.4	5.0 10 30 7.0	No	Schulz and Schmoldt^13^; Dalmora et al^18^; Santos et al^19^
*Beta blockers* a Atenolol Carvedilol Metoprolol Nebivolol Propranolol	8.2	In vitro	H9C2 myocardial cells	0.047	2.0 12 0.48^d^ 1.0	No	Schulz and Schmoldt^13^; Gehr et al, 1999; Sgobbo et al, 2007
*Bisphosphonates* ^*s*^ Alendronate Clodronate Risedronate Ibandronate Zoledronic acid	12‐50 14‐57	In vitro In vitro	Gastric (RGM1) and small intestinal (IEC6) epithelial cells Gastric (RGM1) and small intestinal (IEC6) epithelial cells	0.038 0.00097‐0.0039		No No	Mitchell et al^20^; Yun et al^21^; Nagano et al^22^
Bupivacaine	2.6‐3.9	In vitro	Primary rat cardiomyocytes	0.49‐1.9	2.0	Yes	Schulz and Schmoldt^13^; Li et al^23^; Bethea^24^
Carbamazepine	5.9‐236	In vitro	Isolated rat liver mitochondria	1.5‐6.8	10	Yes	Schulz and Schmoldt^13^; Mahmood and Chamberlin^25^; Santos et al^19^
Ceftriaxone	555	In vitro	Purified rat carnitine/acylcarnitine transporter	223‐276		No	Pochini et al^26^
Chloramphenicol	100	In vitro	Primary human fibroblasts	4.9‐12	25	No	Schulz and Schmoldt^13^
Chlorpromazine	0.32‐1.9	In vitro	Rat ovarian theca cells		1.0	Yes	Schulz and Schmoldt^13^
Clozapine	8.2‐25	In vitro	Mouse myoblasts (C2C12), adipocytes (3 T3‐L1), hepatocytes (FL‐83B) and monocytes (RAW 264.7)	0.10‐0.77	0.6	No	Schulz and Schmoldt^13^
Enalapril	0.0091^e^	In vivo	Isolated cardiac mitochondria from spontaneously hypertensive rats	0.023‐0.21		No	Kelly et al^27^; Holenarsipur et al^28^; Piotrkowski et al^29^; Higuchi et al, 1994
Ethanol	3680‐27 600	In vitro	Human retinal pigment epithelial cells (ARPE‐19)	577^f^	1000	No	Schulz and Schmoldt^13^; Bonet‐Ponce et al^30^; Klockhoff et al^31^
Fentanyl	0.5⋅10^−3^‐2⋅10^−3^	In vitro	Human hepatoma HepG2 cells	0.39⋅10^−3^‐23⋅10^−3^		Yes	Djafarzadeh et al^32^
*Fibrate drugs* a Bezafibrate Ciprofibrate Fenofibrate Gemfibrozil	72‐145	In vitro	Primary fibroblasts and myoblasts from MP	10.6 8.6‐26 29.5		No	Miller and Spence^33^; Abshagen et al^34^; Bastin et al^35^
Fluphenazine	0.043‐44	In vitro	Swiss albino mice brain slices	0.056		No	Balijepalli et al, 1999; Midha et al, 1983
Gabapentin^b^	16‐33	In vivo	Wistar rat striatum mitochondria	4.8	85	Yes	Schulz and Schmoldt^13^; Chen et al^36^;
*Glitazones* Troglitazone Rosiglitazone pioglitazone Ciglitazone	5.5‐22 4.5‐18 4.5‐18	In vitro In vitro In vitro	Human hepatoma cells Human hepatoma cells Human hepatoma cells	0.37‐2.2 0.12‐0.15 0.10‐3.5		No No No	Eckland and Danhof^37^; Loi et al^38^; Balfour and Plosker^39^; Hu et al^40^
Haloperidol^b^	0.0049	In vivo	Brain and muscle mitochondria from treated Sprague‐Dawley rats	0.0076	0.05	Yes	Schulz and Schmoldt^13^; Barrientos et al^41^; Lei et al,^42^; Desai, et al^43^
Halothane	18‐395	In vitro	Isolated pig heart mitochondria	90‐225^g^		Yes	Hanley et al^44^; Atallah and Geddes^45^
Ketamine	5.5‐17	In vivo	Brain mitochondria from treated Wistar rats	0.042	7.0	No	Venâncio et al^46^; Wellington et al^47^; Moaddel et al^48^; Yanagihara et al^49^; Schulz and Schmoldt^13^
Lidocaine	234‐2340	In vitro	Rat dorsal root ganglion	0.157‐0.552	6.0	No	Onizuka et al^50^; Schulz and Schmoldt^13^
Linezolid	5‐15	In vitro	Mouse neurons	12.5	ND	Yes	Bobylev et al^51^
Metformin	50‐1292	In vitro	Isolated mouse skeletal muscle mitochondria and various cell lines (ie, NT2196, NMuMG, MFC10A, and MCF7)	1.3	5.0	No	Schulz and Schmoldt^13^; Shu et al^52^; Andrzejewski et al^53^
Midazolam	33‐326	In vitro	Isolated rat and skeletal muscle mitochondria	0.15	1.0	No	Schulz and Schmoldt^13^; Link et al^54^; Colleoni et al^55^
*NSAIDs* a Diclofenac Ibuprofen Indomethacin Naproxen Celecoxib	18‐35	In vitro	Isolated duodenum mitochondria	4.2 96	50 200 4.0 200	No	Schulz and Schmoldt^13^; Sandoval‐Acuña et al^56^; Caille et al^57^
Oxarbazepine	76	In vitro	Rat embryo Hippocampal neurons	1.1		No	Araújo et al,^58^; Tartara et al^59^
Paracetamol	756	In vitro	Isolated mouse liver mitochondria and primary hepatocytes	18‐21	100	No	Schulz and Schmoldt^13^; Sevilla‐Tirado et al^60^; Burcham and Harman^61^
Phenytoin	6.3‐252	In vitro	Isolated rat liver mitochondria	2.0		No	Santos et al^19^; Suthisisang et al^62^
Propofol	4.5‐18	In vitro	Isolated rat liver mitochondria	2.1‐29^h^		Yes	Branca et al^63^; Khan et al^64^
Quetiapine	9.6‐77	In vitro	Isolated rat liver mitochondria	53‐117	1.8^d^	Yes	Schulz and Schmoldt^13^; Modica‐Napolitano et al^65^; DeVane and Nemeroff^66^
Risperidone	10‐82	In vitro	Isolated rat liver mitochondria	20‐60		Yes	Modica‐Napolitano et al^65^; Heykants et al^67^
*Salicylates* a Acetylsalicylic acid Salsalate	90‐1802	In vitro	Isolated subsarcolemmal mitochondria	1.0‐4.8	300 300	No	Schulz and Schmoldt^13^; Nulton‐Persson et al^68^; Nagelschmitz et al^69^
*Statins* a Atorvastatin Lovastatin Pravastatin Rosuvastatin Simvastatin	16‐56 13‐41 36‐42 25‐48 19‐84	In vitro	Murine myoblasts (C2C12)	27‐66 10‐20 45‐55 37 10‐34		Yes Yes Yes Yes Yes	Bellosta et al^70^; Schirris et al^71^
Topiramate^b^	18‐90	In vivo	Isolated mitochondria from treated Sprague‐Dawley rats	3.7‐7.7		No	Kudin et al^72^; Doose et al^73^; Matar and Tayem^74^
Valproic acid	3.6‐29	In vitro	Isolated rat liver mitochondria	98‐113	150	No	Schulz and Schmoldt^13^; Nunez et al^75^; Jafarian et al^76^

*Note:* The concentration range used in in vitro studies (eg, cellular studies) or peak plasma concentrations (*C*_max_) of animal studies were compared with the corresponding human peak plasma concentrations of drugs evaluated. When no peak plasma concentrations were available, they were calculated using standard pharmacokinetic equations (ie, *C*_max_ = *F* × *D*/*Vd*) or obtained from studies using the same dosing regimen and for animal studies the same species and strain. For pharmacokinetic calculations an average rat weight of 190 mg was assumed.

Abbreviation: ND, no data available.

a For drug classes, up to five representative members were selected based on UpToDate. Waltham, MA: UpToDate Inc. https://www.uptodate.com (Accessed on May 6, 2019).

b Pre‐clinical *C*_max_ values were calculated using available pharmacokinetic data.

c Plasma concentrations after subcutaneous articaine injection. Low plasma levels are expected as this drug is intended for local anaesthesia where much higher concentrations can be reached.

d Based on a single case‐report.

e Total free enalapril concentrations, *C*_max_ including the main metabolite: 1.49 μg⋅mL^‐1^.

f *C*_max_ after consumption of one alcoholic 20% (*v/v*) drink.

g Indicated concentrations show the clinical plasma concentration range, not *C*_max_.

h No *C*_max_, but concentration at loss of consciousness.

Limitations were noticed as well in drawing recommendations from published clinical series and case reports, due to several factors. Many studies describe patients with a biochemical defect in OXPHOS without mentioning a specific mitochondrial genetic defect, that is, without confirmation of primary mitochondrial disease. Furthermore, patients often have co‐morbidities and/or are using polypharmacy making it impossible to determine whether the effects reported are directly related to the drug in question or are consequences of one of the other diseases or other drugs.

We plan to revise the existing list regularly and to extend the list of drugs in the future using the same Delphi process. The list of drugs studied will be freely available on the web pages of centres of mitochondrial disease expertise and the IMP. This provides the opportunity to easily revise and update knowledge about the included drugs. Furthermore, given the frequent disparities between pre‐clinical studies and clinically relevant treatment doses, future pre‐clinical toxicity studies that more closely model patient doses and conditions (ie, mitochondrial deficiencies) are needed for improved drug‐induced mitochondrial dysfunction assessments in mitochondrial patients.

## 5 CONCLUSION

This study details the consensus guidelines of a mitochondrial expert panel on the safe use of medications in patients with mitochondrial disease. We acknowledge that the quality of available evidence or published literature used to accredit these recommendations is currently limited. The key recommendations are: (a) valproate should be avoided in patients with *POLG*‐related mitochondrial disease and alternative anticonvulsants considered as first‐line therapeutic strategies; (b) prolonged use of specific drugs may have negative consequences and should be avoided if good alternative treatment options are available; and (c) the usual standards of good practice prevail when prescribing any drug, irrespective of the drug's mitochondrial toxicity potential or profile. We do emphasise that drug prescriptions should always be tailored to the individual patient after careful consideration of their specific needs.

## CONFLICT OF INTEREST

M. A. reports grants from Virginia Tech Interdisciplinary Graduate Education Program, outside the submitted work.

D. B. reports grants from National Institutes of Health, grants and personal fees from Stealth BioTherapeutics, grants from vTv Therapeutics, grants from Catabasis Pharmaceuticals, grants from United States Dept of Agriculture, outside the submitted work.

A. K. reports that she is the current President of the Mitochondrial Medicine Society.

R. M. reports personal fees from Eisai, outside the submitted work.

S. R. reports personal fees from BioMedical, personal fees from NeuroVive, personal fees from Partners 4 Access, outside the submitted work, and that she is an Editor of the Journal of Inherited Metabolic Disease.

T. S. reports grants from Princes Beatrix Muscle Foundation (Prinses Beatrix Spierfonds), grants from European Molecular Biology Organization, during the conduct of the study.

L. B., G. G., N. K., C. L., M. M., R. M., Y. S. N., M. O., R. P., F. R., K. V., and M. C. De V. declare that they have no conflict of interest.

## AUTHOR CONTRIBUTIONS

All authors contributed to the acquisition of data and preparation of critical revision of manuscript.

M. C. De V. and M. M. contributed to the study conception and design, and analysis and interpretation of data.

All authors except M. E. A. contributed to workshop participation.

M. C. De V., M. M., D. A. B., M. E. A., and T. J. J. S. contributed to drafting of manuscript.

## Supporting information

Data S1: Supplementary data
