# Supplementary material for: Safety of drug use in patients with a primary mitochondrial disease: An international Delphi‐based consensus
Source: J Inherit Metab Dis. 2020 Feb 7;43(4):800–18. doi: 10.1002/jimd.12196 (PMC7383489; doi:10.1002/jimd.12196)
Supplement: Supplementary file 1 — Data S1: Supplementary data [file JIMD-43-800-s001.docx]

| **Alcohol** |
| --- |
| **Mode of action** |
| Ethyl alcohol (ethanol; CH_3_ -CH_2_ -OH) is a low molecular weight hydrocarbon that is rapidly absorbed with peak concentration 20-60 minutes after ingestion. It is converted to acetaldehyde and conversion involves 3 enzymes: the microsomal cytochrome P450 isoenzyme CYP2E1, the cytosolic enzyme alcohol dehydrogenase (ADH), and the peroxisome catalase system. Acetaldehyde, which is the active/toxic component, is converted to acetate in mitochondria, thereafter to acetyl CoA, and lastly to carbon dioxide and water.  Genetic polymorphisms in the gene encoding alcohol dehydrogenase appear to control the rate at which ethanol is metabolised. A general rule is that ethanol is metabolized at a rate of 20-25 mg/dL in the non-alcoholic, and faster in chronic alcoholics. Side effects: intoxication, flushing, hypotension, agitation, hypoglycaemia, nausea, vomiting, polyuria. |
| **Effects measured/seen in mitochondrial patients, mitochondrial cell lines or normal cell lines** |
| Evidence is clear that overconsumption of alcohol causes:   1. Liver damage 2. Cardiomyopathy 3. Myopathy 4. And other (undesirable) effects   Acetaldehyde (Ac) can inhibit the mitochondrial respiratory chain (MRC). One study looked at this in rat liver mitochondria at doses ranging from 20-80µM and found progressive impairment of respiratory rate (state3/state 4 respiration (Farfan Labonne et al 2009). *Note: Breath acetaldehyde levels are about 10-20 and 20-40nM at blood ethanol concentrations of about 10 and 20mM, respectively. Theoretically calculated corresponding blood acetaldehyde levels in pulmonary blood would be about 2-4 and 4-8 microM (Eriksson 2007). Blood concentration of 20mM = 0.092 mg/100ml (Slight impairment of balance, speech, vision, reaction time, and hearing. Euphoria. Reduced judgment and self-control. Impaired reasoning and memory.).*  Several effects on mitochondrial function are possible including decreased ATP production, structural changes in heart/liver mitochondria and decreased respiration rate, but my reading of this is that studies have mostly used levels that would be consistent with intoxication (Manzo-Avalos and Saavedra-Molina 2010). Interestingly, however, a study of long term alcoholics showed no effect on MRC function (Cardellach et al 1992). Ac does also appear to damage DNA as exemplified by Fanconi’s anaemia. Disease causing mutations in ALDH2 cause this syndrome and Ac is toxic for these (Joenje 2011).  General mitochondrial disease  There is one paper that presents data on two sisters with a mitochondrial myopathy characterised by weakness, marked exercise intolerance and a fluctuating lactic acidaemia. Both patients also experienced episodes of increased weakness which could be brought on by unaccustomed activity, going without food or by taking small quantities of alcohol. (Morgan-Hughes et al 1979). The defect in these patients is, as far as information from the original authors is gathered, still unknown.  LHON  Here the evidence is suggestive but not absolutely convincing. In a recent study, Kirkman et al (Kirkman et al 2009) showed that there was a trend toward increasing visual failure in LHON, but only in those with heavy intake (was quantified as the maximum units of alcohol consumed in a single week). Another report documented a patient who improved after stopping alcohol consumption (Morris and Votruba 2012), but this was 10 months after symptoms started and well within the natural history of LHON (m14484T>C). Unlike tobacco (Caporali et al 2017) which appears to be a clear risk, alcohol is not clearly a toxic factor for LHON, at least in small/moderate amounts.  Tobacco/alcohol amblyopia (TAA): there are many toxins that can can cause optic neuropathy (Grzybowski et al 2015), however, the evidence for alcohol alone is not consistent. The disease once called TAA is no longer thought to exist. It is possible that smoking, and cyanide exposure, is the cause, but the role of alcohol is unclear and several deny the existence of the condition (Grzybowski and Pieniazek 2014). |
| **References** |
| Caporali L, Maresca A, Capristo M, et al (2017) Incomplete penetrance in mitochondrial optic neuropathies.  *Mitochondrion* 36: 130-7.  Cardellach F, Galofré J, Grau JM, et al (1992) Oxidative metabolism in muscle mitochondria from patients  with chronic alcoholism. *Ann Neurol* 31: 515-8.  Eriksson CJ (2007) Measurement of acetaldehyde: what levels occur naturally and in response to alcohol?  *Novartis Found Symp* 285: 247-255; discussion 256-260.  Farfan Labonne BE, Gutierrez M, Gomez-Quiroz LE, et al (2009) Acetaldehyde-induced mitochondrial  dysfunction sensitizes hepatocytes to oxidative damage. *Cell Biol Toxicol* 25: 599-609.  Grzybowski A, Pieniazek M (2014) Tobacco-alcohol amblyopia does not exist. *Acta Ophthalmol* 92: e77-78.  Grzybowski A, Zulsdorff M, Wilhelm H, Tonagel F (2015) Toxic optic neuropathies: an updated review. *Acta*  *Ophthalmol* 93: 402-410.  Joenje H (2011) Metabolism: alcohol, DNA and disease. *Nature* 475: 45-6.  Kirkman MA, Yu-Wai-Man P, Korsten A, et al (2009) Gene-environment interactions in Leber hereditary optic  neuropathy. *Brain* 132: 2317-26.  Manzo-Avalos S, Saavedra-Molina A (2010) Cellular and mitochondrial effects of alcohol consumption. *Int J*  *Environ Res Public Health* 7: 4281-304.  Morgan-Hughes JA, Darveniza P, Landon DN, Land JM, Clark JB (1979) A mitochondrial myopathy with a  deficiency of respiratory chain NADH-CoQ reductase activity. *J Neurol Sci* 43: 27-46.  Morris B, Votruba M (2012) Leber's optic neuropathy - visual return on alcohol cessation. *Acta Ophthalmol* 90: e568. |

| **Aminoglycosides** |
| --- |
| **Mode of action** |
| Binds to bacterial 30S ribosomal subunit preventing bacterial protein synthesis (1). It is particularly effective in treating gram negative infections. |
| **Known patterns of toxicity** |
| Nephrotoxicity. Aminoglycosides reach high concentrations in the proximal convoluted tubule and degree of toxicity is related to duration of treatment (2). It is pinocytosed and accumulates within lysosomes (3, 4) which eventually rupture (5) releasing hydrolases and the drug to other organelles including uptake to mitochondria (6, 7). Tubular cell necrosis is seen histologically. Clinical features include aminoaciduria/proteinuria, diminished GFR and nephrogenic DI (4). Biochemically, aminoglycosides are thought to alter the plasma membrane of renal epithelial cells, and cause mitochondrial and lysosomal dysfunction (4,8,9,10)  Ototoxicity. Aminoglycosides are endocytosed and can remain sequestered in (11) and cause damage to the hair cells of the organ of Corti (11,12,13) and stria vascularis cells of the cochlear duct which have high mitochondrial activity/energy requirements in order to maintain ion gradients in the inner ear (14). Large pedigree studies have shown that m.1555A>G (12S rRNA gene) (14,15,16,17,18) is associated with an increased susceptibility to aminoglycoside induced deafness due to enhanced binding of the drug to rRNA contained in mitoribosomes) and ultimately mistranslation of multiple mtDNA encoded protein subunits (14,19). Furthermore m.961T>Cn (20), m1494C>T (18,21,22), m.827A>G (23), m.1095T>C (24) have also been associated with aminoglycoside ototoxicity. m.7445A>G (tRNA Ser gene) may be associated with otoxicity also. (25). Ototoxicity may also be seen in patients receiving aminoglycosides without these mutations (26). |
| **Theoretical effects on mitochondrial function** |
| 1. Binds the 28S subunit of rRNA in mitoribosomes and causes toxicity by inhibiting mitochondrial protein translation (11,14,21,27,28, 29) 2. Reduced respiration or ATP levels (4,8,21,28,30,31,32, 33,34, 35,36) 3. Competition with Mg at the inner mitochondrial membrane increasing mito monovalent cation permeability (4) 4. Altered mitochondrial morphology eg swelling and disrupted cristae (12, 37,38,39,36) 5. Increased ROS production/reduced antioxidant pathways (11,24,28,32,33,35,39,40,41,42, 43,44,45,46,47,48,49) 6. Reduced Complex I activity (33,35,45,50), reduced complex II activity (34,35,45,50) reduced complex II/III activity (24,50) reduced complex IV activity (35,45,50), complex V activity (36) 7. Reduced mitochondrial membrane potential MMP (28,30,36,39,46,47,48) 8. Mitochondrial permeability transition (33,51) 9. Apoptosis (10,11,24,35,38,39,43,44,45,46,47,48,49,50,51, 52,53 ,54,55,56) 10. Carnitine deficiency (32) 11. Mitophagy (28) (contradicted by 57) |
| **Known effects on mitochondrial function** |
| \| Type of Study \| Model Used \| Effect Measured \| Effect seen/ Mechanism \| Ref \| \| --- \| --- \| --- \| --- \| --- \| \| In vivo \| Mice, isolated mouse renal mitochondria  Gentamicin exposure \| O2 consumption  Renal function (BUN) \| Reduced O2 consumption  Increased BUN \| 8 \| \| In vivo \| Chick embryos, hair cells  Kanamycin/Netilmicin exposure \| Mito morphology \| Mito swelling, disruption of cristae in hair cells, moreso for kanamycin than netilmicin. \| 12 \| \| In vitro \| Human cybrids with m.1494C>T  Paromomycin exposure \| O2 consumption  Mito protein synthesis \| Cybrids containing m.1494C>T mutation(lymphoid cell mitochondria from affected individuals fused with mtDNA -less p^o^206 cells) had reduced mito protein synthesis(radiolabelled Met uptake) and O2 consumption rates, and grew more slowly on exposure to paromomycin \| 21 \| \| In vitro \| Human Cybrid cells with m. 1095 T>C mutation  Gentamicin exposure \| Mitochondrial GSH, CII/CII, caspase 3 \| Cybrids with m.1095 T>C made from lymphoid cells of affected individuals and mtDNA – less p^o^206 cells showed reduced mitochondrial GSH, 30% reduction in CII/II activity. Evidence of apoptosis with high caspase 3 expression. \| 24 \| \| In vitro \| Human cybrids with m.1494C>T  Gentamicin exposure \| MT-CO1, MTCO2, MT-ND5 gene products, ATP levels, MMP, ROS, mitophagy. \| Cybrids containing m.1494C>T mutation (lymphoid cell mitochondria from affected individuals fused with mtDNA -less p^o^206 cells) had reduced MT-CO1, MTCO2, MT-ND5 translation, ATP levels, reduced MMP, elevated total cell ROS and increased mitophagy \| 28 \| \| In vitro \| Isolated rat liver mitochondria  Neomycin exposure \| Mitochondrial Ca  O2 consumption \| Reduced mitochondrial calcium  Reduced O2 consumption \| 30 \| \| In vivo \| Rats, renal cortex  Gentamicin exposure \| Histology, cortical cell proteome \| Large derangements in numerous enzymes with high dose gentamicin (70mg/kg/d for 4d) including Kreb cycle enzymes and CV downregulation. Cortical cell necrosis seen histologically. \| 31 \| \| In vivo \| Rats  Gentamicin exposure \| Renal function, urinalysis, intramitochondrial acetyl-CoA, free carnitine, ATP levels, GSH levels \| Increased serum Cr, BUN, urine free carnitine losses, increased intramitochondrial acetyl-CoA. Reduced renal cell total free carnitine, ATP, GSH,ATP/ADP ratio. L carnitine supplementation was able to ameliorate these effects. \| 32 \| \| In vivo \| Rats, isolated rat kidney mitochondria  Gentamicin exposure \| Renal function  Total antioxidant status, H2O2 levels  Histology  MTP and cyt c compartmentalisation  O2 consumption  NADH level, CI activity \| Reduced Cr clearance, increased NAG, lower GFR  Reduced total antioxidant status, Elevated H2O2 levels in mitochondria  Massive and diffuse proximal tubular necrosis, tubules filled with hyaline casts and cellular debris.  Increased MTP and cytochrome c release to cytosol  Reduced O2 consumption  Reduced NADH and complex I activity \| 33 \| \| Ex vivo \| Mouse intact organ of Corti Explants  Gentamicin exposure \| NADH fluorescence, SDH activity, ATP levels \| NADH ffluorescence, SDH activity, ATP levels are reduced in outer high frequency hair cells while inner hair cells were undisturbed. \| 34 \| \| In vivo \| Rats  Gentamicin exposure \| Renal function urine protein levels, histology, complexes I,II, IV  apoptotic markers caspase-3 and bax  GSH, ATP, SOD levels \| Elevated BUN, Cr in serum, proteinuria  Decreased SOD activity and GSH.  Elevated Bax and caspase 3  Reduced CI, CII, CIV activity  Reduced ATP  Histology shows tubular degeneration, hyalinecases, monocellular infiltrates and intraluminal cellular debris \| 35 \| \| In vivo \| Rats, isolated rat renal mitochondria  Gentamicin exposure \| Renal function, O2 consumption, MMP, Mito morphology, CV activity, \| Elevated Cr, BUN, reduced GFR, mildly reduced MMP, reduced CV activity, Reduced O2 consumption rate, mito swelling seen, renal cortical necrosis \| 36 \| \| In vivo \| Rats  Gentamicin exposure \| Histology \| Degeneration of proximal convoluted tubular cells, Swollen mitochondria and enlarged lysosomes. Reversed after cessation of drug \| 37 \| \| In vivo \| Minipig model  Gentamicin exposure \| TUNEL assay, renal function, EM, caspase3, inflamm cytokines \| Elevated BUN and Cr, swelling/ disintegration of cristae, increased caspase 3, elevated inflammatory cytokines \| 38 \| \| In vivo \| Rats  Gentamicin exposure \| Renal function  Lipid peroxidation (ROS)  Catalase/SOD activity, GSH, caspase 3 expression, Bcl-2/bax ratio, MMP, cyt c release, mito morphology \| Reduced renal function  Increased ROS, Reduced activity of antioxidant enzymes(catalase, SOD), reduced GSH, increased proapopotic state (increased caspase 3, Bcl2/Bax ratio), reduced MMp, mitochondrial swelling and cyt c release \| 39 \| \| In vitro \| Isolated rat renal cortical mitochondria  Gentamicin exposure \| ROS(H2O2) production \| Increased ROS(H2O2) production on exposure to even low [gentamicin] of 0.01mcM. \| 40 \| \| In vitro \| Isolated rat renal cortical mitochondria  Gentamicin exposure \| ROS production \| Increased ROS(superoxide and hydroxyl anion) production on exposure to even low [gentamicin] \| 41 \| \| In vivo \| Rats  Gentamicin exposure \| Renal function, urinalysis, antioxidant enzyme activities \| Elevated serum creatinine, BUN, massive proteinuria, glycosuria, urinary electrolyte losses. Reduced enzyme activity of antioxidant enzymes SOD, catalase, glutathione peroxidase \| 42 \| \| In vitro \| HEI-OC1 cell line (derived from mouse cochlear cells)  Gentamicin exposure \| MTT assay  Intracellular ROS measurement  Caspase 3 \| Reduced cell viability, increased ROS and caspase 3 protein expression \| 43 \| \| In vivo \| Guinea Pigs  Gentamicin exposure \| Auditory brain responses, EM, Mn SOD expression, Bak (proapoptotic protein expression) \| Gentamicin exposed animals had hearing loss compared to baseline levels.  EM showed hair cell death in the basal turn of the cochlea, Bak was expression elevated, MnSOD reduced. \| 44 \| \| In vivo \| Rats, isolated rat renal mitochondria  Gentamicin exposure \| Renal function, histology, renal ROS, GSH, SOD activity, CI,CII,CIV activity, caspase 3 and 9, Bax, p52, bcl-2 expression, inflammatory cytokines \| Elevated BUN, Creatinine, histology showed tubular degeneration, tubular necrosis and inflammatory cell infiltration  Reduced levels of GSH, elevated lipid peroxidation, reduced SOD activity  Reduced CI, CII, CIV activity  Elevated inflammatory cytokines, including IL6 and TNFa  Elevated proapoptotic factors including caspase 3 and 9, Bax, p53. Reduced Bcl-2 expression \| 45 \| \| In vitro/ex vivo \| HEI-OC1 cells  Mouse Cochlear explants  Gentamicin exposure \| Cell viability(MTT assay), hair cell number, cell morphology, ROS, MMP, apoptosis(PI stain), Bax, Bcl-2, caspase 3 mRNA expression \| Reduced cell viability, increased ROS, reduced MMP, increased hair cell apoptosis, increased expression of proapoptotic gene mRNA transcripts(Bax, caspase 3) and reduced Bcl-2 \| 46 \| \| Ex vivo \| Mouse cochlear explants  Amikacin exposure \| Histology, caspase 3 expression, TUNEL assay, ROS, MMP \| Degeneration of outer hair cells and stereocilia bundles, elevated ROS, elevated caspase 3 expression and TUNEL labelled cells, decreased MMP. \| 47 \| \| Ex vivo \| Rat cochlear explants  Gentamicin exposure \| Hair cell viability, caspase 3 expression, ROS, MMP \| Reduced hair cells and increased caspase 3 expression, increased ROS, decreased MMP. \| 48 \| \| In vivo  Ex vivo \| Mice, mouse cochlear explants  Neomycin exposure \| ABRs, hair cell viability, TUNEL assay, apoptosis mRNA and protein expression, ROS \| ABR thresholds increased, loss of hair cells, increased TUNEL pos (apoptotic) cells, increased Caspase 3,9, Bax and Apaf1(proapoptotic) mRNA and protein expression, reduced Bcl-2 expression. Increased ROS \| 49 \| \| In vivo \| Rats  Gentamicin exposure \| Renal function complexes I -IV  Bcl-2 mRNA expression  GSH, SOD levels  Inflammatory cytokines \| Elevated BUN, Cr in serum,  Decreased SOD activity and GSH.  Reduced Bcl-2 mRNA expression  Reduced CI-CIV activity  Inflammatory cytokines NFKb, KIM1, NGAL elevated \| 50 \| \| In vitro \| Guinea pig cochlea  Gentamicin exposure \| Cell viability  MMP \| Reduced cell viability within 2h of exposure (outer hair cells affected more than inner hair cells) and reduced MMP, reversed by cyclosporin A implying there is a role for the MPT. Apoptosis also seen. \| 51 \| \| In vitro \| Mouse hair cells  Neomycin exposure \| Caspase 3,8, histology \| Apoptosis demonstrated on TUNEL and TEM, elevated caspase 3 and 8. \| 52 \| \| In vivo \| Zebrafish  Neomycin exposure \| Apoptosis (TUNEL assay), EM, DASPEI to stain mitochondria of hair cells \| Neomycin caused apoptosis (TUNEL pos hair cells), EM showed severe morphological damage of hair cells and loss/fusion of stereocilia. \| 54 \| \| In vitro \| HEI-OC1 cells  Gentamicin exposure \| Apoptosis  Expression of proapoptotic proteins Bak \| Elevated expression of Bak and increased apoptosis \| 55 \| \| In vitro \| HEI-OC1 cells  Streptomycin exposure \| Cell viability (MTT assay), A1F mRNA and protein expression \| Elevated A1F (proapoptotic factor), cell loss on MTT assay \| 56 \| \| In vitro \| Isolated rat renal cortical mitochondria  Gentamicin exposure \| ROS production \| Increased ROS(superoxide and hydroxyl anion) production \| 58 \| \| In vitro \| Rabbit renal proximal tubular cells  Gentamicin exposure \| O2 consumption rate \| Reduced O2 consumption rate \| 59 \| \| In vitro \| LLC-PK1 (porcine kidney prox tubule)  BGM(renal cells from african green monkey)  Gentamicin exposure \| Cytotoxicity measured by MTT absorbance assay (mito hydrolase dependent) \| Reduced viable cells with increasing [gentamicin]. Results also indicate mitochondrial dysfunction \| 60 \| \| In vivo/  in vitro/  ex vivo \| Rats, rat cochlear hair cells and explants  Gentamicin exposure \| ABRs, ROS, apoptosis (TUNEL) assay \| Reduced number of hair cells, increased apoptotic cells, increased ROS, deterioration in ABRs especially after 7d of gentamicin exposure. \| 61 \| \| In vitro \| HEI-OC1 cells  Gentamicin exposure \| Mitophagy, O2 consumption \| Mitophagy not induced – contradicts (99)  No change to O2 consumption \| 62 \| |
| **References** |
| 1. Kouzaki H, Suzuki M, Shimizu T (2007) Immunohistochemical and ultrastructural abnormalities in muscle from a patient with sensorineural hearing loss related to a 1555 A-to-G mitochondrial mutation. *J Clin Neurosci* 14: 603-7. 2. Fabre J, Fillastre JP, Morin JP et al (1978) Nephrotoxicity of gentamicin Action on subcellular organelles and pharmacokinetics in the kidney. *Contrib Nephrol* 10: 53-62. 3. Silverblatt FJ, Kuehn C (1979) Autoradiography of gentamicin uptake by the rat proximal tubule cell. *Kidney Int* 15: 335-45. 4. Humes HD, Weinberg JM, Knauss TC (1982) Clinical and pathophysiologic aspects of aminoglycoside nephrotoxicity. *Am J Kidney Dis* 2: 5-29. 5. Kaloyanides GJ (1992) Drug-phospholipid interactions: role in aminoglycoside nephrotoxicity. *Ren Fail* 14: 351-7. 6. Vera-Roman J, Krishnakantha TP, Cuppage FE (1975) Gentamicin nephrotoxicity in rats: Acute biochemical and ultrastructural effects. *Lab Invest* 33: 412-7. 7. Steyger PS, Peters SL, Rehling J et al (2003) Uptake of gentamicin by bullfrog saccular hair cells in vitro. *J Assoc Res Otolaryngol* 4: 565-78. 8. Humes HD, Sastrasinh M, Weinberg JM (1984) Calcium is a competitive inhibitor of gentamicin-renal membrane binding interactions and dietary calcium supplementation protects against gentamicin nephrotoxicity. *J Clin Invest* 73: 134-47. 9. Kaloyanides GJ (1994) Antibiotic-related nephrotoxicity. *Nephrol Dial Transplant* Suppl 4:1 30-4. 10. Torres-Ruíz NM, Granados O, Meza G (2011) Aminoglycosides: therapeutics, ototoxicity and hypersensitivity of mitochondrial genetic origin. *Proc West Pharmacol Soc* 54: 49-51. 11. Warchol ME (2010) Cellular mechanisms of aminoglycoside ototoxicity. *Curr Opin Otolaryngol Head Neck Surg* 18: 454-8. 12. Fermin CD, Igarashi M (1983) Aminoglycoside ototoxicity in the chick (Gallus domesticus) inner ear: The effects of kanamycin and netilmicin on the basilar papilla. *Am J Otolaryngol* 4: 174-83. 13. Anniko M (1983) Aspects on the ototoxic potential of netilmicin. *Acta Otolaryngol* 96: 75-89. 14. Hutchin T, Cortopassi G (1994) Proposed molecular and cellular mechanism for aminoglycoside ototoxicity. *Antimicrob Agents Chemother* 38: 2517-20. 15. Pandya A, Xia X, Radnaabazar J et al (1997) Mutation in the mitochondrial 12S rRNA gene in two families from Mongolia with matrilineal aminoglycoside ototoxicity. *J Med Genet* 34: 169-72. 16. Gardner JC, Goliath R, Viljoen D et al (1997) Familial streptomycin ototoxicity in a South African family: a mitochondrial disorder. *J Med Genet* 34: 904-6. 17. Van Camp G, Smith RJ (2000) Maternally inherited hearing impairment. *Clin Genet* 57: 409-14. 18. Gao Z, Chen Y, Guan MX (2017) Mitochondrial DNA mutations associated with aminoglycoside induced ototoxicity. J *Otol* 12:1-8. 19. Jacobs HT (1997) Mitochondrial deafness. *Ann Med* 29: 483-91. 20. Fischel-Ghodsian N, Prezant TR, Fournier P et al (1995) Mitochondrial mutation associated with nonsyndromic deafness. *Am J Otolaryngol* 16:403-8. 21. Yanagida C, Ito K, Komiya I et al (2004) Protective effect of fosfomycin on gentamicin-induced lipid peroxidation of rat renal tissue. *Chem Biol Interact* 148: 139-47. 22. Quiros Y, Vicente-Vicente L, Morales AI et al (2011) An integrative overview on the mechanisms underlying the renal tubular cytotoxicity of gentamicin. *Toxicol Sci* 119: 245-56. 23. Chiu PY, Leung HY, Siu AH et al (2008) Long-term treatment with a Yang-invigorating Chinese herbal formula produces generalized tissue protection against oxidative damage in rats. *Rejuvenation Res* 11: 43-62. 24. Matt T, Ng CL, Lang K et al (2012) Dissociation of antibacterial activity and aminoglycoside ototoxicity in the 4-monosubstituted 2-deoxystreptamine apramycin. *Proc Natl Acad Sci USA* 109: 10984-9. 25. Rydzanicz M, Cywińska K, Wróbel M et al (2011) The contribution of the mitochondrial COI/tRNA(Ser(UCN)) gene mutations to non-syndromic and aminoglycoside-induced hearing loss in Polish patients. *Mol Genet Metab* 104: 153-9. 26. Zhao H, Young WY, Yan Q et al (2005) Functional characterization of the mitochondrial 12S rRNA C1494T mutation associated with aminoglycoside-induced and non-syndromic hearing loss. *Nucleic Acids Res* 33: 1132-9. 27. Hobbie SN, Akshay S, Kalapala SK et al (2008) Genetic analysis of interactions with eukaryotic rRNA identify the mitoribosome as target in aminoglycoside ototoxicity. *Proc Natl Acad Sci USA* 105: 20888-93. 28. Quan Y, Xia L, Shao J et al (2015) Adjudin protects rodent cochlear hair cells against gentamicin ototoxicity via the SIRT3-ROS pathway. *Sci Rep* 5: 8181. 29. Ng MR, Antonelli PJ, Joseph J et al (2015) Assessment of mitochondrial membrane potential in HEI-OC1 and LLC-PK1 cells treated with gentamicin and mitoquinone. *Otolaryngol Head Neck Surg* 152: 729-33. 30. Rustenbeck I, Eggers G, Reiter H et al (1998) Polyamine modulation of mitochondrial calcium transport I Stimulatory and inhibitory effects of aliphatic polyamines, aminoglucosides and other polyamine analogues on mitochondrial calcium uptake. *Biochem Pharmacol* 56: 977-85. 31. Kohn S, Fradis M, Ben-David J et al (2002) Nephrotoxicity of combined treatment with cisplatin and gentamicin in the guinea pig: glomerular injury findings. *Ultrastruct Pathol* 26: 371-82. 32. Lingala HB, Penagaluru PR (2009) Role of connexin 26 (GJB2) &amp; mitochondrial small ribosomal RNA (mt 12S rRNA) genes in sporadic &amp; aminoglycoside-induced non syndromic hearing impairment. *Indian J Med Res* 130: 369-78. 33. Zorov DB (2010) Amelioration of aminoglycoside nephrotoxicity requires protection of renal mitochondria. *Kidney Int* 77: 841-3. 34. Tagliati CA, Romero D, Dutra EC et al (2012) A comparison of BGM and LLC-PK1 cells for the evaluation of nephrotoxicity. *Drug Chem Toxicol* 35: 258-63. 35. Adeyemo AA, Oluwatosin O, Omotade OO (2016) Study of streptomycin-induced ototoxicity: protocol for a longitudinal study. *Springerplus* 5: 758. 36. Félix L, Oliveira MM, Videira R et al (2017) Carvedilol exacerbate gentamicin-induced kidney mitochondrial alterations in adult rat. *Exp Toxicol Pathol* 69 :83-92. 37. Notenboom S, Miller DS, Kuik LH et al (2005) Short-term exposure of renal proximal tubules to gentamicin increases long-term multidrug resistance protein 2 (Abcc2) transport function and reduces nephrotoxicant sensitivity. *J Pharmacol Exp Ther* 315: 912-20. 38. Zhu X, He X, Wang W et al (2015) MTO1 worked as a modifier in the aminoglycosides sensitivity of yeast carrying a mitochondrial 15S rRNA C1477G mutation. *PLoS One* 10: e0124200. 39. He Z, Sun S, Waqas M et al (2016) Reduced TRMU expression increases the sensitivity of hair-cell-like HEI-OC-1 cells to neomycin damage in vitro. *Sci Rep* 6: 29621. 40. Walker PD, Shah SV (1987) Gentamicin enhanced production of hydrogen peroxide by renal cortical mitochondria. *Am J Physiol* 253: C495-9. 41. Shah SV, Walker PD (1992) Reactive oxygen metabolites in toxic acute renal failure. *Ren Fail* 14: 363-70. 42. Chiu PY, Leung HY, Ko KM (2008) Schisandrin B Enhances Renal Mitochondrial Antioxidant Status, Functional and Structural Integrity, and Protects against Gentamicin-Induced Nephrotoxicity in Rats. *Biol Pharm Bull* 31: 602-5. 43. Jensen-Smith HC, Hallworth R, Nichols MG (2012) Gentamicin rapidly inhibits mitochondrial metabolism in high-frequency cochlear outer hair cells. *PLoS One* 7: e38471. 44. Ojano-Dirain CP, Antonelli PJ, Le Prell CG (2014) Mitochondria-targeted antioxidant MitoQ reduces gentamicin-induced ototoxicity. *Otol Neurotol* 35: 533-9. 45. Choi J, Chang J, Jun HJ et al (2014) Protective role of edaravone against neomycin-induced ototoxicity in zebrafish. *J Appl Toxicol* 34: 554-61. 46. Cui J, Bai XY, Sun X et al (2015) Rapamycin protects against gentamicin-induced acute kidney injury via autophagy in mini-pig models. *Sci Rep* 5: 11256. 47. Kim YR, Kim MA, Cho HJ et al (2016) Galangin prevents aminoglycoside-induced ototoxicity by decreasing mitochondrial production of reactive oxygen species in mouse cochlear cultures. *Toxicol Lett* 245: 78-85. 48. Abuelezz SA, Hendawy N, Abdel Gawad S (2016) Alleviation of renal mitochondrial dysfunction and apoptosis underlies the protective effect of sitagliptin in gentamicin-induced nephrotoxicity. *J Pharm Pharmacol* 68: 523-32. 49. Yu X, Fan Z, Han Y et al (2018) Paeoniflorin reduces neomycin-induced ototoxicity in hair cells by suppression of reactive oxygen species generation and extracellularly regulated kinase signalization. *Toxicol Lett* 285: 9-19. 50. Adil M, Kandhare AD, Dalvi G et al (2016) Ameliorative effect of berberine against gentamicin-induced nephrotoxicity in rats via attenuation of oxidative stress, inflammation, apoptosis and mitochondrial dysfunction. *Ren Fail* 38: 996-1006. 51. Charlwood J, Skehel JM, King N et al (2002) Proteomic analysis of rat kidney cortex following treatment with gentamicin. *J Proteome Res* 1: 73-82. 52. Kifer-Wysocka E, Romanowska-Sarlej J, Karwan A et al (2004) The proximal convoluted tubule of rats' nephron after experimental administration of gentamicin. *Ann Univ Mariae Curie Sklodowska Med* 59: 86-90. 53. Servais H, Ortiz A, Devuyst O et al (2008) Renal cell apoptosis induced by nephrotoxic drugs: cellular and molecular mechanisms and potential approaches to modulation. *Apoptosis* 13: 11-32. 54. Trujillo J, Chirino YI, Molina-Jijón E et al (2013) Renoprotective effect of the antioxidant curcumin: Recent findings. *Redox Biol* 1: 448-56. 55. Wong HS, Chen JH, Leong PK et al (2014) β-sitosterol protects against carbon tetrachloride hepatotoxicity but not gentamicin nephrotoxicity in rats via the induction of mitochondrial glutathione redox cycling. *Molecules* 19: 17649-62. 56. Song Y, Fan Z, Bai X et al (2016) PARP-1-modulated AIF translocation is involved in streptomycin-induced cochlear hair cell death. *Acta Otolaryngol* 136: 545-50. 57. Zhou S, Sun Y, Kuang X et al (2018) Mitochondria-homing peptide functionalized nanoparticles performing dual extracellular/intracellular roles to inhibit aminoglycosides induced ototoxicity. *Artif Cells Nanomed Biotechnol* 46: 314-323. 58. Oda Y, Harada Y, Kasuga S et al (1995) Effect of streptomycin on the supporting cells of the utricular macula. *Acta Otolaryngol Suppl* 519: 238-43. 59. Beeson CC, Beeson GC, Schnellmann RG (2010) A high-throughput respirometric assay for mitochondrial biogenesis and toxicity. *Anal Biochem* 404: 75-81. 60. Chang J, Jung HH, Yang JY et al (2011) Protective role of antidiabetic drug metformin against gentamicin induced apoptosis in auditory cell line. *Hear Res* 282: 92-6. 61. Jadidian A, Antonelli PJ, Ojano-Dirain CP (2015) Evaluation of apoptotic markers in HEI-OC1 cells treated with gentamicin with and without the mitochondria-targeted antioxidant mitoquinone. *Otol Neurotol* 36: 526-30. 62. Setz C, Benischke AS, Pinho Ferreira Bento AC et al (2018) Induction of mitophagy in the HEI-OC1 auditory cell line and activation of the Atg12/LC3 pathway in the organ of Corti. *Hear Res* 361: 52-65. |

| **Amiodarone** |
| --- |
| **Mode of action** |
| Antiarrhythmic drug. Increases the duration of ventricular and atrial muscle action by inhibiting Na,K-activated myocardial adenosine triphosphatase. There is a resulting decrease in heart rate and in vascular resistance. The antiarrhythmic effect of amiodarone may be due to at least two major actions. It prolongs the myocardial cell-action potential (phase 3) duration and refractory period and acts as a noncompetitive a- and b-adrenergic inhibitor. Amiodarone is eliminated primarily by hepatic metabolism (CYP2C8) and biliary excretion and there is negligible excretion of amiodarone or DEA in urine. The most serious adverse effects are neuropathy, pulmonary fibrosis, microvesicular steatosis (in severe forms leading to liver failure) or thyroid dysfunction. 10-15% of patients has to stop using the drug. |
| **Theoretical effects on mitochondrial function** |
| The inhibition of adrenergic receptors could lead to decrease in Ca influx, which may affect the mitochondrial function. |
| **Known effects on mitochondrial function** |
| Amiodarone enters the mitochondria in the protonated form along the membrane potential and is accumulated by mitochondria where the pH is relatively alkaline, releasing protons that bypass the ATPase, thereby uncoupling mitochondrial ATP synthesis by oxidative phosphorylation.  Increase of mitochondrial ROS production and lipid peroxidation.  Inhibition of complex I-III. Dose dependent.  Inhibition of fatty acid beta-oxidation. Dose dependent.  Inhibition of the TCA cycle.  Inhibition of CPT1 and acyl-CoA dehydrogenases, thus inhibition of beta-oxidation. Studies in rat liver.  Cardiomyocytes are more resistant to amiodarone, do not induce ROS formation even in high doses.  Modifies the phospholipid composition of cellular compartments and cell membranes. |
| **Effects measured/seen in mitochondrial patients, mitochondrial cell lines or normal cell lines** |
| - Microfluidic liver-on-chip devices. HepG2/C3Acells. Oxygen consumption. Phosphorescence system. Identify subthreshold toxicity. Oxygen uptake stayed the same after one dosage, but dropped during 24 hours exposure. The higher the dosage the lesser the % of normal respiration. And the higher the dosage the earlier the respiratory damage occurred. Suggesting that the accumulation of the toxic intermediate is responsible, rather than direct damage by the parent compound. - In vivo mouse model. Hepatic ATP levels did not change. Cytosolic cytochrome c was increased suggesting apoptosis. One dosage administration. - In vivo mouse model. Repeated oral treatment, 6 days. Also model with cotreatment with antioxidant N-acetylcysteine (NAC). Amiodarone induces mitochondrial oxidative stress (measurement of GSH , GSSG, H2O2 production). Effect on membrane permeability of mitochondria. Uncoupling effect. Impair complex I activity, due to increase in cardiolipin oxidation. ATP content was lower. Normal complex V activity. Normalization in animals treated with NAC: complex I, GSH, lipid peroxidation, membrane potential, hepatic ATP content. - Isolated rat liver mitochondria, rat liver cells, HEPg2 cells. Uncoupling properties of amiodarone. Decreased palmitate oxidation, dose-dependent manner. Increased ROS production. - Rat hearts, ischemia-reperfusion condition. Cardiomyocytes and hepatocytes to study effect of amiodarone in cardiac and extra-cardiac tissue. Oxygen consumption in low concentrations was comparable to control. In intermediate concentration range increase in oxygen consumption, and higher concentrations showed inhibition. Same accounts for mitochondrial permeability. Both in heart and liver. No induction of ROS production. Cell viability differs between tissues. - Isolated mitochondria. Biphasic effect on state 4 respiration in liver mitochondria. Its initial stimulatory effect on state 4 respiration is probably related to the entry of protonated amiodarone along the mitochondrial membrane potential, followed by its dissociation in the matrix and the release of a proton and of uncharged amiodarone. Progressive accumulation of amiodarone in the mitochondrion soon leads to inhibition of the respiratory chain; amiodarone selectively affects the transfer of electrons to, in or from, both complex I and complex II. This decreases both state 4 and state 3 respiration and the generation of ATP. |
| **References** |
| [Chan K](https://www.ncbi.nlm.nih.gov/pubmed/?term=Chan%20K%5BAuthor%5D&cauthor=true&cauthor_uid=16863431), [Truong D](https://www.ncbi.nlm.nih.gov/pubmed/?term=Truong%20D%5BAuthor%5D&cauthor=true&cauthor_uid=16863431), [Shangari N](https://www.ncbi.nlm.nih.gov/pubmed/?term=Shangari%20N%5BAuthor%5D&cauthor=true&cauthor_uid=16863431), et al. Drug-induced mitochondrial toxicity. [*Expert Opin Drug Metab Toxicol.*](https://www.ncbi.nlm.nih.gov/pubmed/16863431) 21:655-69.  [Fromenty B](https://www.ncbi.nlm.nih.gov/pubmed/?term=Fromenty%20B%5BAuthor%5D&cauthor=true&cauthor_uid=1979817), [Fisch C](https://www.ncbi.nlm.nih.gov/pubmed/?term=Fisch%20C%5BAuthor%5D&cauthor=true&cauthor_uid=1979817), [Berson A](https://www.ncbi.nlm.nih.gov/pubmed/?term=Berson%20A%5BAuthor%5D&cauthor=true&cauthor_uid=1979817), et al (1990) Dual effect of amiodarone on mitochondrial respiration. Initial protonophoric uncoupling effect followed by inhibition of the respiratory chain at the levels of complex I and complex II. [*J Pharmacol Exp Ther*.](https://www.ncbi.nlm.nih.gov/pubmed/1979817) 255: 1377-84.  [Prill S](https://www.ncbi.nlm.nih.gov/pubmed/?term=Prill%20S%5BAuthor%5D&cauthor=true&cauthor_uid=26041127), [Bavli D](https://www.ncbi.nlm.nih.gov/pubmed/?term=Bavli%20D%5BAuthor%5D&cauthor=true&cauthor_uid=26041127), [Levy G](https://www.ncbi.nlm.nih.gov/pubmed/?term=Levy%20G%5BAuthor%5D&cauthor=true&cauthor_uid=26041127), et al (2016) Real-time monitoring of oxygen uptake in hepatic bioreactor shows CYP450-independent mitochondrial toxicity of acetaminophen and amiodarone. [*Arch Toxicol.*](https://www.ncbi.nlm.nih.gov/pubmed/26041127) 90: 1181-91.  [Serviddio G](https://www.ncbi.nlm.nih.gov/pubmed/?term=Serviddio%20G%5BAuthor%5D&cauthor=true&cauthor_uid=21971348), [Bellanti F](https://www.ncbi.nlm.nih.gov/pubmed/?term=Bellanti%20F%5BAuthor%5D&cauthor=true&cauthor_uid=21971348), [Giudetti AM](https://www.ncbi.nlm.nih.gov/pubmed/?term=Giudetti%20AM%5BAuthor%5D&cauthor=true&cauthor_uid=21971348), et al (2011) Mitochondrial oxidative stress and respiratory chain dysfunction account for liver toxicity during amiodarone but not dronedarone administration*.* [*Free Radic Biol Med.*](https://www.ncbi.nlm.nih.gov/pubmed/21971348) 51: 2234-42.  [Takai S](https://www.ncbi.nlm.nih.gov/pubmed/?term=Takai%20S%5BAuthor%5D&cauthor=true&cauthor_uid=25900201), [Oda S](https://www.ncbi.nlm.nih.gov/pubmed/?term=Oda%20S%5BAuthor%5D&cauthor=true&cauthor_uid=25900201), [Tsuneyama K](https://www.ncbi.nlm.nih.gov/pubmed/?term=Tsuneyama%20K%5BAuthor%5D&cauthor=true&cauthor_uid=25900201), et al (2016) Establishment of a mouse model for amiodarone-induced liver injury and analyses of its hepatotoxic mechanism. [*J Appl Toxic*.](https://www.ncbi.nlm.nih.gov/pubmed/?term=takai+amiodarone-induced)6: 35-47.  [Varbiro G](https://www.ncbi.nlm.nih.gov/pubmed/?term=Varbiro%20G%5BAuthor%5D&cauthor=true&cauthor_uid=12663047), [Toth A](https://www.ncbi.nlm.nih.gov/pubmed/?term=Toth%20A%5BAuthor%5D&cauthor=true&cauthor_uid=12663047), [Tapodi A](https://www.ncbi.nlm.nih.gov/pubmed/?term=Tapodi%20A%5BAuthor%5D&cauthor=true&cauthor_uid=12663047), et al (2003) Concentration dependent mitochondrial effect of amiodarone. [*Biochem Pharmacol.*](https://www.ncbi.nlm.nih.gov/pubmed/12663047) 65: 1115-28.  [Waldhauser KM](https://www.ncbi.nlm.nih.gov/pubmed/?term=Waldhauser%20KM%5BAuthor%5D&cauthor=true&cauthor_uid=16971508), [Török M](https://www.ncbi.nlm.nih.gov/pubmed/?term=T%C3%B6r%C3%B6k%20M%5BAuthor%5D&cauthor=true&cauthor_uid=16971508), [Ha HR](https://www.ncbi.nlm.nih.gov/pubmed/?term=Ha%20HR%5BAuthor%5D&cauthor=true&cauthor_uid=16971508), et al (2006) Hepatocellular toxicity and pharmacological effect of amiodarone and amiodarone derivatives. [*J Pharmacol Exp Ther*.](https://www.ncbi.nlm.nih.gov/pubmed/16971508) 319: 1413-23. |

| **Articaine** |
| --- |
| **Mode of action** |
| It is an amide-type local anesthetic that blocks nerve conduction by reversibly binding to the α-subunit of the voltage-gated sodium channels within the inner cavity of the nerve, similar to other local anesthetics. Binding of articaine to the sodium channel reduces sodium influx so that the threshold potential will not be reached and impulse conduction stops. Articaine is unique among the amides because it is based on a thiophene ring structure. |
| **Theoretical effects on mitochondrial function** |
| Articaine inhibits mitochondria bioenergetics and block respiratory chain complex I. There are concentration-dependent neurotoxic injuries after injection of articaine in an electrophysiological and stereological study of the rat sciatic nerve with a significant difference between 2% and 4% formulations – not mitochondrial studies have been performed. |
| **Known effects on mitochondrial function** |
| Have not been studied |
| **Effects measured/seen in mitochondrial patients, mitochondrial cell lines or normal cell lines** |
| There is one report on the literature of adverse effects in a Kearns-Sayre patient. Five minutes after the injection of articaine, the patient developed weakness of the limb muscles, extreme fatigue with increased desire to sleep, a feeling of heat and frequent urination. The adverse reaction resolved spontaneously within 48 hours without sequelae.  No study exists on mitochondrial cell lines. |
| **References** |
| Finsterer J, Haberler C, Schmiedel J (2005) Deterioration of Kearns-Sayre syndrome following articaine administration for local anesthesia. *Clin Neuropharmacol* 28(3): 148-9.  Gunaydin B, Demiryurek AT(2001) Effects of prilocaine and articaine on human leucocytes and reactive oxygen species in vitro. *Acta Anaesthesiologica Scandinavica* 45: 741–745.  Nouette-Gaulain K, Jose C, Capdevila X et al (2011) From analgesia to myopathy: When local anesthetics impair the mitochondrion. *Int J Biochem Cell Biol* 43: 14-9. |

| **Barbiturates** |
| --- |
| **Mode of action** |
| The barbiturates bind to molecular components of the GABA-A receptor in neuronal membranes in the central nervous system. This receptor, which functions as a chloride ion channel, is activated by the inhibitory neurotransmitter GABA. Barbiturates also facilitate the actions of GABA at multiple sites in the central nervous system and they appear to increase the duration of the GABA-gated chloride channel openings. At high concentrations, the barbiturates may also be GABA-mimetic, directly activating chloride channels. They also depress the actions of the excitatory neurotransmitter glutamic acid via binding to the AMPA receptor. |
| **Theoretical effects on mitochondrial function** |
| Barbiturates can directly depress mitochondrial respiration interfering with ATP synthesis and electron transport. They can also potentiate NMDA-induced neurotoxicity. |
| **Known effects on mitochondrial function** |
| Barbiturates are the first drugs found to inhibit mitochondrial respiration by inhibiting the NADH dehydrogenase. Studies have suggested that mitochondria are an intracellular site of barbiturates action. Cell-permeant barbiturates cause significant depolarization with consequent reduction in ATP synthesis, probably due to impaired electron transport or enhanced inner membrane proton permeability. Moreover, NMDA alone causes a small mitochondrial depolarization, which is increased by the presence of barbiturates (Anderson, 2002). Further studies showed that phenobarbital is able to inhibit state-3 and state-4 respiration, decrease ATP synthesis and alter Ca ^2+^ homeostasis inhibiting mitochondrial swelling (Santos, 2008). Nevertheless, some studies underlined a possible protective effect on mitochondria of some barbiturates, slightly protecting oxidative phosphorylation and increasing ATP production (Berger,2010). |
| **Effects measured/seen in mitochondrial patients, mitochondrial cell lines or normal cell lines** |
| Barbiturates exert their effect on mitochondria interfering with various mitochondrial functions such as oxidative phosphorylation, channels, membrane-bound receptors and membrane potential and anti-oxidative defence, without depletion of mitochondrial DNA. Some studies also showed that phenobarbital produces an increase in microsomal and mitochondrial proteins without a corresponding increase in mitochondrial number, this can be explained with morphological and ultrastructural changes in mitochondria (‘’dumbbell’’, U- and O- shaped) (Karbowski, 1997; Almsheqi, 2007).  In vitro studies demonstrated that non-bioactivated phenobarbital (PB) is able to inhibit state-3 respiration, decrease ATP synthesis and mitochondrial membrane potential. Parent PB did not affect state-4 respiration, Ca^2+^ homeostasis and mitochondrial swelling, suggesting an intact internal mitochondrial membrane. Only after bio-activation PB was able to impair Ca^2+^ transport and inhibit mitochondrial swelling probably because of the inability of the mitochondria to maintain the required Ca^2+^ concentration to trigger the opening of the permeability transition pore and induce mitochondrial swelling (Santos, 2008). It has been suggested that AAEDs’ (aromatic anti-epileptic drugs) hepatotoxicity could be linked to their effects on mitochondria and consequent mitochondrial dysfunction. Regarding this class of drugs, phenobarbital’s mitochondrial toxicity resulted greater than carbamazepine, but less than phenytoin.  However, phenobarbital showed a mild stimulating effect on ATP production in a study conducted on five children and afforded protection against 3-nitropropionic acid (a mitochondrial toxin) induced seizures in mice (Berger, 2010).  Regarding thiopental an in vitro study, conducted on myocytes exposed to several anesthetic agents and dioxide (a known opener of mitoK_ATP_ channels), showed a significant inhibitory effect of these anesthetics (included Thiopental) on mitoK_ATP_ channel activity (Zaugg, 2002). Another in vitro study conducted on thiopental showed an increased generation of mitochondrial ROS among T cells incubated with thiopental but no differences in the GSH mitochondrial level of the thiopental-treated lymphocytes and the control group lymphocytes (Delogu, 2004). Some studies (Nishina, 1998; Almaas, 2000; Harman, 2012) reported thiopental having an antioxidant effect (reducing lipidic peroxidation and ROS releasing) but without enough preventing effect on mitochondrial damage in ischemia-reperfusion models (Harman, 2012). In clinical practice thiopental has been used as induction agent successfully and with little negative effect in mitochondrial patients when used as a bolus (Niezgoda, 2013) and a review of anesthetic outcomes in patients with genetically confirmed mitochondrial disorders reported one case where thiopental has been used as induction without perioperative complications (Smith, 2016). |
| **References** |
| Aldakkak M, Stowe DF, Chen Q et al (2008) Inhibited mitochondrial respiration by amobarbital during cardiac ischaemia improves redox state and reduces matrix Ca2+ overload and ROS release. *Cardiovasc Res* 77: 406-15.  Almsherqi Z, McLachlan CS, Tay SK et al (2007) Chronic phenobarbital-induced mitochondrial pleomorphism in the rat liver. *Toxicol Path* 35: 833-5.  Anderson CM, Norquist BA, Vesce S et al (2002) Barbiturates induce mitochondrial depolarization and potentiate excitotoxic neuronal death. *J Neurosci* 22: 9203-9.  Berger I, Segal I, Shmueli D et al (2010) The effect of antiepileptic drugs on mitochondrial activity: a pilot study. *J Child Neurol* 25: 541-5.  Chen Q, Hoppel CL, Lesnefsky EJ (2006) Blockade of electron transport before cardiac ischemia with the reversible inhibitor amobarbital protects rat heart mitochondria. *J Pharmacol Exp Ther* 316: 200-7.  Delogu G, Antonucci A, Moretti S et al (2004) Oxidative stress and mitochondrial glutathione in human lymphocytes exposed to clinically relevant anesthetic drug concentrations. *J Clin Anesth* 16: 189-94.  Harman F, Hasturk AE, Yaman M et al (2012) Neuroprotective effects of propofol, thiopental, etomidate, and midazolam in fetal rat brain in ischemia-reperfusion model. *J Neurosurgery Pediatr* 28: 1055-62.  Karbowski M, Kurono C, Nishizawa Y et al (1997) Induction of megamitochondria by some chemicals inducing oxidative stress in primary cultured rat hepatocytes. *Biochim Biophys Acta* 1349: 242-50.  Keel M, Mica L, Stover J et al (2005) Thiopental-induced apoptosis in lymphocytes is independent of CD95 activation. *Anesthesiol* 103: 576-84.  Komissarova IA, Nartsissov YR, Burbenskaya NM. Effects of phenobarbital on activity of mitochondrial enzymes in peripheral blood lymphocytes and oxidative phosphorylation in liver mitochondria. *Bull Exp Biol Med* 131: 330-2.  Nishina K, Akamatsu H, Mikawa K et al (1998) The inhibitory effects of thiopental, midazolam, and ketamine on human neutrophil functions. *Anesth Analges* 86: 159-65.  Santos NA, Medina WS, Martins NM et al (2008) Aromatic antiepileptic drugs and mitochondrial toxicity: effects on mitochondria isolated from rat liver. *Toxicol In Vitro* 22: 1143-52.  Santos NA, Medina WS, Martins NM et al (2008) Involvement of oxidative stress in the hepatotoxicity induced by aromatic antiepileptic drugs. *Toxicol In Vitro* 22:1820-4.  Sonnay S, Duarte JMN, Just N et al (2017) Energy metabolism in the rat cortex under thiopental anaesthesia measured In Vivo by (13) C MRS. *J Neurosci Res* 95: 2297-306.  Urbanska EM, Blaszczak P, Saran T et al (1998) Mitochondrial toxin 3-nitropropionic acid evokes seizures in mice. *Eur J Pharmacol* 359: 55-8.  Zaugg M, Lucchinetti E, Spahn DR et al (2002) Differential effects of anesthetics on mitochondrial K(ATP) channel activity and cardiomyocyte protection. *Anesthesiol* 97: 15-23. |

| **Beta blockers** |
| --- |
| **Mode of action** |
| Beta blockers are a class of medications that are used to manage cardiac arrhythmias, acute myocardial infarction, mild to moderate hypertension and prophylaxis of migraine. Beta blockers are [competitive antagonists](https://en.wikipedia.org/wiki/Competitive_antagonist) that block the receptor sites for the [endogenous](https://en.wikipedia.org/wiki/Endogenous) [catecholamines](https://en.wikipedia.org/wiki/Catecholamine) [epinephrine](https://en.wikipedia.org/wiki/Adrenaline) (adrenaline) and [norepinephrine](https://en.wikipedia.org/wiki/Norepinephrine) (noradrenaline) on [adrenergic beta receptors](https://en.wikipedia.org/wiki/Beta_receptor), of the [sympathetic nervous system](https://en.wikipedia.org/wiki/Sympathetic_nervous_system). Some block activation of all types of [β-adrenergic receptors](https://en.wikipedia.org/wiki/%CE%92-adrenergic_receptor) and others are selective for one of the three known types of beta receptors, designated β_1_, β_2_ and β_3_ receptors. [β_1_-adrenergic receptors](https://en.wikipedia.org/wiki/Beta-1_adrenergic_receptor) are located mainly in the heart and in the kidneys. [β_2_-adrenergic receptors](https://en.wikipedia.org/wiki/Beta-2_adrenergic_receptor) are located mainly in the lungs, gastrointestinal tract, liver, uterus, vascular smooth muscle, and skeletal muscle. [β_3_-adrenergic receptors](https://en.wikipedia.org/wiki/Beta-3_adrenergic_receptor) are located in fat cells.  Metoprolol is a cardioselective β1-adrenergic blocking agent. Primarily hepatic metabolized. Less than 5% of an oral dose of metoprolol is recovered unchanged in the urine; the rest is excreted by the kidneys as metabolites that appear to have no beta-blocking activity.  Carvedilol is a non-selective beta, it blocks beta-1 and beta-2 adrenergic receptors as well as the alpha-1 adrenergic receptors. Carvedilol and its metabolite BM-910228 (a less potent beta blocker, but more potent antioxidant) have been shown to restore the inotropic responsiveness to Ca^2+^ in OH^-^ free radical-treated myocardium. Carvedilol and its metabolites also prevent OH^-^ radical-induced decrease in sarcoplasmic reticulum Ca^2+^-ATPase activity. Therefore, carvedilol and its metabolites may be beneficial in chronic heart failure by preventing free radical damage. NADH dehydrogenase (ubiquinone) 1 subunit C2 is a target of carvedilol, inhibition of the enzyme. Hepatic metabolized. Carvedilol is extensively metabolized. Less than 2% of the dose was excreted unchanged in the urine. Carvedilol is metabolized primarily by aromatic ring oxidation and glucuronidation. The oxidative metabolites are further metabolized by conjugation via glucuronidation and sulfation. The metabolites of carvedilol are excreted primarily via the bile into the feces.  Propranolol, the prototype of the beta-adrenergic receptor antagonists, is a competitive, nonselective beta-blocker without intrinsic sympathomimetic activity. Hepatic metabolized. Propranolol is extensively metabolized with most metabolites appearing in the urine. |
| **Theoretical effects on mitochondrial function** |
| The inhibition of adrenergic receptors could lead to decrease in Ca influx, which may affect the mitochondrial function. Direct effect of Carvedilol on exogenous NADH dehydrogenase may negatively affect mitochondrial function. |
| **Known effects on mitochondrial function** |
| Inhibition of ATPase and stage 3 respiration  Inhibition of complex I  Carvedilol has been shown to prevent, in isolated heart mitochondria, the calcium-induced mitochondrial permeability transition (MPT) occurring in the post-ischemic reperfusion of the heart by avoiding protein thiol oxidation. On the other hand, carvedilol can exert pro-oxidant effects. In fact, it has been reported that this drug can induce, in isolated mitochondria, a marked inhibition of mitochondrial respiration by NAD-dependent substrates and a specific inhibition of mitochondrial complex I activity, with these effects being associated with an increased production of ROS.  The signaling to the mitochondria from the adrenergic receptor pathway has not yet been well elucidated. The cytosolic Ca2+ most likely plays an important role in mediating the adrenergic receptor signals to the mitochondria in order to regulate the ATP production. As a result, carvedilol might act to inhibit the Ca2+ entry into the mitochondria. Carvedilol can also inhibit the augmented mV• O2 caused by Ca2+ overload through an inhibitory effect on the respiratory uncoupling. In addition, the protonophoretic characteristics of carvedilol might be involved in this mechanism.  In a state of heart failure, complex I has been shown to be a major source of ROS in the mitochondria, and this rotenone specific domain has also been shown to be sensitive to Ca2+ in the production of ROS. As a result, carvedilol might act on this rotenone-sensitive domain of complex I in order to inhibit the ROS production caused by Ca2+ overload. |
| **Effects measured/seen in mitochondrial patients, mitochondrial cell lines or normal cell lines** |
| - H9C2 cells. The protection of rat heart H9C2 cells from H2O2-induced damage by a double action of carvedilol, i.e. a direct antioxidant effect and a preconditioning-like mechanism via mitochondrial complex I inhibition. - 52-year-old woman developed double vision, myalgias, muscle cramps, and hip and thigh muscle stiffness. Clinical neurologic examination revealed ptosis, dysarthria, sore neck muscles, weakness and wasting of the thighs, and generally brisk tendon reflexes. Lactate stress testing was significantly abnormal. Needle electromyography was nonspecifically abnormal and myopathic. Muscle biopsy showed mild myopathic changes, target fibers, and a single COX-negative fiber. Probable mitochondrial disorder was diagnosed. 30 mg of propranolol during 7 years for arterial hypertension. Shortly after discontinuation of the drug, her double vision gradually disappeared, myalgias and muscle cramps gradually resolved, and the patient reported an increase in muscle mass on repeated follow-ups. Long-term administration of propranolol may aggravate a mitochondrial disorder. Discontinuation of propranolol may result in a gradual resolution of these adverse reactions. - In Chang conjunctival cells timolol decreased cellular viability and induced chromatin condensation which decreased mitochondrial membrane potential. - A pediatric patient with a mitochondrial disorder (genetic defect not mentioned) who developed respiratory distress after metoprolol was prescribed for hypertension. As he improved with discontinuation of medication and no alternative etiology was found for the symptoms, it was surmised that administration of metoprolol aggravated the mitochondrial dysfunction, thus worsening underlying chest wall weakness. - Isolated rat heart mitochondria. Mitochondria mimicking a state of heart failure by impairing complex I with rotenone (100 mol/L) became sensitive to Ca2+ overload in order to increase the ROS production. The effect of carvedilol on the mitochondrial function, such as oxygen consumption and ROS production, is distinct from that of metoprolol. Carvedilol differs significantly from metoprolol in inhibiting the Ca2+ overload-induced mitochondrial oxygen consumption and the ROS production during an impairment of complex I. - HUVECs endothelial cells. Carvedilol stimulates the PGC-1alpha-NRF1-TFAM pathway. It promotes mitochondrial biogenesis (mtDNA content, cytochome C protein, COX protein. It improves mitochondrial respiration. - Isolated rat heart mitochondria. Carvedilol is a specific inhibitor of the exogenous NADH dehydrogenase. |
| **References** |
| Cocco T, Cutecchia G, Montedoro G et al (2002) The antihypertensive drug carvedilol inhibits the activity of mitochondrial NADH-ubiquinone oxidoreductase. J Bioenerg Biomembr 34: 251–8.  Debbasch C, Pisella PJ, De Saint Jean M et al (2001) Mitochondrial activity and glutathione injury in apoptosis induced by unpreserved and preserved beta-blockers on Chang conjunctival cells. *Invest Ophthalmol Vis Sci* 42: 2525-33.  Di Lisa F, Menabo, R, Canton M et al (2001) Opening of the mitochondrial permeability transition pore causes depletion of mitochondrial and cytosolic NAD+ and is a causative event in the death of myocytes in postischemic reperfusion of the heart. *J Biol Chem* 276: 2571–5.  [Kametani R](https://www.ncbi.nlm.nih.gov/pubmed/?term=Kametani%20R%5BAuthor%5D&cauthor=true&cauthor_uid=16501300), [Miura T](https://www.ncbi.nlm.nih.gov/pubmed/?term=Miura%20T%5BAuthor%5D&cauthor=true&cauthor_uid=16501300), [Harada N](https://www.ncbi.nlm.nih.gov/pubmed/?term=Harada%20N%5BAuthor%5D&cauthor=true&cauthor_uid=16501300) et al (2006) Carvedilol inhibits mitochondrial oxygen consumption and superoxide production during calcium overload in isolated heart mitochondria *Circ J* 70: 321-6.  [Oliveira PJ](https://www.ncbi.nlm.nih.gov/pubmed/?term=Oliveira%20PJ%5BAuthor%5D&cauthor=true&cauthor_uid=10666308), [Santos DJ](https://www.ncbi.nlm.nih.gov/pubmed/?term=Santos%20DJ%5BAuthor%5D&cauthor=true&cauthor_uid=10666308), [Moreno AJ](https://www.ncbi.nlm.nih.gov/pubmed/?term=Moreno%20AJ%5BAuthor%5D&cauthor=true&cauthor_uid=10666308) (2000) Carvedilol inhibits the exogenous NADH dehydrogenase in rat heart mitochondria. [*Arch Biochem Biophys.*](https://www.ncbi.nlm.nih.gov/pubmed/10666308) 374: 279-85.  Oliveira PJ, Goncalves L, Monteiro P et al (2005) Are the antioxidant properties of carvedilol important for the protection of cardiac mitochondria? *Curr Vasc Pharmacol* 3: 147–58.  [Samuels C](https://www.ncbi.nlm.nih.gov/pubmed/?term=Samuels%20C%5BAuthor%5D&cauthor=true&cauthor_uid=27840760), [Koenig MK](https://www.ncbi.nlm.nih.gov/pubmed/?term=Koenig%20MK%5BAuthor%5D&cauthor=true&cauthor_uid=27840760), [Hernandez M](https://www.ncbi.nlm.nih.gov/pubmed/?term=Hernandez%20M%5BAuthor%5D&cauthor=true&cauthor_uid=27840760) [et](https://www.ncbi.nlm.nih.gov/pubmed/?term=Yadav%20A%5BAuthor%5D&cauthor=true&cauthor_uid=27840760) al (2016) Mitochondrial Disorder Aggravated by Metoprolol. [*Case Rep Pediatr.*](https://www.ncbi.nlm.nih.gov/pubmed/?term=samuels+metoprolol)  [Sgobbo P](https://www.ncbi.nlm.nih.gov/pubmed/?term=Sgobbo%20P%5BAuthor%5D&cauthor=true&cauthor_uid=17346667), [Pacelli C](https://www.ncbi.nlm.nih.gov/pubmed/?term=Pacelli%20C%5BAuthor%5D&cauthor=true&cauthor_uid=17346667), [Grattagliano I](https://www.ncbi.nlm.nih.gov/pubmed/?term=Grattagliano%20I%5BAuthor%5D&cauthor=true&cauthor_uid=17346667) et al (2007) Carvedilol inhibits mitochondrial complex I and induces resistance to H2O2 -mediated oxidative insult in H9C2 myocardial cells. [*Biochim Biophys Acta*.](https://www.ncbi.nlm.nih.gov/pubmed/17346667) 1767: 222-32.  [Yao K](https://www.ncbi.nlm.nih.gov/pubmed/?term=Yao%20K%5BAuthor%5D&cauthor=true&cauthor_uid=26797282), [Zhang WW](https://www.ncbi.nlm.nih.gov/pubmed/?term=Zhang%20WW%5BAuthor%5D&cauthor=true&cauthor_uid=26797282), [Yao L](https://www.ncbi.nlm.nih.gov/pubmed/?term=Yao%20L%5BAuthor%5D&cauthor=true&cauthor_uid=26797282) et al (2016) Carvedilol promotes mitochondrial biogenesis by regulating the PGC-1/TFAM pathway in human umbilical vein endothelial cells (HUVECs). [*Biochem Biophys Res Commun*.](https://www.ncbi.nlm.nih.gov/pubmed/26797282) 470: 961-6. |

| **Bisphosphonates** |
| --- |
| **Mode of action** |
| The goal is suppression of bone turnover. Based on mode of action bisphosphonates are divided into two classes, non-nitrogenous and nitrogenous.  The non-nitrogenous (Non-*N*-containing) bisphosphonates (non-N-BP) are:   - [Etidronate](https://en.wikipedia.org/wiki/Etidronic_acid) - [Clodronate](https://en.wikipedia.org/wiki/Clodronate) - [Tiludronate](https://en.wikipedia.org/wiki/Tiludronate)   The non-nitrogenous bisphosphonates are [metabolized](https://en.wikipedia.org/wiki/Metabolism) in the [cell](https://en.wikipedia.org/wiki/Cell_(biology)) to compounds that replace the terminal pyrophosphate moiety of ATP, forming a toxic analog that competes with [adenosine triphosphate](https://en.wikipedia.org/wiki/Adenosine_triphosphate) (ATP) in the cellular energy metabolism. Competitive inhibition of the ANT translocase. The osteoclast initiates [apoptosis](https://en.wikipedia.org/wiki/Apoptosis) and dies, leading to an overall decrease in the breakdown of bone. This type of bisphosphonate has overall more negative effects than the nitrogen containing group, and is prescribed far less often.  The nitrogenous bisphosphonates (N-BP) are:   - [Pamidronate](https://en.wikipedia.org/wiki/Pamidronate) - [Neridronate](https://en.wikipedia.org/wiki/Neridronate) - [Olpadronate](https://en.wikipedia.org/wiki/Olpadronate) - [Alendronate](https://en.wikipedia.org/wiki/Alendronate) - [Ibandronate](https://en.wikipedia.org/wiki/Ibandronate) - [Risedronate](https://en.wikipedia.org/wiki/Risedronate)   Nitrogenous bisphosphonates act on bone metabolism by binding and blocking directly the enzyme [farnesyl diphosphate synthase](https://en.wikipedia.org/wiki/Farnesyl_diphosphate_synthase) (FPPS) in the [HMG-CoA reductase pathway](https://en.wikipedia.org/wiki/HMG-CoA_reductase_pathway) (the mevalonate pathway). Disruption of the HMG CoA-reductase pathway at the level of FPPS leads to inhibition of prenylation of cellular proteins, which is essential for the functioning of GTPases and thus, cell survival. Next to this indirect effect, a direct effect has been described, namely inhibition of ANT (adenine nucleotide translocase) by ApppI, a new type of ATP analog. This analog is a product of the inhibition of FFP synthase. Inhibition of FFPS leads to accumulation of IPP which then seems to be converted to AppI.  Order of inhibitor potency against FFPS: alendronate < ibandronate < risedronate < zoledronic acid. |
| **Theoretical effects on mitochondrial function** |
| The non-nitrogenous BP could negatively affect the end-product of energy metabolism by competition with ATP at ANT translocase level.  The nitrogenous BP could lead to mitochondrial membrane instability and / or disturbed permeability and by competition with ATP at ANT translocase level. |
| **Known effects on mitochondrial function** |
| Induction of lipid peroxidation by producing mitochondrial superoxide. Activation of caspase-3-like enzymes. |
| **Effects measured/seen in mitochondrial patients, mitochondrial cell lines or normal cell lines** |
| The non-N-BP:   - Isolated rat liver mitochondria. In submitochondrial particles non-N-BP’s had no effect on mitochondrial oxygen consumption. However, the clodronate metabolite AppCCl2p caused a complete inhibition of the ADP stimulation of oxygen consumption. The same accounts for measurements of the membrane potential, no effect by clodronate, but an increase of the potential by AppCCl2p. No effect on complex V.   The N-BP:   - In gastrointestinal mucosal cells. Reduction of mitochondrial transmembrane potential, disruption of mitochondrial membrane and induction of the production of oygen radicals. Treatment with GGA (geranylgeranylacetone) reduced the production of oxygen radicals. - In renal epithelial cells. Expression of BAX (pro-apoptotic protein) and Bcl2 (anti-apoptotic protein) proteins showed an increase in the BAX/Bcl2 ratio, causing an elevation in the mitochondrial permeability of cells. - Isolated rat liver mitochondria. AppI inhibits ADP/ATP translocase. Zoledronic acid did not have an effect on ADP/ATP translocase, indicating that N-BPs themselves do not affect it. ApppI should reach intracellular concentration high enough to inhibit ANT activity in cells. The cellular uptake of N-BP and the activity of the mevalonate pathway are probably the critical points for ApppI production and thus for the inhibition of ANT. - Case reports. Dose-dependent nephrotoxicity of pamidronate is known. Mitochondrial changes in kidney biopsies: pronounced increase in number of mitochondria, variation in shape and size, degenerative changes (vacuolization, loss of cristae). |
| **References** |
| [Benford HL](https://www.ncbi.nlm.nih.gov/pubmed/?term=Benford%20HL%5BAuthor%5D&cauthor=true&cauthor_uid=10385693), [Frith JC](https://www.ncbi.nlm.nih.gov/pubmed/?term=Frith%20JC%5BAuthor%5D&cauthor=true&cauthor_uid=10385693), [Auriola S](https://www.ncbi.nlm.nih.gov/pubmed/?term=Auriola%20S%5BAuthor%5D&cauthor=true&cauthor_uid=10385693) et al (1999) Farnesol and geranylgeraniol prevent activation of caspases by aminobisphosphonates: biochemical evidence for two distinct pharmacological classes of bisphosphonate drugs. [*Mol Pharmacol.*](https://www.ncbi.nlm.nih.gov/pubmed/?term=benford+aminobiphosphanates) 56:131-40.  Lehenkari PP, Kellinsalmi M, Näpänkangas JP et al (2002) Further insight into mechanism of action of clodronate: inhibition of mitochondrial ADP/ATP translocase by a nonhydrolyzable, adenine-containing metabolite. *Mol Pharmacol.* 62: 1255-62.  [Markowitz GS](https://www.ncbi.nlm.nih.gov/pubmed/?term=Markowitz%20GS%5BAuthor%5D&cauthor=true&cauthor_uid=11373339), [Appel GB](https://www.ncbi.nlm.nih.gov/pubmed/?term=Appel%20GB%5BAuthor%5D&cauthor=true&cauthor_uid=11373339), [Fine PL](https://www.ncbi.nlm.nih.gov/pubmed/?term=Fine%20PL%5BAuthor%5D&cauthor=true&cauthor_uid=11373339) (2001) Collapsing focal segmental glomerulosclerosis following treatment with high-dose pamidronate. [*J Am Soc Nephrol.*](https://www.ncbi.nlm.nih.gov/pubmed/11373339) 12: 1164-72.  [Mönkkönen H](https://www.ncbi.nlm.nih.gov/pubmed/?term=M%C3%B6nkk%C3%B6nen%20H%5BAuthor%5D&cauthor=true&cauthor_uid=16402039), [Auriola S](https://www.ncbi.nlm.nih.gov/pubmed/?term=Auriola%20S%5BAuthor%5D&cauthor=true&cauthor_uid=16402039), [Lehenkari P](https://www.ncbi.nlm.nih.gov/pubmed/?term=Lehenkari%20P%5BAuthor%5D&cauthor=true&cauthor_uid=16402039) et al (2006) A new endogenous ATP analog (ApppI) inhibits the mitochondrial adenine nucleotide translocase (ANT) and is responsible for the apoptosis induced by nitrogen-containing bisphosphonates. [*Br J Pharmacol*.](https://www.ncbi.nlm.nih.gov/pubmed/16402039) 147: 437-45.  [Nagano Y](https://www.ncbi.nlm.nih.gov/pubmed/?term=Nagano%20Y%5BAuthor%5D&cauthor=true&cauthor_uid=23170047), [Matsui H](https://www.ncbi.nlm.nih.gov/pubmed/?term=Matsui%20H%5BAuthor%5D&cauthor=true&cauthor_uid=23170047), [Shimokawa O](https://www.ncbi.nlm.nih.gov/pubmed/?term=Shimokawa%20O%5BAuthor%5D&cauthor=true&cauthor_uid=23170047) (2012) Bisphosphonate-induced gastrointestinal mucosal injury is mediated by mitochondrial superoxide production and lipid peroxidation. [*J Clin Biochem Nutr*.](https://www.ncbi.nlm.nih.gov/pubmed/23170047) 51: 196-203.  [Reszka AA](https://www.ncbi.nlm.nih.gov/pubmed/?term=Reszka%20AA%5BAuthor%5D&cauthor=true&cauthor_uid=16036064), [Rodan GA](https://www.ncbi.nlm.nih.gov/pubmed/?term=Rodan%20GA%5BAuthor%5D&cauthor=true&cauthor_uid=16036064) (2003) Mechanism of action of bisphosphonates. [*Curr Osteoporos Rep*.](https://www.ncbi.nlm.nih.gov/pubmed/16036064) 1: 45-52.  [Sauter M](https://www.ncbi.nlm.nih.gov/pubmed/?term=Sauter%20M%5BAuthor%5D&cauthor=true&cauthor_uid=16731304), [Jülg B](https://www.ncbi.nlm.nih.gov/pubmed/?term=J%C3%BClg%20B%5BAuthor%5D&cauthor=true&cauthor_uid=16731304), [Porubsky S](https://www.ncbi.nlm.nih.gov/pubmed/?term=Porubsky%20S%5BAuthor%5D&cauthor=true&cauthor_uid=16731304) et al (2006) Nephrotic-range proteinuria following pamidronate therapy in a patient with metastatic breast cancer: mitochondrial toxicity as a pathogenetic concept? [*Am J Kidney Dis.*](https://www.ncbi.nlm.nih.gov/pubmed/16731304) 47: 1075-80.  [Singireesu SSNR](https://www.ncbi.nlm.nih.gov/pubmed/?term=Singireesu%20SSNR%5BAuthor%5D&cauthor=true&cauthor_uid=29729261), [Mondal SK](https://www.ncbi.nlm.nih.gov/pubmed/?term=Mondal%20SK%5BAuthor%5D&cauthor=true&cauthor_uid=29729261), [Yerramsetty S](https://www.ncbi.nlm.nih.gov/pubmed/?term=Yerramsetty%20S%5BAuthor%5D&cauthor=true&cauthor_uid=29729261) et al (2018) Zoledronic acid induces micronuclei formation, mitochondrial-mediated apoptosis and cytostasis in kidney cells. [*Life Sci*.](https://www.ncbi.nlm.nih.gov/pubmed/29729261) 203: 305-14. |

| **Bupivacaine** |
| --- |
| **Mode of action** |
| Bupivacaine is an amide-type, long-acting local anesthetic. Bupivicaine reversibly binds to specific sodium ion channels in the neuronal membrane, resulting in a decrease in the voltage-dependent membrane permeability to sodium ions and membrane stabilization; inhibition of depolarization and nerve impulse conduction; and a reversible loss of sensation.  In general, the progression of anesthesia is related to the diameter, myelination and conduction velocity of affected nerve fibers. Clinically, the order of loss of nerve function is as follows: (1) pain, (2) temperature, (3) touch, (4) proprioception, and (5) skeletal muscle tone. The analgesic effects of Bupivicaine are thought to potentially be due to its binding to the prostaglandin E2 receptors, subtype EP1 (PGE2EP1), which inhibits the production of prostaglandins, thereby reducing fever, inflammation, and hyperalgesia. |
| **Theoretical effects on mitochondrial function** |
| Active oxidative metabolism is a key determinant in bupivacaine toxicity. Bupivacaine can inhibit the electron transport chain by uncoupling of oxidative phosphorylation, which is a well-defined mechanism for mitochondrial toxicity. |
| **Known effects on mitochondrial function** |
| Bupivacaine inhibits carnitine acylcarnitine translocase (Weinberg et al 2000). Patients with defects in fatty acid metabolism may have an increased sensitivity to toxicity from bupivacaine.  Bupivacaine depresses cardiac automaticity, conductivity and contractility, but the predominant effect is contractile dysfunction, which results from the disruption of mitochondrial energy metabolism. β-adrenergic activation aggravates the cellular metabolism disorder and therefore contractile dysfunction (Li et al 2018).  Active oxidative metabolism is a key determinant in bupivacaine toxicity. Bupivacaine  myotoxicity is a relevant model of mitochondrial dysfunction involving the PTP and Ca(2+) dysregulation (Irwin et al 2002).  Mitochondrial anion channel and p38 MAPK pathway are implicated in bupicavaine-induced apoptosis. Bupivacaine-induced reactive oxygen species production results in an alteration in the permeability of the mitochondrial membranes and Cl(-) influx into mitochondria, which seems to be responsible for mitochondrial depolarization and the p38 MAPK activation (Lu et al 2011).  Bupivacaine was able to induce lysosomal membrane permeabilization (LMP) with the release of cathepsins into the cytosol, as evidenced by LysoTracker Red staining, acridine orange staining, and cathepsin D immunofluorescence staining. Consistently, inhibitors of lysosomal cathepsins, CA074-Me and pepstatin A, significantly reduced bupivacaine-induced cell death. Finally, we found that bupivacaine resulted in an increase in intracellular reactive oxygen species (ROS) and that inhibition of ROS by N-acetyl-L-cysteine effectively blocked bupivacaine-induced LMP and cell death (Cai et al 2018). |
| **Effects measured/seen in mitochondrial patients, mitochondrial cell lines or normal cell lines** |
| Regional anesthetic techniques and wound infiltration with local anesthetics provide analgesia without the inhibitory effect of parenteral opioids on respiratory drive and upper airway tone. There is a single report of ventricular dysrhythmia after a patient with carnitine deficiency received a small dose of bupivacaine (an amide local anesthetic), which may have been caused by the inhibition of carnitine–acylcarnitine translocase that occurs with that particular local anesthetic in vitro. It should be noted that other clinically useful amide local anesthetics (ropivacaine and lidocaine) inhibit carnitine–acylcarnitine translocase to a lesser degree and therefore have less of a deleterious effect on carnitine-stimulated pyruvate oxidation than does bupivacaine. They should therefore be chosen over bupivacaine when nerve blocks are performed in children with mitochondrial disease. |
| **References** |
| Cai X, Liu Y, Hu Y et al (2018) ROS-mediated lysosomal membrane permeabilization is involved in bupivacaine-induced death of rabbit intervertebral disc cells. *Redox Biol.* 18: 65-76.  Chen Z, Jin Z, Xia Y et al (2017) The protective effect of lipid emulsion in preventing bupivacaine-induced mitochondrial injury and apoptosis of H9C2 cardiomyocytes. *Drug Deliv.* 24: 430-436.  Grishko V, Xu M, Wilson G et al (2010) Apoptosis and mitochondrial dysfunction in human chondrocytes following exposure to lidocaine, bupivacaine, and ropivacaine. *J Bone Joint Surg Am*. 92: 609-18.  Irwin W, Fontaine E, Agnolucci L et al (2002) Bupivacaine myotoxicity is mediated by mitochondria. *J Biol Chem*. 277: 12221-7.  Li J, Duan R, Zhang Y et al (2018) Beta-adrenergic activation induces cardiac collapse by aggravating cardiomyocyte contractile dysfunction in bupivacaine intoxication. *PLoS One* 13(10): e0203602. eCollection 2018.  Lu J, Xu SY, Zhang QG et al (2011) Bupivacaine induces apoptosis via mitochondria and p38 MAPK dependent pathways. *Eur J Pharmacol.* 657(1-3): 51-8.  Weinberg GL, Palmer JW, VadeBoncouer TR et al (2000) Bupivacaine inhibits acylcarnitine exchange in cardiac mitochondria. *Anesthesiology.* 92: 523-8. |

| **Carbamazepine** |
| --- |
| **Mode of action** |
| Carbamazepine is a sodium channel blocker which preferentially binds to inactive voltage-gated sodium channels and prevents rapid repetitive firing of action potentials in neurons. The chemical name is 5H-dibenzazepine-5-carboxamide and the principal metabolite, carbamazepine-10,11-epoxide, is also thought to have an anticonvulsant action. MoA as a mood stabiliser is unknown though it is thought to affect the serotonergic system (Dailey et al 1998). |
| **Theoretical effects on mitochondrial function** |
| Finsterer makes repeated reference to the toxic effect of carbamazepine on mitochondria in several review articles(Finsterer et al 2012, 2012, 2017). The only evidence Finsterer refers to is a publication by Santos *et al* in rat liver (Santos 2008). Santos *et al* actually concluded that of the 3 drugs they investigated, phenytoin, phenobarbital and carbamazepine, the latter was the least effective in impairing mitochondrial function. |
| **Known effects on mitochondrial function** |
| None |
| **Effects measured/seen in mitochondrial patients, mitochondrial cell lines or normal cell lines** |
| None |
| **References** |
| Dailey JW, Reith ME, Steidley KR et al (1998) Carbamazepine-induced release of serotonin from rat hippocampus in vitro. *Epilepsia* 39: 1054-63.  Finsterer J, Scorza FA (2017) [Epilepsy in mitochondrial disorders.](https://www.ncbi.nlm.nih.gov/pubmed/22459315) *Epilepsy Res*. 136: 5-11.  Finsterer J, Zarrouk Mahjoub S (2012) [Effects of antiepileptic drugs on mitochondrial functions, morphology, kinetics, biogenesis, and survival.](https://www.ncbi.nlm.nih.gov/pubmed/28732239) *Seizure.* 21: 316-21.  Finsterer J, Zarrouk Mahjoub S (2012) [Mitochondrial toxicity of antiepileptic drugs and their tolerability in mitochondrial disorders.](https://www.ncbi.nlm.nih.gov/pubmed/22149023) *Expert Opin Drug Metab Toxicol*. 8: 71-9.  Santos NA, Medina WS, Martins NM et al (2008) Aromatic antiepileptic drugs and mitochondrial toxicity: effects on mitochondria isolated from rat liver. *Toxicol In Vitro*, 22: 1143-52. |

| **Ceftriaxone** |
| --- |
| **Mode of action** |
| A broad-spectrum cephalosporin antibiotic. Works by inhibiting the mucopeptide synthesis in the bacterial cell wall. The beta-lactam moiety of ceftriaxone binds to carboxypeptidases, endopeptidases, and transpeptidases in the bacterial cytoplasmic membrane. These enzymes are involved in cell-wall synthesis and cell division. By binding to these enzymes, Ceftriaxone results in the formation of of defective cell walls and cell death.  Thirty-three percent to 67% of a ceftriaxone dose was excreted in the urine as unchanged drug and the remainder was secreted in the bile and ultimately found in the feces as microbiologically inactive compounds. |
| **Theoretical effects on mitochondrial function** |
| The beta-lactam probably binds to peptidases in mitochondrial membranes leading to defective mitochondrial membranes and mitochondrial dysfunction. |
| **Known effects on mitochondrial function** |
| No known effects of ceftriaxone on mitochondrial function. Regarding the group of beta-lactam antibiotics it is known that they induce an increase in ROS production. |
| **Effects measured/seen in mitochondrial patients, mitochondrial cell lines or normal cell lines** |
| Effects measured beta-lactam antibiotics:   - On isolated ETC protein complexes. Inhibition mitochondrial ETC complexes, in particular complexes I and III, complex V showing less change in activity. Resulting in ROS formation. (ampicillin) - In primary human mammary epithelial cells (MCF10A cells). Effect on balance between fission and fusion. Short, swollen, fragmented mitochondria with highly reduced branching. (ampicillin) - In MCF10A cells. Seahorse experiment showed reduced basal respiration and maximal respiratory capacity. (ampicillin) - Inhibition of the carnitine/acylcarnitine transporter. Inhibition of the transporter competing with the physiological substrate for the active site or other cephalosporins inhibit the transporter by covalent interaction. The irreversible mechanism may become significant for some of the cephalosporins after medium and long term administration. Cefonicid, cefazolin, cephalotin, ampicillin, piperacillin inhibited the carnitine transport at various extent; whereas cefepime, cefotaxime, ceftriaxone, cefuroxime and cefodizime had very low, if any, effect. - Beta-lactam antibiotics are potent stimulators of GLT1 expression. It appears to be mediated through increased transcription of the GLT1 gene. When delivered to animals, the beta-lactam ceftriaxone increased both brain expression of GLT1 and its biochemical and functional activity. Glutamate transporters are important in preventing glutamate neurotoxicity. Ceftriaxone was neuroprotective in vitro when used in models of ischaemic injury and motor neuron degeneration, both based in part on glutamate toxicity. Animal studies concerning ischemic injury and ALS. |
| **References** |
| [Kalghatgi S](https://www.ncbi.nlm.nih.gov/pubmed/?term=Kalghatgi%20S%5BAuthor%5D&cauthor=true&cauthor_uid=23825301), [Spina CS](https://www.ncbi.nlm.nih.gov/pubmed/?term=Spina%20CS%5BAuthor%5D&cauthor=true&cauthor_uid=23825301), [Costello JC](https://www.ncbi.nlm.nih.gov/pubmed/?term=Costello%20JC%5BAuthor%5D&cauthor=true&cauthor_uid=23825301) (2013) Bactericidal antibiotics induce mitochondrial dysfunction and oxidative damage in Mammalian cells. [*Sci Transl Med.*](https://www.ncbi.nlm.nih.gov/pubmed/23825301) 5: 192.  [Kohanski MA](https://www.ncbi.nlm.nih.gov/pubmed/?term=Kohanski%20MA%5BAuthor%5D&cauthor=true&cauthor_uid=17803904), [Dwyer DJ](https://www.ncbi.nlm.nih.gov/pubmed/?term=Dwyer%20DJ%5BAuthor%5D&cauthor=true&cauthor_uid=17803904), [Hayete B](https://www.ncbi.nlm.nih.gov/pubmed/?term=Hayete%20B%5BAuthor%5D&cauthor=true&cauthor_uid=17803904) et al (2007) A common mechanism of cellular death induced by bactericidal antibiotics. [*Cell*.](https://www.ncbi.nlm.nih.gov/pubmed/17803904) 130: 797-810.  [Pochini L](https://www.ncbi.nlm.nih.gov/pubmed/?term=Pochini%20L%5BAuthor%5D&cauthor=true&cauthor_uid=18452908), [Galluccio M](https://www.ncbi.nlm.nih.gov/pubmed/?term=Galluccio%20M%5BAuthor%5D&cauthor=true&cauthor_uid=18452908), [Scumaci D](https://www.ncbi.nlm.nih.gov/pubmed/?term=Scumaci%20D%5BAuthor%5D&cauthor=true&cauthor_uid=18452908) et al (2008) Interaction of beta-lactam antibiotics with the mitochondrial carnitine/acylcarnitine transporter. [*Chem Biol Interact.*](https://www.ncbi.nlm.nih.gov/pubmed/18452908) 173: 187-94.  [Rothstein JD](https://www.ncbi.nlm.nih.gov/pubmed/?term=Rothstein%20JD%5BAuthor%5D&cauthor=true&cauthor_uid=15635412), [Patel S](https://www.ncbi.nlm.nih.gov/pubmed/?term=Patel%20S%5BAuthor%5D&cauthor=true&cauthor_uid=15635412), [Regan MR](https://www.ncbi.nlm.nih.gov/pubmed/?term=Regan%20MR%5BAuthor%5D&cauthor=true&cauthor_uid=15635412) et al (2005) Beta-lactam antibiotics offer neuroprotection by increasing glutamate transporter expression. [*Nature*.](https://www.ncbi.nlm.nih.gov/pubmed/15635412) 433: 73-7.  Tune BM, Sibley RK, Hsu CY (1988) The Mitochondrial Respiratory Toxicity of Cephalosporin Antibiotics. An Inhibitory Effect on Substrate Uptake. *J Pharmacol Exp Ther* 245: 1054–9.  Tune BM, Hsu CY (1990) The Renal Mitochondrial Toxicity of Cephalosporins: Specificity of the Effect on Anionic Substrate Uptake. *J Pharmacol Exp Ther* 252: 65–9. |

| **Chloramphenicol** |
| --- |
| **Mode of action** |
| Chloramphenicol (CAP) is commonly used topically to treat eye infections but is still used in some developing countries intravenously in the form of CAP succinate to treat haemophilus influenzae meningitis due to high central nervous system penetration and resistant strains to other newer antibiotics. Its mechanism of antimicrobial action is to bind the 50S bacterial ribosomal  Known patterns of toxicity:   1. Myelotoxicity: At lower blood concentrations – a reversible dose dependent cell differentiation arrest of bone marrow erythroid precursors (2) 2. Gray baby syndrome: Ineffective clearance/conjugation of the drug by the neonatal liver results in extremely high blood levels and multi-organ failure (3) 3. Fatal aplastic crises in certain genetically susceptible individuals (1,4) |
| **Theoretical effects on mitochondrial function** |
| 1. Inhibition of ribosomal translation by binding rRNA in 50S of mitoribosomes(1,5,6) specifically of cytochromes a (2,6), a3 (2,6)(Complex IV), b(2,6) and c1 (Complex III)(6) 2. Reduction of mitochondrial DNA transcription(7) 3. Complex I inhibition (8) 4. Inhibition of SDH (Complex II) (9) 5. Structural changes: Megalomitochondria (10,11)   formation and abnormal cristae (6,7), vacuolation (3)  6. Autophagy (1) |
| **Known effects on mitochondrial function** |
| \| **Type of Study** \| **Model Used** \| **Effect Measured** \| **Effect seen/ Mechanism** \| **Ref** \| \| --- \| --- \| --- \| --- \| --- \| \| **In vitro** \| Rabbit bone marrow cells and peripheral erythrocytes  Rabbit bone marrow mitochondria \| Protein synthesis cyt a, a3 and b.  COX activity  O2 consumption \| Reduced COX activity, cyt a, a3, b (reversible)  Normal SDH activity noted  Reduced incorporation of radiolabeled amino acids in mitochondria  Reduced O2 consumption rate in marrow cells (reversible) \| **2** \| \| **In vivo** \| Rabbits \| Survival \| Reversible myelosuppression after exposure to CAP infusion up to 5days, death beyond 5 days of exposure \| **2** \| \| **Ex vivo** \| Porcine Cardiac mitochondria \| Mito respiration \| Mitochondrial respiration reduced \| **3** \| \| **Ex vivo** \| Rat hepatocytes \| Protein synthesis cyt a, a3, b, c1  COX activity  Mito morphology \| Reduction in Cyt a, a3, b, c1 but not c  Reduced COX activity  Mitochondrial swelling, cytoplasmic vacuolation, reduced mitochondrial cristae and dilated ER cisternae \| **6** \| \| **In vivo** \| Rat- partially hepatectomised \| Liver regeneration \| Rate of liver regeneration reduced in the presence of CAP \| **6** \| \| **In vitro** \| Human fibroblasts \| mtDNA transcription  Mito morphology \| Reduced ND1, ND4, COX2 RNA transcripts  Increased POLG and TFAM RNA transcripts (likely compensatory)  Abnormal cristae \| **7** \| \| **In vivo** \| Rat liver \| NADH levels  Survival \| Complex I inhibition  Reduced survival \| **8** \| \| In vitro \| Human Bone marrow cells, hepatocytes  Rat Liver and kidney mitochondria \| FADH2 formation \| CAP is shown to be a competitive inhibitor of FAD by acting at the SDH reduction site. Therefore SDH activity is reduced (not consistent with ( ref **2**)) \| **9** \| \| **In vitro** \| Rat hepatocytes \| ROS levels  Mito morphology  Cell growth \| Elevated levels of ROS, Megalomitochondria, Reduced cell growth: induction of apoptosis thought to be due to reduced ATP \| **10** \| \| **Ex vivo** \| Rat hepatocytes \| Liver growth  Mito morphology  ROS levels \| Reduced wt and liver size  Megalomitochondria,  Increased ROS \| **11** \| \| In vitro \| Rat liver and brain mitochondria \| Protein synthesis \| reduced mitochondrial protein synthesis: reduced incorporation of radiolabelled amino acids \| **12** \| \| **In vitro** \| Mitochondria isolated from:  Rat heart and liver  rabbit heart and bone marrow \| Total mito protein synthesis \| Reduced total mitochondrial protein levels \| **13** \| |
| **Effects measured/seen in mitochondrial patients, mitochondrial cell lines or normal cell lines** |
| It is hypothesized that CAP toxicity resulting in fatal aplastic anaemia may be a phenomenon that is due to a genetic predisposition due to mtDNA mutations, and therefore that CAP sensitivity may be a feature of certain mitochondrial diseases. One case report of severe aplastic anaemia in an individual with m.3010G>A(mitochondrial rRNA gene) has been described(14), and another of identical twins with CAP induced AA although no genetic diagnosis was sought in this case(15). The experiments above provide several theoretical mechanisms that link CAP toxicity to mitochondrial dysfunction. However, they are not translatable to mitochondrial patients as none of them were performed using tissue from animals/patients with mutations known to cause mitochondrial disease. Only two studies were in human cell lines (in vitro) and all in vivo experiments were in animals. The in vivo experiments are supportive of the hypothesis that CAP induced mitochondrial dysfunction mediates (at least in part) its known toxic effects. |
| **References** |
| 1. Cohen BH, Saneto RP (2012) [Mitochondrial translational inhibitors in the pharmacopeia.](https://www.ncbi.nlm.nih.gov/pubmed/22421540) *Biochim Biophys Acta*. 1819: 1067-74. 2. Firkin FC (1972) [Mitochondrial lesions in reversible erythropoietic depression due to chloramphenicol.](https://www.ncbi.nlm.nih.gov/pubmed/4341013) *J Clin Invest*. 51: 2085-92. 3. Fripp RR, Carter MC, Werner JC et al (1983) [Cardiac function and acute chloramphenicol toxicity.](https://www.ncbi.nlm.nih.gov/pubmed/6886920) *J Pediatr*. 103: 487-90. 4. Fine PE (1978) [Mitochondrial inheritance and disease.](https://www.ncbi.nlm.nih.gov/pubmed/80581) *Lancet*. 2: 659-62. 5. Barnhill AE, Brewer MT, Carlson SA(2012) [Adverse effects of antimicrobials via predictable or idiosyncratic inhibition of host mitochondrial components.](https://www.ncbi.nlm.nih.gov/pubmed/22615289) *Antimicrob Agents Chemother*. 56: 4046-51 6. Firkin FC, Linnane AW (1969) [Biogenesis of mitochondria. The effect of chloramphenicol on regenerating rat liver.](https://www.ncbi.nlm.nih.gov/pubmed/5780794) *Exp Cell Res.* 55: 68-76. 7. Kao LP, Ovchinnikov D, Wolvetang E (2012) [The effect of ethidium bromide and chloramphenicol on mitochondrial biogenesis in primary human fibroblasts.](https://www.ncbi.nlm.nih.gov/pubmed/22712077) *Toxicol Appl Pharmacol*. 261: 42-9. 8. Mottin S, Laporte P, Cespuglio R (2003) [Inhibition of NADH oxidation by chloramphenicol in the freely moving rat measured by picosecond time-resolved emission spectroscopy.](https://www.ncbi.nlm.nih.gov/pubmed/12562508) *J Neurochem*. 84: 633-42. 9. Ambekar CS, Lee JS, Cheung BM et al (2004) [Chloramphenicol succinate, a competitive substrate and inhibitor of succinate dehydrogenase: possible reason for its toxicity.](https://www.ncbi.nlm.nih.gov/pubmed/15130601) *Toxicol In Vitro.* 18: 441-7 10. Karbowski M, Kurono C, Wozniak M et al (1999) [Cycloheximide and 4-OH-TEMPO suppress chloramphenicol-induced apoptosis in RL-34 cells via the suppression of the formation of megamitochondria.](https://www.ncbi.nlm.nih.gov/pubmed/10076048) *Biochim Biophys Acta.* 1449: 25-40. 11. Matsuhashi T, Liu X, Nishizawa Y et al (1996) Mechanism of the formation of megamitochondria in the mouse liver induced by chloramphenicol. *Toxicol Lett*. 86: 47-54. 12. Bernacki RJ, Bosmann HB (1970) [Warfarin and vitamin K accelerate protein and glycoprotein synthesis in isolated rat liver mitochondria in vitro.](https://www.ncbi.nlm.nih.gov/pubmed/5518179) *Biochem Biophys Res Commun*. 41: 498-505. 13. McKee EE, Ferguson M, Bentley AT et al (2006) [Inhibition of mammalian mitochondrial protein synthesis by oxazolidinones.](https://www.ncbi.nlm.nih.gov/pubmed/16723564) *Antimicrob Agents Chemother.* 50: 2042-9. 14. Mehta A, Vulliamy T, Gordon-Smith E et al (1989) A new genetic polymorphism in the 16S ribosomal RNA gene of human mitochondrial DNA. *Ann Hum Genet* 53: 303–310. 15. Nagao T, Mauer A (1969) Concordance for drug-induced aplastic anaemia in identical twins. *N Engl J Med* 281: 7–11. |

| **Clozapine** |
| --- |
| **Mode of action** |
| 1. < 10 nM affinity for the serotonin (5-HT)2A, 5-HT2C, 5-HT6, 5-HT7, D4, m1, and alpha 1-adrenergic receptor 2. Weak affinity for the D2 receptor. 3. Current evidence suggests the 5-HT2A and D4 receptor antagonist properties of clozapine, together with its weak D2 blocking properties, contribute the most to its MOA |
| **Theoretical effects (on mitochondrial function)** |
| 1. Directly increases *de novo* lipogenesis in [hepatocytes](https://www.sciencedirect.com/topics/medicine-and-dentistry/hepatocyte) 2. Acute clozapine exposure affects SREBP-regulated lipid biosynthesis as well as other lipid homeostasis pathways 3. Increased risk of metabolic syndrome 4. Decreases the plasma level of insulin and causes hyperglycaemia and insulin resistance in rats |
| **Known effects (on mitochondrial function)** |
| 1. Mechanisms involved are not fully characterized; it appears that overproduction of reactive oxygen species by the damaged mitochondria could play a salient role. 2. Inhibits complex I, but at a significantly higher concentration 3. Altered morphology, membrane potential & reduced ATP levels 4. Inhibition of ETC & OXPHOS capacity 5. Slight effects on NADH-coenzyme Q reductase activity |
| **References** |
| [Chen J](https://www.ncbi.nlm.nih.gov/pubmed/?term=Chen%20J%5BAuthor%5D&cauthor=true&cauthor_uid=29209160), [Huang XF](https://www.ncbi.nlm.nih.gov/pubmed/?term=Huang%20XF%5BAuthor%5D&cauthor=true&cauthor_uid=29209160), [Shao R](https://www.ncbi.nlm.nih.gov/pubmed/?term=Shao%20R%5BAuthor%5D&cauthor=true&cauthor_uid=29209160) et al (2017) Molecular Mechanisms of Antipsychotic Drug-Induced Diabetes. [*Front Neurosci.*](https://www.ncbi.nlm.nih.gov/pubmed/?term=29209160) 11: 643.  [Contreras-Shannon V](https://www.ncbi.nlm.nih.gov/pubmed/?term=Contreras-Shannon%20V%5BAuthor%5D&cauthor=true&cauthor_uid=23527073), [Heart DL](https://www.ncbi.nlm.nih.gov/pubmed/?term=Heart%20DL%5BAuthor%5D&cauthor=true&cauthor_uid=23527073), [Paredes RM](https://www.ncbi.nlm.nih.gov/pubmed/?term=Paredes%20RM%5BAuthor%5D&cauthor=true&cauthor_uid=23527073) et al (2013) Clozapine-induced mitochondria alterations and inflammation in brain and insulin-responsive cells. [*PLoS One*.](https://www.ncbi.nlm.nih.gov/pubmed/?term=23527073) 8: e59012.  [Elmorsy E](https://www.ncbi.nlm.nih.gov/pubmed/?term=Elmorsy%20E%5BAuthor%5D&cauthor=true&cauthor_uid=25824037), [Smith PA](https://www.ncbi.nlm.nih.gov/pubmed/?term=Smith%20PA%5BAuthor%5D&cauthor=true&cauthor_uid=25824037) (2015) Bioenergetic disruption of human micro-vascular endothelial cells by antipsychotics. [*Biochem Biophys Res Commun.*](https://www.ncbi.nlm.nih.gov/pubmed/?term=25824037) 460: 857-62.  [Elmorsy E](https://www.ncbi.nlm.nih.gov/pubmed/?term=Elmorsy%20E%5BAuthor%5D&cauthor=true&cauthor_uid=28322891), [Al-Ghafari A](https://www.ncbi.nlm.nih.gov/pubmed/?term=Al-Ghafari%20A%5BAuthor%5D&cauthor=true&cauthor_uid=28322891), [Aggour AM](https://www.ncbi.nlm.nih.gov/pubmed/?term=Aggour%20AM%5BAuthor%5D&cauthor=true&cauthor_uid=28322891) et al (2017) Effect of antipsychotics on mitochondrial bioenergetics of rat ovarian theca cells. [*Toxicol Lett.*](https://www.ncbi.nlm.nih.gov/pubmed/?term=28322891) 272: 94-100  [Fernø J](https://www.ncbi.nlm.nih.gov/pubmed/?term=Fern%C3%B8%20J%5BAuthor%5D&cauthor=true&cauthor_uid=18989661), [Vik-Mo AO](https://www.ncbi.nlm.nih.gov/pubmed/?term=Vik-Mo%20AO%5BAuthor%5D&cauthor=true&cauthor_uid=18989661), [Jassim G](https://www.ncbi.nlm.nih.gov/pubmed/?term=Jassim%20G%5BAuthor%5D&cauthor=true&cauthor_uid=18989661) et al (2009) Acute clozapine exposure in vivo induces lipid accumulation and marked sequential changes in the expression of SREBP, PPAR, and LXR target genes in rat liver. [*Psychopharmacology (Berl).*](https://www.ncbi.nlm.nih.gov/pubmed/?term=18989661)203: 73-84.  [Kumar P](https://www.ncbi.nlm.nih.gov/pubmed/?term=Kumar%20P%5BAuthor%5D&cauthor=true&cauthor_uid=30143692), [Efstathopoulos P](https://www.ncbi.nlm.nih.gov/pubmed/?term=Efstathopoulos%20P%5BAuthor%5D&cauthor=true&cauthor_uid=30143692), [Millischer V](https://www.ncbi.nlm.nih.gov/pubmed/?term=Millischer%20V%5BAuthor%5D&cauthor=true&cauthor_uid=30143692) et al (2018) Mitochondrial DNA copy number is associated with psychosis severity and anti-psychotic treatment. [*Sci Rep*.](https://www.ncbi.nlm.nih.gov/pubmed/?term=30143692) 8: 12743.  [Modica-Napolitano JS](https://www.ncbi.nlm.nih.gov/pubmed/?term=Modica-Napolitano%20JS%5BAuthor%5D&cauthor=true&cauthor_uid=14661862), [Lagace CJ](https://www.ncbi.nlm.nih.gov/pubmed/?term=Lagace%20CJ%5BAuthor%5D&cauthor=true&cauthor_uid=14661862), [Brennan WA](https://www.ncbi.nlm.nih.gov/pubmed/?term=Brennan%20WA%5BAuthor%5D&cauthor=true&cauthor_uid=14661862) et al (2003) Differential effects of typical and atypical neuroleptics on mitochondrial function in vitro. [*Arch Pharm Res*.](https://www.ncbi.nlm.nih.gov/pubmed/?term=14661862) 26: 951-9.  [Rowe C](https://www.ncbi.nlm.nih.gov/pubmed/?term=Rowe%20C%5BAuthor%5D&cauthor=true&cauthor_uid=28919358), [Shaeri M](https://www.ncbi.nlm.nih.gov/pubmed/?term=Shaeri%20M%5BAuthor%5D&cauthor=true&cauthor_uid=28919358), [Large E](https://www.ncbi.nlm.nih.gov/pubmed/?term=Large%20E%5BAuthor%5D&cauthor=true&cauthor_uid=28919358) et al (2018) Perfused human hepatocyte microtissues identify reactive metabolite-forming and mitochondria-perturbing hepatotoxins. [*Toxicol In Vitro*.](https://www.ncbi.nlm.nih.gov/pubmed/?term=28919358) 46: 29-38.  [Scaini G](https://www.ncbi.nlm.nih.gov/pubmed/?term=Scaini%20G%5BAuthor%5D&cauthor=true&cauthor_uid=29449054), [Quevedo J](https://www.ncbi.nlm.nih.gov/pubmed/?term=Quevedo%20J%5BAuthor%5D&cauthor=true&cauthor_uid=29449054), [Velligan D](https://www.ncbi.nlm.nih.gov/pubmed/?term=Velligan%20D%5BAuthor%5D&cauthor=true&cauthor_uid=29449054) et al (2018) Second generation antipsychotic-induced mitochondrial alterations: Implications for increased risk of metabolic syndrome in patients with schizophrenia. [*Eur Neuropsychopharmacol.*](https://www.ncbi.nlm.nih.gov/pubmed/?term=29449054) 28: 369-80.  [Tran HQ](https://www.ncbi.nlm.nih.gov/pubmed/?term=Tran%20HQ%5BAuthor%5D&cauthor=true&cauthor_uid=30207504), [Park SJ](https://www.ncbi.nlm.nih.gov/pubmed/?term=Park%20SJ%5BAuthor%5D&cauthor=true&cauthor_uid=30207504), [Shin EJ](https://www.ncbi.nlm.nih.gov/pubmed/?term=Shin%20EJ%5BAuthor%5D&cauthor=true&cauthor_uid=30207504) et al (2018) Clozapine attenuates mitochondrial burdens and abnormal behaviors elicited by phencyclidine in mice via inhibition of p47 ^phox^; Possible involvements of phosphoinositide 3-kinase/Akt signaling. [J Psychopharmacol.](https://www.ncbi.nlm.nih.gov/pubmed/?term=30207504) 2018 Nov;32(11):1233-51. |

| **Chlorpromazine** |
| --- |
| **Mode of action** |
| 1. Blocks post-synaptic D_2_ dopamine receptors. 2. Has anti-serotonergic & anti-histaminergic properties |
| **Theoretical effects on mitochondrial function** |
| Inhibition of ETC & OXPHOS capacity (Inhibit complexes I and III) |
| **Known effects on mitochondrial function** |
| 1. Impairs biogenesis in human microvascular endothelial cells of BBB (concn. and time dep manner) (Elmorsy and Smith 2015) 2. Oxidative stress plays a major role as both a primary causal and an aggravating factor in the early CPZ-induced intrahepatic cholestasis in human hepatocytes.(De Faria et al 2015) 3. Differential effects of typical and atypical neuroleptics on mitochondrial function in vitro. (Modica-Napolitano et al 2003) |
| **Effects measured/seen in mitochondrial patients, mitochondrial cell lines or normal cell lines** |
| Manuscripts   1. Bioenergetic disruption of human micro-vascular endothelial cells by antipsychotics. 2. Effect of antipsychotics on mitochondrial bioenergetics of rat ovarian theca cells. 3. Cytotoxicity of phenothiazine derivatives associated with mitochondrial dysfunction: a structure-activity investigation. (De Faria et al 2015) 4. Oxidative stress plays a major role in chlorpromazine-induced cholestasis in human HepaRG cells. (Anthérieu et al 2013) 5. Effect of chlorpromazine and biliary drainage on portal blood flow and mitochondrial function during extrahepatic cholestasis. (Roselino et al 1989) 6. Mechanism of chlorpromazine-induced arrhythmia -- arrhythmia and mitochondrial dysfunction. (Kitazawa et al 1981)   In vivo / in vitro / cell / animal studies  HBVECs (Elmorsy and Smith 2015); rat: (Elmorsy et al 2017); mongrel dogs: (Kitazawa et al 1981) |
| **References** |
| [Anthérieu S](https://www.ncbi.nlm.nih.gov/pubmed/?term=Anth%C3%A9rieu%20S%5BAuthor%5D&cauthor=true&cauthor_uid=23175273), [Bachour-El Azzi P](https://www.ncbi.nlm.nih.gov/pubmed/?term=Bachour-El%20Azzi%20P%5BAuthor%5D&cauthor=true&cauthor_uid=23175273), [Dumont J](https://www.ncbi.nlm.nih.gov/pubmed/?term=Dumont%20J%5BAuthor%5D&cauthor=true&cauthor_uid=23175273) et al (2013) Oxidative stress plays a major role in chlorpromazine-induced cholestasis in human HepaRG cells. [*Hepatology.*](https://www.ncbi.nlm.nih.gov/pubmed/?term=23175273) 57: 1518-29.  [De Faria PA](https://www.ncbi.nlm.nih.gov/pubmed/?term=de%20Faria%20PA%5BAuthor%5D&cauthor=true&cauthor_uid=25686698), [Bettanin F](https://www.ncbi.nlm.nih.gov/pubmed/?term=Bettanin%20F%5BAuthor%5D&cauthor=true&cauthor_uid=25686698), [Cunha RL](https://www.ncbi.nlm.nih.gov/pubmed/?term=Cunha%20RL%5BAuthor%5D&cauthor=true&cauthor_uid=25686698) et al (2015) Cytotoxicity of phenothiazine derivatives associated with mitochondrial dysfunction: a structure-activity investigation. [*Toxicology.*](https://www.ncbi.nlm.nih.gov/pubmed/?term=25686698) 330: 44-54.  [Elmorsy E](https://www.ncbi.nlm.nih.gov/pubmed/?term=Elmorsy%20E%5BAuthor%5D&cauthor=true&cauthor_uid=25824037), [Smith PA](https://www.ncbi.nlm.nih.gov/pubmed/?term=Smith%20PA%5BAuthor%5D&cauthor=true&cauthor_uid=25824037) (2015) Bioenergetic disruption of human micro-vascular endothelial cells by antipsychotics. [*Biochem Biophys Res Commun.*](https://www.ncbi.nlm.nih.gov/pubmed/?term=25824037) 460: 857-6.  [Elmorsy E](https://www.ncbi.nlm.nih.gov/pubmed/?term=Elmorsy%20E%5BAuthor%5D&cauthor=true&cauthor_uid=28322891), [Al-Ghafari A](https://www.ncbi.nlm.nih.gov/pubmed/?term=Al-Ghafari%20A%5BAuthor%5D&cauthor=true&cauthor_uid=28322891), [Aggour AM](https://www.ncbi.nlm.nih.gov/pubmed/?term=Aggour%20AM%5BAuthor%5D&cauthor=true&cauthor_uid=28322891) et al (2017) Effect of antipsychotics on mitochondrial bioenergetics of rat ovarian theca cells. [*Toxicol Lett.*](https://www.ncbi.nlm.nih.gov/pubmed/?term=28322891) 272: 94-100.  [Kitazawa M](https://www.ncbi.nlm.nih.gov/pubmed/?term=Kitazawa%20M%5BAuthor%5D&cauthor=true&cauthor_uid=6167651), [Sugiyama S](https://www.ncbi.nlm.nih.gov/pubmed/?term=Sugiyama%20S%5BAuthor%5D&cauthor=true&cauthor_uid=6167651), [Ozawa T](https://www.ncbi.nlm.nih.gov/pubmed/?term=Ozawa%20T%5BAuthor%5D&cauthor=true&cauthor_uid=6167651) et al (1981) Mechanism of chlorpromazine-induced arrhythmia -- arrhythmia and mitochondrial dysfunction. [*J Electrocardiol.*](https://www.ncbi.nlm.nih.gov/pubmed/?term=6167651) 14: 219-24.  [Modica-Napolitano JS](https://www.ncbi.nlm.nih.gov/pubmed/?term=Modica-Napolitano%20JS%5BAuthor%5D&cauthor=true&cauthor_uid=14661862), [Lagace CJ](https://www.ncbi.nlm.nih.gov/pubmed/?term=Lagace%20CJ%5BAuthor%5D&cauthor=true&cauthor_uid=14661862), [Brennan WA](https://www.ncbi.nlm.nih.gov/pubmed/?term=Brennan%20WA%5BAuthor%5D&cauthor=true&cauthor_uid=14661862) et al (2003) Differential effects of typical and atypical neuroleptics on mitochondrial function in vitro. [*Arch Pharm Res*.](https://www.ncbi.nlm.nih.gov/pubmed/?term=14661862) 26: 951-9.  [Roselino JE](https://www.ncbi.nlm.nih.gov/pubmed/?term=Roselino%20JE%5BAuthor%5D&cauthor=true&cauthor_uid=2629953), [Castro-e-Silva Júnior O](https://www.ncbi.nlm.nih.gov/pubmed/?term=Castro-e-Silva%20J%C3%BAnior%20O%5BAuthor%5D&cauthor=true&cauthor_uid=2629953), [Romanello LM](https://www.ncbi.nlm.nih.gov/pubmed/?term=Romanello%20LM%5BAuthor%5D&cauthor=true&cauthor_uid=2629953) et al (1989) Effect of chlorpromazine and biliary drainage on portal blood flow and mitochondrial function during extrahepatic cholestasis. [*Braz J Med Biol Res*.](https://www.ncbi.nlm.nih.gov/pubmed/?term=2629953) 22: 889-93. |

| **Corticosteroids** |
| --- |
| **Mode of action** |
| 1. Transcriptional activation (anti-inflammatory, ↑gluconeogenesis) 2. Transcriptional repression (mRNA binding) 3. Non-genomic 4. Directly induce apoptosis presumably by inhibition of Bcl-2, the central regulator protein of apoptosis, located at the outer mitochondrial membrane |
| **Theoretical effects on mitochondrial function** |
| Reduces transmembrane mitochondrial potential |
| **Known effects on mitochondrial function** |
| Unknown |
| **Effects measured/seen in mitochondrial patients, mitochondrial cell lines or normal cell lines** |
| Fatal metabolic acidosis, hyperglycemia, and coma after steroid therapy for Kearns-Sayre syndrome, 2 patients (Curless et al 1986)  Steroidal danger in KSS (Feinsmith et al 1988)  Hyperglycemic acidotic coma and death in Kearns-Sayre syndrome (Bachynski et al 1986)  Adult-onset MELAS presenting as herpes encephalitis (Sharfstein et al 1999)  On the link between Bcl-2 family proteins and glucocorticoidinduced apoptosis (Almawi et al 2004) |
| **References** |
| Almawi WY, Melemedjian OK, Jaoude MM (2004) On the link between Bcl-2 family proteins and glucocorticoidinduced apoptosis. *J Leukoc Biol* 76:7–14.  [Bachynski BN](https://www.ncbi.nlm.nih.gov/pubmed/?term=Bachynski%20BN%5BAuthor%5D&cauthor=true&cauthor_uid=3703509), [Flynn JT](https://www.ncbi.nlm.nih.gov/pubmed/?term=Flynn%20JT%5BAuthor%5D&cauthor=true&cauthor_uid=3703509), [Rodrigues MM](https://www.ncbi.nlm.nih.gov/pubmed/?term=Rodrigues%20MM%5BAuthor%5D&cauthor=true&cauthor_uid=3703509) et al (1986) Hyperglycemic acidotic coma and death in Kearns-Sayre syndrome. [*Ophthalmology*.](https://www.ncbi.nlm.nih.gov/pubmed/3703509) 93: 391-6.  [Curless RG](https://www.ncbi.nlm.nih.gov/pubmed/?term=Curless%20RG%5BAuthor%5D&cauthor=true&cauthor_uid=3703301), [Flynn J](https://www.ncbi.nlm.nih.gov/pubmed/?term=Flynn%20J%5BAuthor%5D&cauthor=true&cauthor_uid=3703301), [Bachynski B](https://www.ncbi.nlm.nih.gov/pubmed/?term=Bachynski%20B%5BAuthor%5D&cauthor=true&cauthor_uid=3703301) et al (1986) Fatal metabolic acidosis, hyperglycemia, and coma after steroid therapy for Kearns-Sayre syndrome. [*Neurology*.](https://www.ncbi.nlm.nih.gov/pubmed/3703301) 36: 872-3.  [Feinsmith BM](https://www.ncbi.nlm.nih.gov/pubmed/?term=Feinsmith%20BM%5BAuthor%5D&cauthor=true&cauthor_uid=3050623), [Liebesman WP](https://www.ncbi.nlm.nih.gov/pubmed/?term=Liebesman%20WP%5BAuthor%5D&cauthor=true&cauthor_uid=3050623), [Guibor P](https://www.ncbi.nlm.nih.gov/pubmed/?term=Guibor%20P%5BAuthor%5D&cauthor=true&cauthor_uid=3050623) (1988) Steroid danger in Kearns-Sayre syndrome (KSS). [*N J Med*.](https://www.ncbi.nlm.nih.gov/pubmed/3050623) 85: 659-63.  [Sharfstein SR](https://www.ncbi.nlm.nih.gov/pubmed/?term=Sharfstein%20SR%5BAuthor%5D&cauthor=true&cauthor_uid=10025431), [Gordon MF](https://www.ncbi.nlm.nih.gov/pubmed/?term=Gordon%20MF%5BAuthor%5D&cauthor=true&cauthor_uid=10025431), [Libman RB](https://www.ncbi.nlm.nih.gov/pubmed/?term=Libman%20RB%5BAuthor%5D&cauthor=true&cauthor_uid=10025431) et al (1999) Adult-onset MELAS presenting as herpes encephalitis. [*Arch Neurol.*](https://www.ncbi.nlm.nih.gov/pubmed/10025431) 56: 241-3. |

| **Enalapril** |
| --- |
| **Mode of action** |
| Enalapril is indicated for the treatment of hypertension and heart failure. It is metabolised to enalaprilat which then inhibits angiotensin converting enzyme, decreasing production of Angiotensin II (a potent vasoconstrictor) and decreased aldosterone secretion. Enalaprilat does not cross the blood-brain barrier. |
| **Theoretical effects on mitochondrial function** |
| Increases mitochondrial biogenesis in aging mice and has a positive effect on NADH and cytochrome c oxidoreductase activity (Ferder et al 1993, Piotrkowski et al 2009)  Also thought to increase mitochondrial nitric oxide synthase activity in heart and liver (Boveris et al 2003) |
| **Effects measured/seen in mitochondrial patients, mitochondrial cell lines or normal cell lines** |
| Mouse and rat studies ((Ferder et al 1993, Piotrkowski et al 2009, Boveris et al 2003)  **Supplementary Information**  Side effects*  *Common or very common*  Asthenia; blurred vision; depression; dyspnoea  *Uncommon*  Alopecia; anorexia; arrhythmias; confusion; drowsiness; dry mouth; flushing; hyponatraemia; ileus; impotence; insomnia; muscle cramps; nervousness; palpitation; peptic ulcer; sweating; tinnitus; vertigo  *Rare*  Abnormal dreams; allergic alveolitis; exfoliative dermatitis; glossitis; gynaecomastia; pemphigus; pulmonary infiltrates; Raynaud’s syndrome; Stevens-Johnson syndrome; stomatitis; toxic epidermal necrolysis  *Very rare*  Gastro-intestinal angioedema  *Underlined side effects *may* be clinical considerations in using Enalapril in mitochondrial disease patients. |
| **References** |
| Boveris A, D'Amico G, Lores-Arnaiz S et al (2003) Enalapril increases mitochondrial nitric oxide synthase activity in heart and liver. *Antioxid Redox Signal*. 5: 691–7.  Ferder L, Inserra F, Romano L et al (1993) [Effects of angiotensin-converting enzyme inhibition on mitochondrial number in the aging mouse.](https://www.ncbi.nlm.nih.gov/pubmed/8338123) *Am J Physiol*. 265: C15-8.  Piotrkowski B, Koch OR, De Cavanagh EM et al (2009) [Cardiac mitochondrial function and tissue remodelling are improved by a non-antihypertensive dose of enalapril in spontaneously hypertensive rats.](https://www.ncbi.nlm.nih.gov/pubmed/19296328) *Free Radic Res*. 43: 390-9. |

| **Fentanyl** |
| --- |
| **Mode of action** |
| Primarily a mu-opioid agonist. Fentanyl is also used as an adjunct to general anaesthetics, and as an anaesthetic for induction and maintenance. |
| **Theoretical effects on mitochondrial function** |
| \| **Type of Study** \| **Model Used** \| **Effect Measured** \| **Effect seen/ Mechanism** \| **Year** \| **Ref** \| \| --- \| --- \| --- \| --- \| --- \| --- \| \| **In vitro** \| Human hepatoma HepG2 cells \| Fentanyl ***marginally*** reduced maximal mitochondrial complex–specific respiration rates using exogenous substrates (decrease in medians: 11%–18%; P = 0.003–0.001) but did not affect basal cellular respiration rates (P = 0.834); effect on stimulated respiration was prevented by preincubation with naloxone \| data suggest that fentanyl reduces stimulated mitochondrial respiration of cultured human hepatocytes by a mechanism that is blocked by a mitoKATP channel antagonist \| **2016** \| **1** \| |
| **Effects measured/seen in mitochondrial patients, mitochondrial cell lines or normal cell lines** |
| **Summary** – dozens (possibly hundreds) of patients with mitochondrial disease (children and adults, including Leigh syndrome, KSS, MELAS, other mitochondrial myopathies and encephalomyopathies) have been reported to receive fentanyl anaesthesia without adverse effects. There are a few reports of adverse effects in patients who received multiple agents including fentanyl, eg one report of a short episode of bradycardia in one patient with KSS, and apnoea in one young adult with mitochondrial encephalomyopathy. One infant with congenital lactic acidosis had progressive acidosis and died 2 weeks later, it is difficult to attribute this directly to fentanyl.   \| **Type of Study** \| **Model Used** \| **Effect Measured** \| **Effect seen/ Mechanism** \| **Year** \| **Ref** \| \| --- \| --- \| --- \| --- \| --- \| --- \| \| **In vivo** \| Patient with mito myopathy \| Anaesthesia induced with TPS and fentanyl, and maintained with isoflurane (procedure: hemithroidectomy) \| quick recovery without complications \| **1991**  Italian  (English abstract) \| **2** \| \| **In vivo** \| Patient with KSS \| Propofol-fentanyl anaesthesia \| short episode of bradycardia (heart rate < 50.min-1) \| **1993** German (English abstract) \| **3** \| \| **In vivo** \| 41y woman with KSS \| Anesthesia was induced with incremental doses of fentanyl to 1 mg and midazolam to 8 mg for aortic valve repair, aortic coarctation repair,  and patent ductus arteriosus (PDA) ligatlon; Anesthesia was maintained with fentanyl to a total of 4 mg, midazolam to a total of 15 rag, and intermittent lSOflurane \| authors suggest there is a normal response to fentanyl and midazolam in KSS patients. \| **1995** \| **4** \| \| **In vivo** \| Two patients with MELAS (both 11y girls) \| VO shunt insertion: Anesthesia was induced with fentanyl and midazolam, and vecuronium was used to facilitate tracheal intubation. Volatile anesthetic was avoided, and anesthesia was maintained with fentanyl, midazolam, and nitrous oxide. \| No complications occurred during anesthesia in both patients. \| **1995** Japanese (English abstract) \| **5** \| \| **In vivo** \| 27y female KSS \| MV surgery: Anesthetic induction was achieved with propofol and fentanyl, and maintenance was with nitrous oxide and oxygen through a face mask. \| No adverse events \| **1996** Spanish (English abstract) \| **6** \| \| **In vivo** \| 6y boy with MELAS \| ENT surgery was performed without complications under general anaesthesia using propofol, fentanyl, and ventilation with nitrous oxide and oxygen \| postoperative course was uneventful; Signs or symptoms of malignant hyperthermia never occurred \| **2001**  German (English abstract) \| **7** \| \| **In vivo** \| 49-year-old female with mitochondrial encephalomyopathy \| Cochlear implantation: Anesthesia was induced with 5 mg.kg-1 of propofol, and the trachea was intubated without a muscle relaxant, anesthesia was maintained with a continuous infusion of 4-8 mg.kg-1.hr-1 of propofol, a bolus injection of 50-100 micrograms of fentanyl, and nitrous oxide (66%) in oxygen (33%) \| No cardiovascular instabilities or increase in plasma lactate concentration were observed during surgery. The patient had a smooth recovery from the propofol anesthesia \| **2002** Japanese (English abstract) \| **8** \| \| **In vivo** \| 57y female mito myopathy \| Laparoscopic cholecystectomy for cholelithiasis; total intravenous anesthesia with propofol, fentanyl, and ketamine \| No serious acidosis, hyperlactemia, hypothermia, nor prolonged recovery from the anesthesia was observed \| **2004** Japanese (English abstract) \| **9** \| \| **In vivo** \| 21y male mito encephalomyopathy \| Anaesthesia for removal of maxillary cyst. Induction with intravenous administration of fentanyl 100 microgram \| During the induction of anesthesia, the patient fell into the state of apnea after intravenous administration of fentanyl 100 microgram. The respiratory depression lasted for about 120 minutes after administration of fentanyl, and was antagonized by naloxone 40 microgram. This case suggests that careful administration of fentanyl is mandatory in a patient with mitochondrial encephalomyopathy. \| **2006**  Japanese (English abstract) \| **10** \| \| **In vivo** \| **15y girl** with mitochondrial encephalomyopathy \| tracheostomy under total intravenous anesthesia - Anesthesia was induced with propofol (30 mg) and fentanyl (50 microg), anesthesia was maintained with a continuous infusion of propofol 4-10 mg x kg(-1) x hr(-1) and a bolus injection of fentanyl 25 microg \| patient showed smooth recovery from anesthesia \| **2006**  Japanese (English abstract) \| **11** \| \| **In vivo** \| 53-year-old man with MELAS \| Gastrectomy - After  pre-oxygenation, the patient was given 50  ug fentanyl and 30 mg propofol and was paralyzed with 3 mg  vecuronium. \| No anaesthetic complications reported \| **2007** \| **12** \| \| **In vivo** \| 11y boy with complex III deficiency and encephalomyopathy \| Surgery for ischiotibial muscle spasticity. Midazolam, fentanyl, and propofol were used for anesthetic induction; mivacurium was used during intubation. Anesthetic maintenance was with propofol in continuous infusion and fractionated doses of fentanyl and mivacurium on demand in a mixture of oxygen and air. \| no problems during the anesthetic procedure \| **2006**  Spanish (English abstract) \| **13** \| \| **In vivo** \| 25y woman with Friedreich ataxia \| Caesarean section - peridural anaesthesia with 8 ml 0.75% ropivacaine and 10 microg sufentanil \| no exacerbation of neurological symptoms \| **2009** German (English abstract) \| **14** \| \| **In vivo** \| 58y woman with MELAS \| emergency laparotomy - Anesthesia was induced by rapid-sequence fashion and maintained using midazolam, propofol, ketamine, fentanyl and vecuronium \| uneventful peri-operative course \| **2010**  Japanese (English abstract) \| **15** \| \| **In vivo** \| 37y woman with recurrent exercise-induced rhabdomyolysis caused by m.4281A>G \| fentanyl citrate transdermal patch \| enabled her to perform moderate exercise without pain \| **2011** \| **16** \| \| **In vivo** \| 26y woman with MNGIE \| emergency surgery for megacolon - Rapid sequence induction was accomplished with midazolam, fentanyl, propofol, and rocuronium as an alternative to succinylcholine. Anesthesia was maintained with intravenous propofol \| No intraoperative problems developed and extubation was possible 2 hours after arrival in the postoperative critical care unit \| **2011**  Spanish (English abstract) \| **17** \| \| **In vivo** \| 4 week infant with myopathy and lactic acidosis \| Muscle biopsy - anesthesia was induced with 5 mg propofol, 5 μg fentanyl, and 3 mg rocuronium. A propofol infusion at 200 μg·kg−1·min−1 was used to maintain anesthesia (44 mg propofol total) \| remained intubated and ventilated with a respiratory and metabolic acidosis – worsening lactic acidosis, died 2 weeks later \| **2013** \| **18** \| \| **In vivo** \| 27y woman with mito myopathy and complex III deficiency \| combination of fentanyl (100µg), ketamine, and methohexital for induction, as well as methohexital, sufentanil, and dexmedetomidine for the maintenance of general anesthesia during 2 consecutive surgeries (laparoscopic resection of adenocarcinoma of sigmoid, and resection of lung met) \| uneventful postoperative course \| **2017** \| **19** \| \| **In vivo** \| 8y child with ETFDH deficiency \| surgery for acute appendicitis - combination of fentanyl (50 mcg), low dose propofol and nitrous oxide \| patient remained completely stable \| **2017** \| **20** \| \| **In vivo** \| 877 paediatric patients with neuromusc dis (367 metabolic/ mitochondrial myopathy) – retrospective 20y review \| Anaesthesia for muscle biopsy - Intravenous agents were used in 836 (95.3%) cases with the three commonest agents—propofol, midazolam, and fentanyl—accounting for 77.5% of usage \| none of the 877 patients exhibited signs of intraoperative or postoperative anesthesia-induced muscle injury \| **2016** \| **21** \| \| **In vivo** \| 58 procedures in (retrospective review) \| Fentanyl used in induction in 3 procedures and for induction in 24 cases \| 3 adverse events reported (all received fentanyl analgesia but also many other agents) – hypovolaemia, renal impairment, respiratory failure and metabolic acidosis) \| **2008** \| **22** \| \| **In vivo** \| 11y girl with MERRF \| Bilateral clubfoot surgery - General anesthesia was induced with propofol 2 mg/kg and fentanyl 2ug/kg \| No adverse effects \| **2005** \| **23** \| \| **In vivo** \| 21y woman with Leigh syndrome \| posterior spinal fusion - Anesthesia was induced with IV propofol and maintained with intermittent positive pressure ventilation with desflurane in oxygen and air, supplemented with intermittent IV fentanyl \| Aspiration pneumonia, progressive brain stem necrosis. Death day 62 post procedure \| **2003** \| **24** \| \| **In vivo** \| 13m girl with OXPHOS defect \| Cholecystectomy - premedicated with intravenous midazolam (0.1 mg/kg). Anesthesia was induced with thiopental and fentanyl and maintained with isoflurane in nitrous oxide and oxygen. Pancuronium was used for relaxation \| perioperative white matter degeneration and death \| **1997** \| **25** \| \| **In vivo** \| 20y man with MELAS \| Cochlear implant - midazolam premeditation, anesthesia induced with propofol and cisatracurium and maintainedwith propofol and cisatracurium infusions; 50 ug of fentanyl was given for analgesia, \| Anaesthetic recovery was without complication \| **1997** \| **26** \| \| **In vivo** \| 41y woman with KSS \| Aortic valve repair - Anesthesia was induced with incremental doses of fentanyl to 1 mg and midazolam to 8 mg, and maintained with fentanyl to a total of 4 mg, midazolam to a total of 15 mg, and intermittent lSOflurane \| Authors suggest there is a normal response to fentanyl and midazolam in KSS patients. \| **1995** \| **27** \| |
| **References** |
| 1. Djafarzadeh S, Vuda M, Jeger V et al (2016) The Effects of Fentanyl on Hepatic Mitochondrial Function. *Anesth Analg*. 123: 311-25.  2. Perilli V, Sollazzi L, Valenti M et al (1991) [Use of atracurium in patients with muscular diseases. 2 cases of distal and mitochondrial myopathy]. *Minerva Anestesiol*. 57: 441-4. Italian.  3. Klockgether-Radke A, Henze T, Braun U et al (1993) [General anesthesia in two patients with mitochondrial myopathy]. *Anaesthesist.* 42: 111-4. German.  4. Pivalizza EG, Ando KJ, Sweeney MS (1995) Kearns-Sayre syndrome and cardiac anesthesia. *J Cardiothorac Vasc Anesth*. 9: 189-91.  5. Itaya K, Takahata O, Mamiya K et al (1995) [Anesthetic management of two patients with mitochondrial encephalopathy, lactic acidosis and stroke-like episodes (MELAS)]. *Masui.* 44: 710-2. Japanese.  6. Sabaté S, Ferrándiz M, Paniagua P et al (1996) [Anesthesia in Kearns-Sayre syndrome (mitochondrial myopathy)]. *Rev Esp Anestesiol Reanim.* 43: 255-7. Spanish.  7. Thiel A, Ritzka M, Saur G. (2001) [Anesthesia in mitochondrial encephalomyopathies]. *Anasthesiol Intensivmed Notfallmed Schmerzther.* 36: 437-9. German.  8. Shibukawa K, Kawamata M, Seki S et al (2002) [The use of propofol combined with nitrous oxide and fentanyl in anesthetic management of a patient with mitochondrial encephalomyopathy]. *Masui.* 51: 888-91. Japanese.  9. Kushikata T, Yatsu Y, Kubota T et al (2004) [Total intravenous anesthesia with propofol, ketamine, and fentanyl (PFK) for a patient with mitochondrial myopathy]. *Masui.* 53: 178-80. Japanese.  10. Ishiguro M, Hashimoto K, Hayakata Y et al (2006) [Prolonged respiratory depression after fentanyl administration in a patient with mitochondrial encephalomyopathy]. *Masui.* 55: 73-5. Japanese.  11. Tatsumi Y, Nakashima M, Kitao T et al (2006) [Anesthetic management of a patient with mitochondrial encephalomyopathy under total intravenous anesthesia]. *Masui.* 55: 1228-30. Japanese.  12. Sasano N, Fujita Y, So M et al (2007) Anesthetic management of a patient with mitochondrial myopathy, encephalopathy, lactic acidosis, and stroke-like episodes (MELAS) during laparotomy. *J Anesth*. 21: 72-5.  13. Ortiz-Gómez JR, Souto-Ferro JM (2006) [Anesthesia for a patient with mitochondrial respiratory chain complex III deficiency]. *Rev Esp Anestesiol Reanim*. 53: 575-9. Spanish.  14. Hanusch P, Heyn J, Well H et al (2009) [Peridural anaesthesia with ropivacaine for a patient with Friedrich's ataxia. Caesarean section after dorsal stabilisation of the spinal column (Th5-L1)]. *Anaesthesist*. 58: 691-4. German.  15. Imai Y, Kitamura T, Kawamura G et al (2010) [Anesthetic management of emergency total gastrectomy in a patient with mitochondrial encephalomyopathy: a case of gastric perforation accompanied by systemic inflammatory response syndrome]. *Masui.* 59: 765-9. Japanese.  16. Patchett DC, Grover ML (2011) Mitochondrial myopathy presenting as rhabdomyolysis. *J Am Osteopath Assoc*. 111: 404-5.  17. Ibáñez C, Fernández-González I (2011) [Emergency anesthesia in a woman with mitochondrial neurogastrointestinal encephalopathy]. *Rev Esp Anestesiol Reanim*. 58: 585-7. Spanish.  18. Saettele AK, Sharma A, Murray DJ (2013) Case scenario: Hypotonia in infancy: anesthetic dilemma. *Anesthesiology.* 119: 443-6.  19. Woodward EL, Xiong Z (2017) Use of Methohexital and Dexmedetomidine for Maintenance of Anesthesia in a Patient With Mitochondrial Myopathy: A Case Report. *A A Case Rep*. 8: 33-35.  20. Lilitsis E, Astyrakaki E, Blevrakis E (2017) Anesthetic management of a pediatric patient with Electron Transfer Flavoprotein Dehydrogenase deficiency (ETFDH) and acute appendicitis: case report and review of the literature. *BMC Anesthesiol*. 17: 116.  21. Shapiro F, Athiraman U, Clendenin DJ (2016) Anesthetic management of 877 pediatric patients undergoing muscle biopsy for neuromuscular disorders: a 20-year review. *Paediatr Anaesth.* 26: 710-21.  22. Footitt EJ, Sinha MD, Raiman JA (2008) Mitochondrial disorders and general anaesthesia: a case series and review. *Br J Anaesth.* 100: 436-41.  23. Vilela H, Garcìa-Fernández J, Parodi E et al (2005) Anesthetic management of a patient with MERRF syndrome. *Paediatr Anaesth.* 15: 77-9.  24. Cooper MA, Fox R (2003) Anesthesia for corrective spinal surgery in a patient with Leigh's disease. *Anesth Analg*. 97: 1539-41.  25. Casta A, Quackenbush EJ, Houck CS et al (1997) Perioperative white matter degeneration and death in a patient with a defect in mitochondrial oxidative phosphorylation. *Anesthesiology.* 87: 420-5.  26. Thompson VA, Wahr JA (1997) Anesthetic considerations in patients presenting with mitochondrial myopathy, encephalopathy, lactic acidosis, and stroke-like episodes (MELAS) syndrome. *Anesth Analg*. 85: 1404-6.  27. Pivalizza EG, Ando KJ, Sweeney MS (1995) Kearns-Sayre syndrome and cardiac anesthesia. *J Cardiothorac Vasc Anesth.* 9: 189-91. |

| **Fibrate drugs [group] *e.g.* clofibrate, ciprofibrate** |
| --- |
| **Mode of action** |
| Fibrates are agonists of the nuclear transcription factor peroxisome proliferator-activated receptor (PPAR)-α that also activate PPAR-β/δ and PPAR-γ, but with a far lower potency. Their cholesterol- and triglyceride-lowering effect is mediated via PPAR-α (Fruchart & Duriez, 2006), which downregulates apoprotein C-III (an inhibitor of lipoprotein lipase) and upregulates the synthesis of apolipoprotein A-I, fatty acid transport protein, and lipoprotein lipase resulting in an increase in VLDL catabolism, fatty acid oxidation, and elimination of triglyceride-rich particles (Tiwari & Khokhar, 2014). As a result of a decrease in VLDL levels, total plasma triglycerides are reduced by 30 to 60%, and a modest increase in HDL occurs in some hypertriglyceridemic patients (Tiwari & Khokhar, 2014). |
| **Theoretical effects on mitochondrial function** |
| Traditionally, fibrate-induced mitochondrial dysfunction received most attention, as long-term treatment is associated with mitochondrial toxicity linked to inhibition of complex I (Brunmair et al, 2004; Yamada, 2013). Though more recently, bezafibrate, which also inhibits complex I (Yamada, 2013), has been shown to improve mitochondrial function, including acylcarnitine metabolism (Shioya *et al*, 2014; Yamada, 2017) and enzymatic activity of the respiratory complexes (I, III, and IV) in patient-derived myoblasts and fibroblasts of patients with a deficiency of these complexes (Bastin *et al*, 2008). |
| **Known effects on mitochondrial function** |
| Fibrates are known to impair mitochondrial function *in vitro* (Brunmair *et al.*, 2004). Fibrates, like fenofibrate and clofibrate, induce mitochondrial dysfunction via various molecular mechanisms and some of them predominantly inhibit respiratory chain complex I (Brunmair *et al.*, 2004). Ciprofibrate and gemfibrocil, but not bezafibrate, impair cell respiration without complex I inhibition. The myotoxic effect of fibrates may be enhanced by the combination with other drugs. Particularly, combined statin and fibrate therapy can markedly enhance the risk of developing myopathy (Johnson *et al.*,2005). In contrast, studies in patient-derived fibroblasts or myoblasts with a complex I-, II-, or IV-deficiency have shown that bezafibrate can stimulate the activity of these respiratory complexes (Bastin *et al.*, 2008). Fibrates have been also shown to correct carnitine palmitoyl-transferase II deficiency (Laforêt *et al.*, 2007) in patient-derived cells, and clofibrate induces the production of 16S-rRNA in rat liver mitochondria (Cai *et al*., 1996). |
| **Effects measured/seen in mitochondrial patients, mitochondrial cell lines or normal cell lines** |
| The effects of fibrates on patients with mitochondrial diseases mainly relate to the positive effects of these drugs (Avula et al, 2014). In this respect bezafibrate is most studied and has been shown to induce mitochondrial biogenesis and to control the expression of antioxidant enzymes and uncoupling proteins (Avula *et al*, 2014). In a murine model of COX deficiency (Noe *et al*, 2013), bezafibrate administration improved mitochondrial protein production and mitochondrial ATP-generating capacity. Overall, bezafibrate was neuroprotective in this mouse model of mitochondrial encephalopathy, but the paper has been retracted due to scientific integrity issues. The clinical trial of bezafibrate treatment for a boy with the intermediate form of glutaric acidemia type 2 (GA2) showed that daily administration dramatically improved his motor and cognitive skills, accompanied by sustained reduction of C4, C8, C10, and C12 acylcarnitines in blood and normalized urinary organic acid profile (Shioya *et al*, 2014). No major adverse effects have been observed. These findings align with the stimulatory effects of bezafibrate on the fatty acid oxidation observed in fibroblasts from glutaric acidemia type II patients (Yamada *et al*, 2017). This implies that bezafibrate may be a promising therapeutic agent for the treatment of neurodegenerative disease associated with mitochondrial dysfunction, although further study is warranted (Avula *et al*, 2014). Adverse effects of fibrates in mitochondrial patients have not been described, however, caution is warranted. In a case-report fenofibrate in combination with metformin and rosiglitazone was associated with myopathy (Ledl *et al.*, 2005). The authors concluded that fibrates combined with thiazolidinediones may have a myotoxic effect, which necessitates more frequent monitoring of these patients by measurements of serum creatine kinase and creatinine, particularly if there is a preexisting nephropathy or polyneuropathy. |
| **References** |
| Avula S, Parikh S, Demarest S et al (2014) Treatment of mitochondrial disorders. *Curr Treat Opt Neur* 16: 292.  Bastin J, Aubey F, Rotig A et al (2008) Activation of peroxisome proliferator-activated receptor pathway stimulates the mitochondrial respiratory chain and can correct deficiencies in patients’ cells lacking its components. *J Clin Endocrinol Metab* 93: 1433-41.  Brunmair B, Lest A, Staniek K et al (2004) Fenofibrate impairs rat mitochondrial function by inhibition of respiratory complex I. *J Pharmacol Exp Ther* 311: 109-14.  Cai, Y, Nelson BD, Li R et al (1996). Thyromimetic action of the peroxisome proliferators clofibrate, perfluorooctanoic acid, and acetylsalicylic acid includes changes in mRNA levels for certain genes involved in mitochondrial biogenesis. *Arch Biochem Biophys* 325: 107-12.  Fruchart, JC, Duriez P (2006) Mode of action of fibrates in the regulation of triglyceride and HDL-cholesterol metabolism. *Drugs Today* 42: 39-64.  Hamilton-Craig I, Yudi M, Johnson L et al (2012) Fenofibrate therapy in carnitine palmitoyl transferase type 2 deficiency. *Case Rep Med*, 2012.  Johnson, TE, Zhang X, Shi S et al (2005) Statins and PPARα agonists induce myotoxicity in differentiated rat skeletal muscle cultures but do not exhibit synergy with co-treatment. *Toxicol Appl Pharmacol* 208: 210-21.  Laforêt P, Nicolino M, Eymard B (2007) New approaches for the treatment of metabolic myopathies. *Rev neurologiq*, 163; 930-5.  Ledl M, Hohenecker J, Francesconi C et al (2005) Acute myopathy in a type 2 diabetic patient on combination therapy with metformin, fenofibrate and rosiglitazone. *Diabetol* 48: 1996-8.  Noe N, Dillon L, Lellek V et al (2013) RETRACTED: Bezafibrate improves mitochondrial function in the CNS of a mouse model of mitochondrial encephalopathy. *Mitochondrion* 13: 417-26  Shioya A, Takuma H, Yamaguchi S et al (2014) Amelioration of acylcarnitine profile using bezafibrate and riboflavin in a case of adult-onset glutaric acidemia type 2 with novel mutations of the electron transfer flavoprotein dehydrogenase (ETFDH) gene. *J Neurol Sci* 346: 350-2.  Tiwari V, Khokhar M (2014) Mechanism of action of anti-hypercholesterolemia drugs and their resistance. *Eur J Pharmacol*, 741: 156-70.  Yamada, K, Kobayashi H, Bo R et al (2017) Efficacy of bezafibrate on fibroblasts of glutaric acidemia type II patients evaluated using an in vitro probe acylcarnitine assay. *Brain Develop* 39: 48-57.  Yamada K, Tsunoda K, Kawai K et al (2013) Mitochondria toxicity of antihyperlipidemic agents bezafibrate and fenofibrate. *Diabetol Int* 4: 126-31. |

| **Fluphenazine** |
| --- |
| **Mode of action** |
| Is a phenothiazine; antagonizes dopaminergic D1 and D2 receptors; depresses release of hypothalamic and hypophyseal hormones. |
| **Known effects on mitochondrial function** |
| Study published in Annals of Neurology in 1993 (Burkhardt et al 1993) that looked at neuroleptics haloperidol, clozapine etc and showed complex 1 deficiency. The study looked at rat brain mitochondria after treatment with various compounds. There is another early paper looking at phenothiazines that showed there was inhibition of complexes I and IV (Dawkins et al 1959).  Also appears to be evidence that phenothiazines can inhibit succinate oxidation in vitro (old papers).  Other studies support the view that classical neuroleptics such as haloperidol inhibit mitochondrial complex I through oxidative modification of the enzyme complex that can be reversed by concomitant treatment with glutathione (Balijepalli et al 1999). These studies used haloperidol as the main example and showed decrease of complex I by fluphenazine in brain slices but not the GSH effect.  Phenothiazine derivatives induced the mitochondrial permeability transition associated with cytochrome c release. All phenothiazine derivatives decreased the viability of the HTC cells in a concentration-dependent manner and exhibited different cytotoxic potencies. The EC_50_ values ranged from 45 to 125 μM. They also looked at the membrane potential using rhodamine 123 and concentrations of PTZ = 50 µM. |
| **Effects measured/seen in mitochondrial patients, mitochondrial cell lines or normal cell lines** |
| Apart from studies that look at the whole group, and include haloperidol, the evidence for mitochondrial toxicity of fluphenazine (and phenothiazines as a whole) is poor. There is some evidence that they are anti-tumour albeit with lower mitochondrial membrane potential (Zhang et al 2017). There is also some evidence that they are neuroprotective. |
| **References** |
| Balijepalli S, Boyd MR, Ravindranath V (1999) Inhibition of mitochondrial complex I by haloperidol: the role of thiol oxidation. *Neuropharmacology* 38: 567-77.  Burkhardt C, Kelly JP, Lim YH et al (1993) Neuroleptic medications inhibit complex I of the electron transport chain. *Ann Neurol* 33: 512-17.  Dawkins MJR, Judah JD, Rees KR (1959) Action of Some Phenothiazine Derivatives on the Respiratory Chain. *Biochemical Pharmacology* 2: 112-20.  Mavroidis ML, Kanter DR, Hirschowitz J et al (1984) Fluphenazine plasma levels and clinical response. *J Clin Psychiatry* 45: 370-3.  Zhang C, Gong P, Liu P et al (2017) Thioridazine elicits potent antitumor effects in colorectal cancer stem cells. *Oncol Rep* 37: 1168-74. |

| **Gabapentin** |
| --- |
| **Mode of action** |
| Gabapentin is structurally related to GABA. However, the drug does not bind to GABA_A_ or GABA_B_ receptors, and it does not appear to influence synthesis or uptake of GABA. High affinity gabapentin binding sites have been located throughout the brain; these sites correspond to the presence of voltage-gated calcium channels specifically possessing the alpha-2-delta-1 subunit. This channel appears to be located presynaptically and may modulate the release of excitatory neurotransmitters which participate in epileptogenesis and nociception. |
| **Theoretical effects on mitochondrial function** |
| There are indications that gabapentin (GBT) inhibits the mitochondrial branched-chain aminotransferase, which reversibly catalyzes transamination of essential branched-chain amino acids (Hutson *et al*, 2001) although this finding has been challenged (Goto *et al*, 2005). |
| **Known effects on mitochondrial function** |
| Whether GBT inhibits the mitochondrial branched-chain aminotransferase, which reversibly catalyses transamination of the essential branched-chain amino acids leucine, isoleucine, and valin, remains a matter of debate (Goto *et al*, 2005; Hutson *et al*, 2001).  In contrast, in rats exposed to 3-nitropropionic acid, which reduces oxidative defense and impairs function of complexes I, II, and IV of the respiratory chain, administration of GBT restored mitochondrial oxidative defense mechanisms and respiratory chain complex activities (Kumar *et al*, 2012). In addition, GBT-lactam, a GBT analogue, has been shown to exhibit neuroprotective effects by opening mitochondrial ATP-dependent K-channels in a transgenic mouse model of Huntington’s Disease (Zucker *et al*, 2004). However, in this study gabapentin itself did not gave similar beneficial effects. Remarkably, these beneficial effects have been assigned to the above adverse mechanism in which GBT has been shown to inhibit mitochondrial forms of the brain branched-chain aminotransferase, as in rats it resulted in reduced synthesis of glutamate from branched chain amino acids (Goldlust *et al*, 1995). Furthermore, in another animal study gabapentin protected against rotenone-induced striatal neuronal dysfunction (Costa *et al*, 2008). |
| **Effects measured/seen in mitochondrial patients, mitochondrial cell lines or normal cell lines** |
| Beneficial effects of GBT in patients with a mitochondrial disorder have been described, which supports its use for the relief of neuropathic pain in these patients (Hirano, 2016; Ahaja, 2018). |
| **References** |
| Costa C, Belcastro V, Tozzi A et al (2008) Electrophysiology and pharmacology of striatal neuronal dysfunction induced by mitochondrial complex I inhibition. *J Neurosci*, 28: 8040-52.  Goldlust A, Su TZ, Welty DF et al (1995) Effects of anticonvulsant drug gabapentin on the enzymes in metabolic pathways of glutamate and GABA. *Epilepsy Res* 22: 1-11.  Goto M, Miyahara I, Hirotsu K (2005) Structural determinants for branched-chain aminotransferase isozyme specific inhibition by the anticonvulsant drug gabapentin. *J Biol Chem* 280: 37246-56.  Hutson SM, Lieth E, LaNoue KF (2001) Function of leucine in excitatory neurotransmitter metabolism in the central nervous system. *J Nutr* 131: 846S-50S.  Kumar P, Kalonia H, Kumar A (2012) Possible GABAergic mechanism in the neuroprotective effect of gabapentin and lamotrigine against 3-nitropropionic acid induced neurotoxicity. *Eur J Pharmacol* 674: 265-74.  Zucker B, Ludin DE, Gerds TA (2004) Gabapentin-lactam, but not gabapentin, reduces protein aggregates and improves motor performance in a transgenic mouse model of Huntington’s disease. *Naunyn-Schmiedebergs Arch Pharmacol*, 370: 131-9. |

| **Glitazones** |
| --- |
| **Mode of action** |
| Glitazones are antidiabetic, anti-inflammatory drugs which are PPAR gamma agonists (1) whose intended therapeutic action is to increase insulin sensitivity in Type 2 Diabetes Mellitus (2). They also modulate hepatic glucocse transport and improve glucose utilization in skeletal muscle and cause preadipocyte differentiation. (3)  Known pattern of toxicity - Tro/Pio/Rosi  Troglitazone was removed from the market in 2000 due to severe acute hepatic necrosis in a minority of patients (4, 5, 6). This includes including elevated liver transaminases, acute liver failure/necrosis. The mechanism of effect is thought to be PPAR gamma independent. Proposed mechanisms include direct cytotoxicity, cholestasis through inhibition of bile acid transporters, mitochondrial dysfunction (2) formation of reactive quinone metabolites (7). Pioglitazone and Rosiglitazone have also caused acute liver failure in patients but this is extremely rare (5). |
| **Theoretical effects on mitochondrial function** |
| Theoretical effect on mitochondrial function  Toxic effects:   1. Reduced mitochondrial membrane potential (MMP) (2,7,8,9, 10,11, 12, 13) 2. Mitochondrial permeability transition (MPT) (14, 15,16, 17) 3. OXPHOS complex inhibition (1,8,18, 19, 20) 4. ROS generation/ reduced glutathione (8 ,9, 10, 12, 17, 18, 20, 21, 22) 5. Induction of apoptosis (2,9, 10,14,9,22, 23) 6. Abnormal mito morphology (8, 12, 13, 15,16,17, 24) 7. Reduced respiration/ATP (1,7, 8, 13, 16, 18, 19, 25) 8. Reduced mtDNA content/mtDNA damage (8, 23)   Protective effects   1. Improved mitochondrial biogenesis (26,27) 2. Improved mitochondrial viability (28) 3. Improved OXPHOS function (27,28, 29,30) 4. Reduced mtDNA deletions/ increased mtDNA content (27,29) 5. Improved mito morphology (29) |
| **Known effects on mitochondrial function** |
| Toxic effects   \| Type of Study \| Model Used \| Effect Measured \| Effect seen/ Mechanism \| Ref \| \| --- \| --- \| --- \| --- \| --- \| \| In vitro \| HepG2/C3a cell line  (human hepatoma cells)  Tro exposure \| Oxygen uptake  OXPHOS activity \| O2 uptake reduced to 8% of normal at highest [Tro], TC50 was 285mcM. Activity of effect is 40-100mins and is dose dependent.  OXPHOS activity reduced. Compensatory increase in glycolytic ATP production \| 1 \| \| In vitro \| HepG2 cells  (human hepatoma cells)  Tro exposure \| MMP  Ca fluxes  Caspase 3  ATP levels \| Drop in MMP potential at 100mcM. This completed after 5mins and resulted in rises in Ca levels and activation of caspase 3 leading to apoptosis. No change to ATP levels, possibly due to increased glycolysis \| 2 \| \| In vitro \| Rat and human hepatocytes  Human lymphocytes  Tro, Pio, Rosi exposure \| LDH, LFTs, reductive metabolism (measured as MTT conversion), MMP, ATP levels \| In rats: For both Tro and Rosi: Increased LDH, ALT, AST, deduced reductive metabolism, reduced MMP, membrane permeability and loss of plasma membrane integrity, reduced ATP levels.  In humans: MMP dropped in one patients’s cells but not the other. Lymphocyte MMP dropped on exposure to Tro, Pio, Rosi \| 7 \| \| In vitro \| HepaRG (human hepatoma cells)  Tro, Pio, Rosi exposure \| O2 consumption  OXPHOS activity  ROS, ATP, MMP  Mito morphology  mtDNA content \| For all of the three drugs: O2 consumption rate decreased, decreased OXPHOS activity (CI to C IV), ATP reduced, ROS increased. mtDNA content reduced, MMP reduced, abnormal mito structure seen (significant swelling and ruptured cristae). \| 8 \| \| In vitro \| Opposum kidney epithelial cells  Cig exposure \| ROS, Ca flux, cell viability \| Increased ROS production, and a transient increase in Ca, via a SOCC channel, triggered MAPK and disrupted mito membrane potential which further results in the nuclear translocation of A1F inducing apoptosis. \| 9 \| \| In vitro \| HC 04 immortalised human hepatocytes  Tro exposure \| MMP, ROS, Ask1 levels \| Tro causes a conc.t and time dependent apoptosis after 12-24h, dissipates the MMP., increases levels of superoxides, activates Trx2 Ask1 pathway leading to apoptosis. \| 10 \| \| In vivo \| Zebrafish  Troa nd Pio exposure \| MMP measured live using ZMJ214 dye \| Decreased MMP for Tro but not Pio at 80mcM infusion \| 11 \| \| In vitro \| OUMS 29 Human hepatocytes  Tro, Pio, Cig exposure \| Flow cytometry to assess mito volume, MMP, ROS \| Increased mitochondrial volume on flow cytometry, reduced MMP, increased ROS especially hydrogen peroxide. \| 12 \| \| In vitro \| HepG2 cell line  Tro exposure \| Cell viability, LDH release, ATP, MMP, mito morphology \| Tro caused a conc and time depenedent increase in cell death as measured by LDH release. Reduced ATP production, reduced MMP.  Abnormal mito morphology: invaginated mito, duplicated double membranes and reticulated cristae.  [Tro] of 50-100mcM caused total loss of cell viability within 5h. \| 13 \| \| In vitro \| HepG2 cells  Tro exposure \| MPT,Ca flux  Caspase 3 induction \| Use of 400mcM of Tro caused mit perm transition within 10mins, intracellular Ca increased from 60mins, caspase 3/apoptosis induction from 30mins \| 14 \| \| In vitro \| Isolated mouse liver mitochondria  Trp, Cig, Rosi, Pio exposure \| Mito morphology, Ca flux, MPT \| For tro and cig, increased mito swelling, increased intramitochondrial Ca accumulation and increased mitochondrial permeability transition (MPT).  These effects were not seen Rosi or Pio \| 15 \| \| In vitro \| Isolated rat liver mitochondria  Tro exposure \| MPT, O2 consumption, mito morphology \| Reduced O2 consumption in stage 3 respiration, opening of different MTPs depending on [Tro]. Induction of mitochondrial swelling \| 16 \| \| In vitro \| Rat Diabetic Model (ZDF+/+). Isolated liver mitochondria, hepatocytes  Tro exposure \| Mito morphology, MPT, glutathione levels, MMP, OXPHOS (respiration) \| MPT was induced moreso in diabetic rats than WT rats. Reduced glutathione in liver mitochondria of DM rats on tro exposure. Mito swelling noted.OXPHOS and MMP unaffected. \| 17 \| \| In vivo  In vitro \| Perfused working mouse heart, isolated cardiomyocyte mitochondria  Rosi exposure \| Cardiac function, in vivo and in vitro ATP levels  CI/CIV activity  ROS levels \| Supratherapeutic [Rosi] caused myocardial energy deficiency - reduced ATP, ATP/ADP ratio, PCr, respiratiion, reduced CI and CIV activity and increased ROS production. \| 18 \| \| In vitro \| Rat isolated liver mitochondria  Multiple glitazone exposure \| O2 consumption, OXPHOS activity \| Disruption o O2 consumption and potent inhibtion of CII,II,IV,V  the rank order of potency of inbibtion of respiration is tro=cig= dar>rosi>mur>pio in State 2 respiration. For OXPHOS inhibition the rank order is tro>ciglitazone> dar> mur>rosi >pio.  IC50 values for all of these effects are provided. \| 19 \| \| In vivo \| SOD -/+ mice  Isolated hepatocyte mitochondria  Tro exposure \| ALT, liver histology, CI activity, ROS. \| High dose intraperitoneal Tro of 30mg/kg/d for 4 weeks caused deranged ALT, hepatic necrosis in SOD -/+ mice but not WT mice, Hepatic mito showed reduced CI, increased ROS \| 20 \| \| In vivo \| SOD2 -/+ mice  Isolated hepatic mitohchondria  Tro exposure \| Mitoproteome  ROS  Liver histology  Serum ALT \| Damage of mitochondrial proteins involved in cell death, fatty acid metabolism, mitochondrial glutathiomne import. In addition liver histology shows delayed onset liver injury similar to humans – confluent areas of cytoplasmic vacuolation and hepatocyte death, serum ALT was elevated also. \| 21 \| \| In vitro \| HepG2 cells  Tro exposure \| PGC1a expression, ROS, cell, mito mass \| PGC1 alpha expression is reduced, ROS increased and apotosis increased on exposure of cells to T. Overexpression of this factor restores mito mass and therefore shows that T may caused cytotoxicity through this mechanism \| 22 \| \| In vitro \| Human primary hepatocytes  Tro, rosi exposure \| Cell viability, cytc release, ATP, intact mtDNA levels \| There is apoptosis and cytc release and reduced ATP production, increased mtDNA damage. These are seen in [Tro] of 5-50mcM but not in Rosi. \| 23 \| \| In vitro \| Rat isolated liver mitochondria  Pio exposure \| Mito viability(MTT assay), ATP, SOD activity, Glutathione levels  Mito morphology \| Reduced mito viability with an LC50 of 880mcM. Reduced ATP production and SOD activity in mito, increased mito swelling, no reduction in glutathione. \| 24 \| \| In vitro \| Primary human hepatocytes  Tro exposure \| O2 consumption  Caspase 3/7 activity \| Reduced O2 consumption rate and no effect on caspase 3/7 activity –i.e. no apoptosis thereby contradicts (2) \| 25 \| \| In vivo \| SOD2 knock out mice  Tro exposure \| LFTs, liver histology, mass \| Slight increase in liver mass, ALT, AST, ALP with T in both SOD KO and WT groups. No liver necrosis seen but centrilobular hepatocyte hypertrophy was seen. Suggest that this is not due to mito toxicity as the effect seen in SOD2 knock out animals was mild. \| 31 \| \| In vitro \| Rat heart and liver homogenate  Tro, Pio, Rosi exposure \| Binding affinity (proteomic approach) \| All three of Tro, Pio, Rosi have affinity to several mitochondrial enzymes as determined by proteomic approaches, including C V and TCA cycle proteins. \| 32 \|   Protective effects   \| In vivo \| Rats  Rosi exposure \| O2 consumption \| Rats exposed to nicotine antenatally have reduced O2 consumption that is rescued with postnatal Rosi administration \| 26 \| \| --- \| --- \| --- \| --- \| --- \| \| In vitro \| SH-SY5Y human neuroblatoma cell line  Pio, Rosi exposure \| mtDNA, PGC1a NRF1, TFAM, CO1 and COIV protein expression. \| Pio and Rosi rescued P and R induced cell loss at nanomolar concentrations. They stimulated mitochondrial biogenesis, increased mtDNA levels and increased PGC1 alpha, NRF1, TFAM, CO1 and COIV protein expression. \| 27 \| \| In vitro \| HIT-T15 cells  Pio exposure \| ATP, cell viability, PGC1a and NRF1 mRNA transcripts \| Pio rescued palmitate induced apoptosis, reduced ATP/ADP ratio, enhanced expression of PGC1 and NRF1 mRNA transcripts (which are controllers of mito OXPHOS subunit gene transcription) \| 28 \| \| In vivo \| Rats  Rosi exposure \| mtDNA deletions  Complex IV activity  Mito morphology \| Rosi improved mito dysfunction/ structural abnormalities induced by in utero nicotine exposed mice, in pancreatic cells of DM prone rats, as well as restoring mito CIV and preventing mtDNA deletions in beta cells of the rats. \| 29 \| \| In vitro \| SH – SY5Y human neuroblatoma cell line  Rosi exposure \| MMP, ATP, Caspase 9, CI activity, cyt c \| Rosi rescued cells from negative effects of Delmethrin, namely cyt c release, activation of caspase 9, CI inhibition, reduced MMP, ATP levels. The [Rosi] was 10mcM, a much lower concentration than that which causes hepatoxicity in vitro. \| 30 \| |
| **Effects measured/seen in mitochondrial patients, mitochondrial cell lines or normal cell lines** |
| Troglitazone has been removed from the market due to idiosyncratic hepatotoxicity. It is hypothesized that its toxicity may be induced in genetically susceptible individuals (2, 33) including individuals with mutations in genes controlling mitochondrial function leading to a state of elevated baseline oxidative stress. Of the glitazones listed, only pioglitazone and rosiglitazone remain prescribable. Numerous experiments in vitro using supratherapeutic concentrations of glitazones(33) has been able to induce mitochondrial toxicity via the aforementioned mechanisms. In vivo studies in wild type mice have been unable to induce liver failure, while SOD -/+ mice have demonstrated hepatic necrosis on Tro exposure in one study(20) but not in another(21). However, no studies have investigated toxicity in patients (or cell lines of patients) with confirmed mitochondrial disorders. In addition, the data suggest conflicting protective effects on mitochondrial function for Pio and Rosi at low concentrations when rodents are exposed to known mitotoxic compounds/conditions. |
| **References** |
| 1. Bavli D, Prill S, Ezra E et al (2016) Real-time monitoring of metabolic function in liver-on-chip microdevices tracks the dynamics of mitochondrial dysfunction. *Proc Natl Acad Sci U S A* 113: E2231-40. 2. Bova MP, Tam D, McMahon G et al (2005) Troglitazone induces a rapid drop of mitochondrial membrane potential in liver HepG2 cells. *Toxicol Lett* 155: 41-50. 3. Lehmann JM, Moore LB, Smith-Oliver TA et al (1995) An antidiabetic thiazolidinedione is a high affinity ligand for peroxisome proliferator-activated receptor gamma (PPAR gamma). *J Biol Chem* 270:12953-6. 4. Graham DJ, Green L, Senior JR et al (2003) Troglitazone-induced liver failure: a case study. *Am J Med* 114: 299-306. 5. Julie NL, Julie IM, Kende Al et al (2008) Mitochondrial dysfunction and delayed hepatotoxicity: another lesson from troglitazone. *Diabetologia* 51: 2108-16. 6. Watkins PB, Whitcomb RW (1998) Hepatic dysfunction associated with troglitazone. *N Engl J Med* 338: 916-7. 7. Haskins JR, Rowse P, Rahbari R (2001) Thiazolidinedione toxicity to isolated hepatocytes revealed by coherent multiprobe fluorescence microscopy and correlated with multiparameter flow cytometry of peripheral leukocytes. *Arch Toxicol* 75: 425-38. 8. Hu D, Wu CQ, Li ZJ et al (2015) Characterizing the mechanism of thiazolidinedione-induced hepatotoxicity: An in vitro model in mitochondria*. Toxicol Appl Pharmacol* 284: 134-41. 9. Kwon CH, Park JY, Kim TH et al (2009) Ciglitazone induces apoptosis via activation of p38 MAPK and AIF nuclear translocation mediated by reactive oxygen species and Ca(2+) in opossum kidney cells. *Toxicology* 257: 1-9. 10. Lim PL, Liu J, Go ML et al (2008) The mitochondrial superoxide/thioredoxin-2/Ask1 signaling pathway is critically involved in troglitazone-induced cell injury to human hepatocytes. *Toxicol Sci* 101: 341-9. 11. Sasagawa S, Nishimura Y, Koiwa J et al (2016) In Vivo Detection of Mitochondrial Dysfunction Induced by Clinical Drugs and Disease-Associated Genes Using a Novel Dye ZMJ214 in Zebrafish. *ACS Chem Biol* 11: 381-8. 12. Shishido S, Koga H, Harada M et al (2003) Hydrogen peroxide overproduction in megamitochondria of troglitazone-treated human hepatocytes. *Hepatology* 37: 136-47. 13. Tirmenstein MA, Hu CX, Gales TL et al (2002) Effects of troglitazone on HepG2 viability and mitochondrial function. Toxicol Sci 69: 131-8. 14. Kim JA, Han E, Eun CJ et al (2012) Real-time concurrent monitoring of apoptosis, cytosolic calcium, and mitochondria permeability transition for hypermulticolor high-content screening of drug-induced mitochondrial dysfunction-mediated hepatotoxicity. *Toxicol Lett* 214: 175-81. 15. Masubuchi Y, Kano S, Horie T (2006) Mitochondrial permeability transition as a potential determinant of hepatotoxicity of antidiabetic thiazolidinediones. *Toxicology* 222: 233-9. 16. Okuda T, Norioka M, Shitara Y et al (2010) Multiple mechanisms underlying troglitazone-induced mitochondrial permeability transition. *Toxicol Appl Pharmacol* 248: 242-8. 17. Segawa M, Sekine S, Sato T et al (2018) Increased susceptibility to troglitazone-induced mitochondrial permeability transition in type 2 diabetes mellitus model rat. *J Toxicol Sci* 43: 339-351. 18. He H, Tao H, Xiong H et al (2014) Rosiglitazone causes cardiotoxicity via peroxisome proliferator-activated receptor γ-independent mitochondrial oxidative stress in mouse hearts. *Toxicol Sci* 138: 468-81. 19. Nadanaciva S, Dykens JA, Bernal A et al (2007) Mitochondrial impairment by PPAR agonists and statins identified via immunocaptured OXPHOS complex activities and respiration. *Toxicol Appl Pharmacol* 223: 277-87. 20. Ong MM, Latchoumycandane C, Boelsterli UA (2007) Troglitazone-induced hepatic necrosis in an animal model of silent genetic mitochondrial abnormalities. *Toxicol Sci* 97: 205-13. 21. Lee YH, Goh WW, Ng CK et al (2013) Integrative toxicoproteomics implicates impaired mitochondrial glutathione import as an off-target effect of troglitazone. *J Proteome Res* 12: 2933-45. 22. Liao X, Wang Y, Wong CW (2010) Troglitazone induces cytotoxicity in part by promoting the degradation of peroxisome proliferator-activated receptor γ co-activator-1α protein. *Br J Pharmacol* 161: 771-81. 23. Rachek LI, Yuzefovych LV, Ledoux SP et al (2009) Troglitazone, but not rosiglitazone, damages mitochondrial DNA and induces mitochondrial dysfunction and cell death in human hepatocytes. *Toxicol Appl Pharmacol* 240: 348-54. 24. Rezaiean Mehrabadi A, Jamshidzadeh A, Rashedinia M et al (2015) Study of the Effects of ATP Suppliers and Thiol Reductants on Toxicity of Pioglitazone in Isolated Rat Liver Mitochondria. *Iran J Pharm Res* 14: 825-32. 25. Goda K, Takahashi T, Kobayashi A et al (2016) Usefulness of in vitro combination assays of mitochondrial dysfunction and apoptosis for the estimation of potential risk of idiosyncratic drug induced liver injury. *J Toxicol Sci* 41: 605-15. 26. Cannon DT, Liu J, Sakurai R et al (2016) Impaired Lung Mitochondrial Respiration Following Perinatal Nicotine Exposure in Rats. *Lung* 194: 325-8. 27. Miglio G, Rosa AC, Rattazzi L et al (2009) PPARgamma stimulation promotes mitochondrial biogenesis and prevents glucose deprivation-induced neuronal cell loss. *Neurochem Int* 55: 496-504. 28. Li Y, Zhang X, Tong N (2013) Pioglitazone ameliorates palmitate induced impairment of mitochondrial morphology and function and restores insulin level in beta cells. *Pharmazie* 68: 270-3. 29. Bruin JE, Petrik JJ, Hyslop JR et al (2010) Rosiglitazone improves pancreatic mitochondrial function in an animal model of dysglycemia: role of the insulin-like growth factor axis. *Endocrine* 37: 303-11. 30. Ko J, Park JH, Park YS et al (2016) PPAR-γ activation attenuates deltamethrin-induced apoptosis by regulating cytosolic PINK1 and inhibiting mitochondrial dysfunction. *Toxicol Lett* 260: 8-17. 31. Fujimoto K, Kumagai K, Ito K et al (2009) Sensitivity of liver injury in heterozygous Sod2 knockout mice treated with troglitazone or acetaminophen. *Toxicol Pathol* 37: 193-200. 32. Hoffmann BR, El-Mansy MF, Sem DS et al (2012) Chemical proteomics-based analysis of off-target binding profiles for rosiglitazone and pioglitazone: clues for assessing potential for cardiotoxicity. *J Med Chem* 55: 8260-71. 33. Jaeschke H (2007) Troglitazone hepatotoxicity: are we getting closer to understanding idiosyncratic liver injury? *Toxicol Sci* 97: 1-3. |

| **Haloperidol** |
| --- |
| **Mode of action** |
| Haloperidol inhibits the effects of dopamine and increases its turnover. However, the exact mechanism of action is not fully understood. Dopamine overactivity can be presynaptic (an excess of dopamine release from dopamine nerve terminals) or post-synaptic (an increase in the density of D2 receptors or an increase in post-receptor action). Traditional antipsychotics, such as haloperidol, bind more tightly than dopamine itself to the dopamine D2 receptor, with dissociation constants that are lower than that for dopamine. |
| **Theoretical effects on mitochondrial function** |
| Inhibition of MRC I and inhibition of OXPHOS |
| **Known effects on mitochondrial function** |
| Haloperidol caused inhibition of MRC complex I activity in mice brain slices, which was accompanied by a decrease in GSH status. Although the mechanism is uncertain, it has been suggested that haloperidol treatment may cause an increased auto-oxidation of dopamine, resulting in the generation of Superoxide, which has been shown to be an effective inhibitor of complex I activity. |
| **Effects measured/seen in mitochondrial patients, mitochondrial cell lines or normal cell lines** |
| No reports in clinical practice/in vivo. Endothelial cells, immortalised (EBV) lymphoblasts, mononuclear cells, and human brain cortex. MCI inhibition, impaired network dynamics and cellular respiration (in Schizophrenia versus BAD). No studies in mitochondrial disease patients or cell lines. Remaining studies in rodents show variable reduction in MRCE, particular MCI. |
| **References** |
| Altunkaynak BZ, Ozbek E, Unal B et al (2012) Chronic treatment of haloperidol induces pathological changes in striatal neurons of guinea pigs: a light and electron microscopical study. *Drug Chem Toxicol* 35: 406–11.  Balijepalli S, Boyd MR, Ravindranath V (1999) Inhibition of mitochondrial complex I by haloperidol: the role of thiol oxidation. *Neuropharmacology* 38: 567–77.  Balijepalli S, Kenchappa RS, Boyd MR et al (2001) Protein thiol oxidation by haloperidol results in inhibition of mitochondrial complex I in brain regions: comparison with atypical antipsychotics. *Neurochem Int* 38: 425–35.  Barrientos A, Marín C, Miró O et al (1998) Biochemical and molecular effects of chronic haloperidol administration on brain and muscle mitochondria of rats. *J Neurosci Res* 53: 475–81.  Brown S, Taylor NL (2000) Inhibition of mitochondrial succinate oxidation by antipsychotic medication. *Vet Hum Toxicol* 42: 209–11.  Burkhardt C, Kelly JP, Lim YH et al (1993) Neuroleptic medications inhibit complex I of the electron transport chain. *Ann Neurol* 33: 512–7.  Casademont J, Garrabou G, Miró O et al (2007) Neuroleptic treatment effect on mitochondrial electron transport chain: peripheral blood mononuclear cells analysis in psychotic patients. *J Clin Psychopharmacol* 27: 284–8.  Elmorsy E, Smith PA (2015) Bioenergetic disruption of human micro-vascular endothelial cells by antipsychotics. *Biochem Biophys Res Commun* 460: 857–62.  Elmorsy E, Al-Ghafari A, Aggour AM et al (2017) Effect of antipsychotics on mitochondrial bioenergetics of rat ovarian theca cells. *Toxicol Lett* 272: 94–100.  Eyles DW, Pond SM, Van der Schyf CJ et al (2000) Mitochondrial ultrastructure and density in a primate model of persistent tardive dyskinesia. *Life Sci* 66: 1345–50.  Maurer I, Möller HJ (1997) Inhibition of complex I by neuroleptics in normal human brain cortex parallels the extrapyramidal toxicity of neuroleptics. *Mol Cell Biochem* 174: 255–9.  Modica-Napolitano JS, Lagace CJ, Brennan WA et al (2003) Differential effects of typical and atypical neuroleptics on mitochondrial function in vitro. *Arch Pharm Res* 26: 951–9.  Prince JA, Yassin MS, Oreland L (1997) Neuroleptic-induced mitochondrial enzyme alterations in the rat brain. *J Pharmacol Exp Ther* 280: 261–7.  Rosenfeld M, Brenner-Lavie H, Ari SG-B et al (2011) Perturbation in mitochondrial network dynamics and in complex I dependent cellular respiration in schizophrenia. *Biol Psychiatry* 69: 980–8.  Sagara Y (1998) Induction of reactive oxygen species in neurons by haloperidol. *J Neurochem* 71: 1002–12.  Shinoda Y, Tagashira H, Bhuiyan MS et al (2016) Haloperidol aggravates transverse aortic constriction-induced heart failure via mitochondrial dysfunction. *J Pharmacol Sci* 131: 172–83.  Streck EL, Rezin GT, Barbosa LM et al (2007) Effect of antipsychotics on succinate dehydrogenase and cytochrome oxidase activities in rat brain. *Naunyn Schmiedebergs Arch Pharmacol* 376: 127–33.  Wei Z, Qi J, Dai Y et al (2009) Haloperidol disrupts Akt signalling to reveal a phosphorylation-dependent regulation of pro-apoptotic Bcl-XS function. *Cell Signal* 21: 161–8. |

| **Ketamine** |
| --- |
| **Mode of action** |
| Ketamine (and memantine) antagonizes N-methyl-d-aspartate receptors (NMDARs), a glutamate receptor subfamily, by blocking the receptor-associated ion channel. |
| **Theoretical effects on mitochondrial function** |
| Prolonged ketamine exposure produces an increase in NMDA receptor expression that allows for a toxic influx of calcium into neurons once ketamine is removed from the system, leading to elevated ROS generation and neuronal cell death. The application of antioxidants such as L‐carnitine appears to be promising for preventing or reversing the toxic effects of ketamine. Involvement of ketamine in either toxicity and/or protection may be determined or directed by the dose of ketamine used, the duration of exposure, the route of administration, and the stage of development at the time of exposure [ref 8]. |
| **Known effects on mitochondrial function** |
| \| **Type of Study** \| **Model Used** \| **Effect Measured** \| **Effect seen/ Mechanism** \| **Year** \| **Ref** \| \| --- \| --- \| --- \| --- \| --- \| --- \| \| **In vivo** \| Adult male rats \| received a single dose of ketamine (50, 100, or 150 mg/kg IP) or a combination of ketamine and N-nitro-L-arginine (3 mg/kg IP). Animals were killed 6 hours after treatment. Brain and blood samples were collected for plasma NO determination and mitochondria isolation. Several variables of brain mitochondrial function were evaluated. \| Acute ketamine administration impaired the function of mitochondrial complex I leading to increased mtNOS activity, increased generation of hydrogen peroxide and NO, resulting in superoxide dismutase triggering, and improved antioxidant activity. \| **2015** \| **1** \| \| **In vivo** \| Rats administered ketamine for 7 consecutive days \| Behavioural evaluation and activities of mitochondrial respiratory chain complexes I, II, I-III and IV in multiple brain regions \| hyperlocomotion occurred in the ketamine group 1 and 3 h after the last injection; found that ketamine administration affects the respiratory chain, altering the activity of respiratory chain complexes in the striatum and hippocampus after 1 h, those in the prefrontal cortex and hippocampus after 3 h and those in the prefrontal cortex and striatum 6 h after the last administration of ketamine; ]authors suggest that ketamine alters the behaviour of rats and changes the activity of respiratory chain complexes in multiple brain regions at different time points \| **2011** \| **7** \| \| **In vivo** \| Mice \| Perinatal treatment with ketamine induced persistent changes in the reduced glutathione/oxidized glutathione (glutathione disulfide) ratio in the medial PFC, indicating long-lasting increases in oxidative stress. \| antioxidant (NAC) treatment in male mice prevents mitochondrial and synaptic changes in an NMDA receptor dysfunction model of schizophrenia \| **2017** \| **12** \| |
| **Effects measured/seen in mitochondrial patients, mitochondrial cell lines or normal cell lines** |
| Few reports of ketamine anaesthesia for mitochondrial disease, and no convincing adverse effects reported except central DI in a patient receiving prolonged ketamine infusion in ICU   \| **Type of Study** \| **Model Used** \| **Effect Measured** \| **Effect seen/ Mechanism** \| **Year** \| **Ref** \| \| --- \| --- \| --- \| --- \| --- \| --- \| \| **In vivo** \| 30m with Leigh syndrome \| extracorporeal shockwave lithotripsy - Anaesthesia was induced with ketamine and midazolam im, and N2O in oxygen, and maintained with propotol and N2O. No volatile anaesthetics were used. \| perianaesthetic course was uneventful \| **1997** \| **3** \| \| **In vivo** \| 57y female mito myopathy \| Laparoscopic cholecystectomy for cholelithiasis; total intravenous anesthesia with propofol, fentanyl, and ketamine \| No serious acidosis, hyperlactemia, hypothermia, nor prolonged recovery from the anesthesia was observed \| **2004** Japanese (English abstract) \| **4** \| \| **In vivo** \| 22y woman with mitochondrial status epilepticus \| Status epilepticus not responsive to benzodiazepines, phenytoin, thiopental, and propofol \| SE was terminated within days after supplemental administration of continuous ketamine infusion to midazolam. \| **2008** \| **5** \| \| **In vivo** \| 58y woman with MELAS \| emergency laparotomy - Anesthesia was induced by rapid-sequence fashion and maintained using midazolam, propofol, ketamine, fentanyl and vecuronium \| uneventful peri-operative course \| **2010**  Japanese (English abstract) \| **6** \| \| **In vivo** \| 2y girl with LCHAD deficiency \| Continuous ketamine infusion was used for the sedation and facilitation of mechanical ventilation for respiratory failure associated with episode of pneumonia \| Developed transient central diabetes insipidus attributed to ketamine infusion \| **2014** \| 9 \| \| **In vivo** \| 27y woman with mito myopathy and complex III deficiency \| combination of fentanyl (100µg), ketamine, and methohexital for induction, as well as methohexital, sufentanil, and dexmedetomidine for the maintenance of general anesthesia during 2 consecutive surgeries (laparoscopic resection of adenocarcinoma of sigmoid, and resection of lung met) \| uneventful postoperative course \| **2017** \| **10** \| \| **In vivo** \| 26 patients with genetically proven mitochondrial disease  underwent 65 GAs (only 2 received ketamine) \| Induction of anaesthesia was intravenous  in the majority of patients: propofol n= 34 (52%), thiopental n= 1 (1.5%), **ketamine n= 2 (3%)** \| Patient 5 has a diagnosis of combined oxidative phosphorylation deficiency 11 and received sevoflurane and ketamine at induction of GA with sevoflurane for maintenance. She became  hypotensive towards the end of her 2-h GA managed  with a 20 ml/kg colloid bolus, stabilised successfully with appropriate intervention, did not require ICU admission \| **2017** \| **11** \| |
| **References** |
| 1. Venancio C, Felix L, Almeida V et al (2015) Acute ketamine impairs mitochondrial function and promotes superoxide dis-mutase activity in the rat brain*. Anesth Analg* 120: 320-8.  2. Ito H, Uchida T, Makita K (2015) Ketamine causes mitochondrial dys-function in human induced pluripotent stem cell-derived neurons. *PLoS One* 10: e0128445.  3. Shenkman Z, Krichevski I, Elpeleg ON et al (1997) Anaesthetic management of a patient with Leigh's syndrome. *Can J Anaesth* 44: 1091-5.  4. Kushikata T, Yatsu Y, Kubota T (2004) [Total intravenous anesthesia with propofol, ketamine, and fentanyl (PFK) for a patient with mitochondrial myopathy]. *Masui* 53: 178-80. Japanese.  5. Prüss H, Holtkamp M (2008) Ketamine successfully terminates malignant status epilepticus. *Epilepsy Res* 82: 219-22.  6. Imai Y, Kitamura T, Kawamura G et al (2010) [Anesthetic management of emergency total gastrectomy in a patient with mitochondrial encephalomyopathy: a case of gastric perforation accompanied by systemic inflammatory response syndrome]. *Masui* 59: 765-9. Japanese.  7. de Oliveira L, Fraga DB, De Luca RD et al (2011) Behavioral changes and mitochondrial dysfunction in a rat model of schizophrenia induced by ketamine. *Metab Brain Dis* 26: 69-77.  8. Wang C, Liu F, Patterson TA et al (2013) Preclinical assessment of ketamine. *CNS Neurosci Ther* 19: 448-53.  9. Hatab SZ, Singh A, Felner EI, Kamat P (2014) Transient central diabetes insipidus induced by ketamine infusion. Ann Pharmacother 48: 1642-5.  10. Woodward EL, Xiong Z (2017) Use of Methohexital and Dexmedetomidine for Maintenance of Anesthesia in a Patient With Mitochondrial Myopathy: A Case Report. *A A Case Rep* 8: 33-5.  11. Smith A, Dunne E, Mannion M et al (2017) A review of anaesthetic outcomes in patients with genetically confirmed mitochondrial disorders. *Eur J Pediatr* 176: 83-88.  12. Phensy A, Driskill C, Lindquist K et al (2017) Antioxidant Treatment in Male Mice Prevents Mitochondrial and Synaptic Changes in an NMDA Receptor Dysfunction Model of Schizophrenia. *eNeuro* 4. |

| **Lidocaine** |
| --- |
| **Mode of action** |
| Lidocaine is a common local or regional anesthetic and antiarrhythmic drug.  Lidocaine alters signal conduction in neurons by blocking the fast voltage gated sodium (Na+) channels in the neuronal cell membrane that are responsible for signal propagation. With sufficient blockage the membrane of the postsynaptic neuron will not depolarize and will thus fail to transmit an action potential. This creates the anaesthetic effect by not merely preventing pain signals from propagating to the brain but by aborting their birth in the first place.  Lidocaine is a sodium-channel blocker that does not affect the QRS complex and is thus classified as a class-1b antiarrhythmic drug. In cardiology, lidocaine is used against ventricular arrhythmias. |
| **Effects on mitochondrial function** |
| - Compared to control neutrophils, the ATP concentration and the mitochondrial transmembrane potential were significantly reduced in neutrophils exposed to lidocaine. Lidocaine also induced structural changes in neutrophil mitochondria and induced apoptosis.  - The adverse effects of lidocaine on mitochondrial bioenergetics are mostly inhibitory to the electron transport chain, which results in decreasing respiration of non-beating myocardium in vitro. But lidocaine reduce cellular energy demands.  - The physiological alterations inducing lidocaine toxicity participate in mitochondrial depolarization, with the excessive generation of ROS/ RNS, and intracellular calcium accumulation.  - The chondrotoxicity is associated with mitochondrial dysfunction resulting from damage to the mitochondrial genome. This leads to a decrease in energy production and, ultimately, to cell death.  - In cells obtained from the rat dorsal root ganglion, lidocaine induced apoptosis correlated with mitochondrial membrane depolarization, cytochrome-c release from mitochondria into the cytosol, and activation of caspases.  - Lidocaine depressed energy consumption by delaying ischemia-induced membrane depolarization and reduced the rate of energy utilization in Ischemic Mouse Brain. |
| **References** |
| Aburawi EH, Souid A-K (2014) Inhibition of murine cardiomyocyte respiration by amine local anesthetics. European *J Drug Metab Pharmacokin* 39: 293–9.  Boone CHT, Grove RA, Adamcova D et al (2017) Oxidative stress, metabolomics profiling, and mechanism of local anesthetic induced cell death in yeast. *Redox Biol* 12: 139-49.  Ellis Z, Bloomer C (2005) [Outpatient anesthesia for oral surgery in a juvenile with Leigh disease.](https://www.ncbi.nlm.nih.gov/pubmed/16048155) *Anesth Prog* 52: 70-3.  Grishko V, Xu M, Wilson G et al (2010) [Apoptosis and mitochondrial dysfunction in human chondrocytes following exposure to lidocaine, bupivacaine, and ropivacaine.](https://www.ncbi.nlm.nih.gov/pubmed/20194319) *J Bone Joint Surg Am* 92: 609-18.  Kawasaki C, Kawasaki T, Ogata M et al (2010) Lidocaine enhances apoptosis and suppresses mitochondrial functions of human neutrophil in vitro. *J Trauma* 68: 401-8.  Onizuka S, Yonaha T, Tamura R et al (2011) [Lidocaine depolarizes the mitochondrial membrane potential by intracellular alkalization in rat dorsal root ganglion neurons.](https://www.ncbi.nlm.nih.gov/pubmed/21212988) *J Anesth* 25: 229-39.  Rosaeg OP, Morrison S, MacLeod JP (1996) Anaesthetic management of labour and delivery in the parturient with mitochondrial myopathy. *Can J Anaesth* 43: 403–7.  Sasano N, Fujita Y, So M et al(2007) Anesthetic management of a patient with mitochondrial myopathy, encephalopathy, lactic acidosis, and stroke-like episodes (MELAS) during laparotomy. *J Anesth* 21: 72–5.  [Seyfried FJ](https://www.ncbi.nlm.nih.gov/pubmed/?term=Seyfried%20FJ%5BAuthor%5D&cauthor=true&cauthor_uid=15840992), [Adachi N](https://www.ncbi.nlm.nih.gov/pubmed/?term=Adachi%20N%5BAuthor%5D&cauthor=true&cauthor_uid=15840992), [Arai T](https://www.ncbi.nlm.nih.gov/pubmed/?term=Arai%20T%5BAuthor%5D&cauthor=true&cauthor_uid=15840992). (2005) Suppression of energy requirement by lidocaine in the ischemic mouse brain. [*J Neurosurg Anesthesiol.*](https://www.ncbi.nlm.nih.gov/pubmed/?term=Suppression+of+Energy+Requirement+by+Lidocaine+in+the+Ischemic+Mouse+Brain+Frank-Joachim) 17: 75-81. |

| **Linezolid** |
| --- |
| **Mode of action** |
| The drug works by inhibiting the initiation of bacterial protein synthesis. It does so by preventing the formation of the initiation complex, composed of the 30S and 50S subunits of the ribosome, tRNA, and mRNA. Linezolid binds to the 23S portion of the 50S subunit (the center of peptidyl transferase activity), close to the binding sites of chloramphenicol, lincomycin, and other antibiotics. |
| **Theoretical effects on mitochondrial function** |
| Linezolid is toxic to mitochondria, probably because of the similarity between mitochondrial and bacterial ribosomes. Lactic acidosis, a potentially life-threatening buildup of lactic acid in the body, may also occur due to mitochondrial toxicity. |
| **Known effects on mitochondrial function** |
| The drug decreases the activity of mitochondrial respiratory chain without ultrastructural mitochondrial abnormalities and without mutations or depletion of mtDNA. |
| **Effects measured/seen in mitochondrial patients, mitochondrial cell lines or normal cell lines** |
| The most common side effect in clinical practice, described in people without mitochondrial disease treated with linezolid, is hyperlactatemia, linked to depression mitochondrial protein sintesys, and less frequently optic or peripheral neuropathy.  On normal cell lines of patients treated with linezolid, many studies described that this drug reduced protein expression for the mitochondrially coded, transcribed, and translated COX subunit (COX I, II and IV); COX activity was also found to be decreased.These reductions were observed despite the numbers of COX-II mitochondrial RNA transcripts being abnormally increate and the mitochondrial DNA content remaining stable. No alteration have been found in mitochondrial DNA amount. In another study, linezolid reduced mitochondrial protein levels, complex IV activity, and mitochondrial mass in PBMC and was associated with a trend toward an increase in the rate of apoptosis. In skin tissue, mitochondrial mass increased within nerve fibers, accompanied by subclinical axonal swelling.  Most of the described mitochondrial abnormalities returned to control ranges after linezolid withdrawal.  Mitochondrial haplogroup U, mutations in 12S rRNA, and the m.2706A→G, m.3197T→C, and m.3010G→A polymorphisms in 16S rRNA showed a trend toward an association with increased mitochondrial and clinical adverse effects.  No study exist on mito cell lines.  In a patient with MELAS, linezolid induced an early lactic acidosis (after 4 pills). |
| **References** |
| Apodaca AA, Rakita RM (2003) Linezolid-induced lactic acidosis. *N Engl J Med* 348: 86-7.  Bobylev I, Maru H, Joshi AR et al (2016) Toxicity to sensory neurons and Schwann cells in experimental linezolid-induced peripheral neuropathy. *J Antimicrob Chemother* 71(3):685-91.  Contou D, Fichet J, Grimaldi D et al (2011) Early life-threatening lactic acidosis following a single infusion of linezolid. *Int J Antimicrob Agents* 38: 84-5.  Cope TE, McFarland R, Schaefer A (2011) Rapid-onset, linezolid-induced lactic acidosis in MELAS. *Mitochondrion* 11: 992-3.  Del Pozo JL, Fernández-Ros N, Sáez E et al (2014) Linezolid-induced lactic acidosis in two liver transplant patients with the mitochondrial DNA A2706G polymorphism. *Antimicrob Agents Chemother* 58: 4227-9  De Vriese AS, Coster RV, Smet J et al (2006) Linezolid-induced inhibition of mitochondrial protein synthesis. *Clin Infect Dis* 42: 1111-7  Flanagan S, McKee EE2, Das D et (2015) Nonclinical and pharmacokinetic assessments to evaluate the potential of tedizolid and linezolid to affect mitochondrial function. *Antimicrob Agents Chemother* 59(1):178-85.  Garrabou G, Soriano A, López S et al (2007) Reversible inhibition of mitochondrial protein synthesis during linezolid-related hyperlactatemia. *Antimicrob Agents Chemother* 51: 962-7  Garrabou G, Soriano À, Pinós T et al (2017) Influence of Mitochondrial Genetics on the Mitochondrial Toxicity of Linezolid in Blood Cells and Skin Nerve Fibers. *Antimicrob Agents Chemother* 61.  Javaheri M, Khurana RN, O'hearn TM et al (2007) Linezolid‐induced optic neuropathy: a mitochondrial disorder? *Br J Ophthalmol* 91: 111-5.  Kraleti S, Soultanova I (2013) Pancytopenia and lactic acidosis associated with linezolid use in a patient with empyema. *J Ark Med Soc* 110(4):62-3.  McKee EE, Ferguson M, Bentley AT et al (2006) Inhibition of mammalian mitochondrial protein synthesis by oxazolidinones. *Antimicrob Agents Chemother* 50: 2042-9.  Ozkaya-Parlakay A, Kara A, Celik M et al (2014) Early lactic acidosis associated with linezolid therapy in paediatric patients. *Int J Antimicrob Agents* 44: 334-6.  Palenzuela L, Hahn NM, Nelson RP Jr et al (2005) Does linezolid cause lactic acidosis by inhibiting mitochondrial protein synthesis? *Clin Infect Dis* 40: e113-6.  Protti A, Ronchi D, Bassi G et al (2015) Changes in Whole-Body Oxygen Consumption and Skeletal Muscle Mitochondria During Linezolid-Induced Lactic Acidosis. *Crit Care Med* 44: e579-82.  Rucker JC, Hamilton SR, Bardenstein D et al (2006) Linezolid-associated toxic optic neuropathy. *Neurology* 66(4):595-8.  Soriano A, Miró O, Mensa J (2005) Mitochondrial toxicity associated with linezolid. *N Engl J Med* 353: 2305-6.  Taketani T, Kanai R, Fukuda S et al (2009) Pure red cell precursor toxicity by linezolid in a pediatric case. *J Pediatr Hematol Oncol* 31: 684-6. |

| **Midazolam** |
| --- |
| **Mode of action** |
| The therapeutic as well as adverse effects of midazolam are due to its effects on the GABA_A_ receptors; midazolam does not activate GABA_A_ receptors directly but, as with other benzodiazepines, it enhances the effect of the neurotransmitter GABA on the GABA_A_ receptors (↑ frequency of Cl− channel opening) resulting in neural inhibition. |
| **Theoretical effects on mitochondrial function** |
| It is supposed that midazolam has a scavenger effect in ROS preventing mitochondrial damage and subsequently prevent peroxidation and neuronal damage |
| **Known effects on mitochondrial function** |
| Midazolam primarily acts as a mitochondrial electron transport inhibitor. This inhibition is mainly due to the fact that midazolam decreases NADH ubiquinone reductase (complex I) and ubiquinol cytochrome c reductase (complex III) activities, but it also inhibits complex II activity. Spectrophotometric measurements of redox states of rat skeletal muscle mitochondria cytochromes show a decrease in the reduction of aa_3_ and c+c_1_ cytochromes in the presence of the benzodiazepine. Midazolam significantly decreased the reduced ubiquinone/total ubiquinone ratio (evaluated by means of HPLC and electrochemical detection) in rat liver mitochondria in both Phydroxybutyrate and succinate. These effects of midazolam, not necessarily related to the preanaesthetic and hypnotic action are probably mediated via mitochondrial benzodiazepine receptors. |
| **Effects measured/seen in mitochondrial patients, mitochondrial cell lines or normal cell lines** |
| The most common side effect of midazolam is respiratory depression. No specific side effect has been reported in mitochondrial patients. |
| **References** |
| [Ghezzi F](https://www.ncbi.nlm.nih.gov/pubmed/?term=Ghezzi%20F%5BAuthor%5D&cauthor=true&cauthor_uid=29069620), [Monni L](https://www.ncbi.nlm.nih.gov/pubmed/?term=Monni%20L%5BAuthor%5D&cauthor=true&cauthor_uid=29069620), [Corsini S](https://www.ncbi.nlm.nih.gov/pubmed/?term=Corsini%20S%5BAuthor%5D&cauthor=true&cauthor_uid=29069620) et al (2017) Propofol Protects Rat Hypoglossal Motoneurons in an In Vitro Model of Excitotoxicity by Boosting GABAergic Inhibition and Reducing Oxidative Stress. [*Neuroscience.*](https://www.ncbi.nlm.nih.gov/pubmed/29069620) 367: 15-33.  Hata M, Kobayashi K, Yoshino F et al (2011) Direct assessment of the antioxidant properties of midazolam by electron spin resonance spectroscopy*. J Anesth* 25:765–9.  [Li Y](https://www.ncbi.nlm.nih.gov/pubmed/?term=Li%20Y%5BAuthor%5D&cauthor=true&cauthor_uid=29634998), [Li X](https://www.ncbi.nlm.nih.gov/pubmed/?term=Li%20X%5BAuthor%5D&cauthor=true&cauthor_uid=29634998), [Zhao J](https://www.ncbi.nlm.nih.gov/pubmed/?term=Zhao%20J%5BAuthor%5D&cauthor=true&cauthor_uid=29634998) et al (2018) Midazolam Attenuates Autophagy and Apoptosis Caused by Ketamine by Decreasing Reactive Oxygen Species in the Hippocampus of Fetal Rats. [*Neuroscience*.](https://www.ncbi.nlm.nih.gov/pubmed/29634998) 388:460-71.  Lunardi N, Ori C, Erisir A et al (2010) [General anesthesia causes long-lasting disturbances in the ultrastructural properties of developing synapses in young rats.](https://www.ncbi.nlm.nih.gov/pubmed/19626389) *Neurotox Res*. 17:179-88. |

| **NSAIDs** |
| --- |
| **Mode of action** |
| Nonsteroidal anti-inflammatory drug (NSAIDs) work as COX inhibitors. COX are involved in the synthesis of prostaglandins which cause inflammation. There are two types of NSAID available: non-selective and COX-2 selective. Most NSAIDs are non-selective, and inhibit the activity of both COX-1 and COX-2. Side effects depend on the specific drug, but largely include an increased risk of gastrointestinal, heart, liver and kidney disease. |
| **Theoretical effects on mitochondrial function** |
| These drugs seem to uncouple or inhibit mitochondrial oxidative phosphorylation interacting with the phospholipids in the target membrane, making the membrane permeable to protons, that is, they uncouple by their protonophoric actions. |
| **Known effects on mitochondrial function** |
| Except for some NSAIDs such as nabumetone and naproxen, the other drugs stimulated basal and uncoupled respiration, inhibited ATP synthesis, and collapsed membrane potential in mitochondria (incubated in the presence of either glutamate + malate or succinate) (Rafael Moreno-Sanchez et al. 1999)  Diclofenac and Ibuprofen may alter the morphology of mitochondria, leading to cytochrome c release into cytosol. Ibuprofen acted as an activator of Ca2++ and phosphate in promoting the opening of inner mitochondrial membrane pore (Mitochondrial permeability transition (MPT) of the inner membrane in Ca2++ overload situations is often associated with opening of a Ca2++ induced pore. Opening this pore renders mitochondria leaky and leads to dissipation of membrane potential, uncoupling of energy transduction, loss of pre-accumulated Ca2++ and expansion of the matrix volume) (Ibrahim A. Al-Nasser 2000; M. Moorthy et al. 2008) . One of the mechanisms by which nonsteroidal anti-inflammatory drugs induce renal damage is through oxygen free radicals possibly generated by activated neutrophils and mitochondrial dysfunction (Jayasree Basivireddya 2003). Moreover, the indomethacin seems to induce proteasomal dysfunction and mitochondrial apoptosis in cells (Amanullah A et al. 2018). |
| **Effects measured/seen in mitochondrial patients, mitochondrial cell lines or normal cell lines** |
| The most common side effects described in animals model treated with NSAIDs underling that these drugs uncouple or inhibit mitochondrial oxidative phosphorylation.  One study demonstrated that that nimesulide, meloxicam, piroxicam, and indomethacin behaved as mitochondrial uncouplers, whereas nabumetone exerted a specific inhibition of site 1 of the respiratory chain. Diclofenac was an uncoupler too, but it also affected the adenine nucleotide translocase and the H1-ATPase. Another study concluded that ibuprofen acted as an activator of Ca2++ and phosphate in promoting the opening of inner mitochondrial membrane pore. A study which used rats as an animal model, designed to look at the possible biochemical mechanisms involved in indomethacin-induced renal damage suggested that one of the mechanisms by which nonsteroidal anti-inflammatory drugs induce renal damage is through oxygen free radicals possibly generated by activated neutrophils and mitochondrial dysfunction.  No studies exist on mito cell lines, and no published data on mito patients. |
| **References** |
| Al-Nasser IA (2000) Ibuprofen-induced liver mitochondrial permeability transition. *Toxicol Lett*. 111: 213-8.  Amanullah A, Mishra R, Upadhyay A et al (2018) Indomethacin elicits proteasomal dysfunctions develops apoptosis through mitochondrial abnormalities. *J Cell Physiol* 233: 1685-99.  Basivireddy J, Jacob M, Pulimood AB et al (2004) Indomethacin-induced renal damage: role of oxygen free radicals. *Biochem Pharmacol.* 67: 587-99.  Bjarnason I, Scarpignato C, Holmgren E et al (2018) Mechanisms of Damage to the Gastrointestinal Tract From Nonsteroidal Anti-Inflammatory Drugs. *Gastroenterology.* 154: 500-14.  Browne GS, Nelson C, Nguyen T et al (1999) Stereoselective and substrate-dependent inhibition of hepatic mitochondria beta-oxidation and oxidative phosphorylation by the non-steroidal anti-inflammatory drugs ibuprofen, flurbiprofen, and ketorolac. *Biochem Pharmacol* 57: 837-44.  Krause MM, Brand MD, Krauss S et al (2003) Nonsteroidal antiinflammatory drugs and a selective cyclooxygenase 2 inhibitor uncouple mitochondria in intact cells. *Arthritis Rheum* 48: 1438-44.  Mazumder S, De R, Sarkar S et al (2016) Selective scavenging of intra-mitochondrial superoxide corrects diclofenac-induced mitochondrial dysfunction and gastric injury: A novel gastroprotective mechanism independent of gastric acid suppression. *Biochem Pharmacol*. 121:33-51.  Moorthy M, Fakurazi S, Ithnin H (2008) Morphological alteration in mitochondria following diclofenac and ibuprofen administration. *Pak J Biol Sci* 11: 1901-8.  Moreno-Sánchez R, Bravo C, Vásquez C et al (1999) Inhibition and uncoupling of oxidative phosphorylation by nonsteroidal anti-inflammatory drugs: study in mitochondria, submitochondrial particles, cells, and whole heart. *Biochem Pharmacol* 57: 743-52.  Salgueiro-Pagadigorria CL, Kelmer-Bracht AM, Bracht A et al (2004) Naproxen affects Ca(2+) fluxes in mitochondria, microsomes and plasma membrane vesicles. *Chem Biol Interact*. 147: 49-63.  Sandoval-Acuña C, Lopez-Alarcón C, Aliaga ME et al (2012) Inhibition of mitochondrial complex I by various non-steroidal anti-inflammatory drugs and its protection by quercetin via a coenzyme Q-like action. *Chem Biol Interact* 199: 18-28.  Van Leeuwen JS, Unlü B, Vermeulen NP et al (2012) Differential involvement of mitochondrial dysfunction, cytochrome P450 activity, and active transport in the toxicity of structurally related NSAIDs. *Toxicol In Vitro* 26: 197-205. |

| **Oxcarbazepine** |
| --- |
| **Mode of action** |
| Oxcarbazepine is a derivative of carbamazepine but minor structural alterations between them result in different metabolism of the two medications. Similar to carbamazepine, oxcarbazepine binds to inactivated sodium channels on neurons and prevents high frequency repetitive firing (Stefani et al 1995). Oxcarbazepine also inhibits release of the excitatory neurotransmitter glutamate (Waldmeier et al 1995). Oxcarbazepine is rapidly converted to an active metabolite known as licarbazepine which appears to be responsible for most of the anticonvulsant effect of oxcarbazepine. |
| **Theoretical effects on mitochondrial function** |
| Oxcarbazepine increase reactive oxygen species production in cultured rat hippocampal neurons (Araújo et al 2004). |
| **References** |
| [Araújo](https://onlinelibrary.wiley.com/action/doSearch?ContribAuthorStored=Ara%C3%BAjo%2C+In%C3%AAs+M) IM, [Ambrósio](https://onlinelibrary.wiley.com/action/doSearch?ContribAuthorStored=Ambr%C3%B3sio%2C+Ant%C3%B3nio+Francisco) AF, [Leal](https://onlinelibrary.wiley.com/action/doSearch?ContribAuthorStored=Leal%2C+Ermelindo+C) EC et al (2004) Neurotoxicity Induced by Antiepileptic Drugs in Cultured Hippocampal Neurons: A Comparative Study between Carbamazepine, Oxcarbazepine, and Two New Putative Antiepileptic Drugs, BIA 2‐024 and BIA 2‐093. [*Epilepsia.*](https://www.ncbi.nlm.nih.gov/pubmed/?term=In%C3%AAs+M.+Ara%C3%BAjo+and+Ant%C3%B3nio+Francisco+Ambr%C3%B3sio) 45: 1498-505.  Stefani A, Pisani A, De Murtas M et al (1995) Action of GP47779, the active metabolite of oxcarbazepine, on the corticostriatal system. II. Modulation of high-voltage-activated calcium currents. *Epilepsia* 36: 997–1002.  Waldmeier PC, Baumann PA, Wicki P et al (1995) Similar potency of carbamazepine, oxcarbazepine, and lamotrigine in inhibiting the release of glutamate and other neurotransmitters. *Neurology* 45: 1907–13. |

| **Paracetamol** |
| --- |
| **Mode of action** |
| Not completely understood. Weak anti-inflammatory properties but analgesic and anti-pyretic. Selective inhibition of cyclo-oxygenase expressed in brain but not in extra-CNS tissues (Flower and Vane 1972). Not a direct effect on cyclo-oxygenase. A metabolite AM404 may modulate the endogenous cannabinoid system. Antioxidant properties? (Tripathy and Grammas 2009) |
| **Theoretical effects on mitochondrial function** |
| Concern regarding impact of therapeutic doses of paracetamol on mitochondrial respiratory chain function in patients with pre-existing mitochondrial dysfunction. |
| **Known effects on mitochondrial function** |
| N-acetyl-para-benzoquinone imine (NAPQI) increases oxidative stress and causes a severe impairment of mitochondrial function leading to a profound depletion of ATP and activation of c-Jun N-terminal (JNK) in acute liver injury due to paracetamol overdose.  Paracetamol toxicity impairs mitochondrial respiration in rat hepatocytes in vivo and decreases Complex II (succinate dehydrogenase) and to a lesser extent Complex I (NADH dehydrogenase) activity in mouse hepatocytes (Donnelly et al 1994, Burcham and Harman 1991)  Paracetamol may interfere with supercomplex formation via the mitochondrial negative regulator MCJ (modulator of complex I) leading to a decreased production of ATP and increased generation of ROS (Barbier-Torres et al 2017).  There are also reports of beneficial effects on mitochondrial dysfunction in rats exposed to cerebral ischaemia (Baliga et al 2010). |
| **References** |
| Baliga SS, Jaques-Robinson KM, Hadzimichalis NM et al (2010) [Acetaminophen reduces mitochondrial dysfunction during early cerebral postischemic reperfusion in rats.](https://www.ncbi.nlm.nih.gov/pubmed/20079345) *Brain Res.* 1319: 142-54.  Barbier-Torres L, Iruzubieta P, Fernández-Ramos D et al (2017) [The mitochondrial negative regulator MCJ is a therapeutic target for acetaminophen-induced liver injury.](https://www.ncbi.nlm.nih.gov/pubmed/29233977) *Nat Commun*. 8: 2068.  Burcham PC, Harman AW (1991) Acetaminophen toxicity results in site- specific mitochondrial damage in isolated mouse hepatocytes. *J. Biol. Chem.* 266: 5049–54.  Donnelly PJ, Walker RM, Racz WJ (1994) Inhibition of mitochondrial respiration in vivo is an early event in acetaminophen-induced hepatotoxicity. *Arch. Toxicol.* 68: 110–8.  Flower RJ, Vane JR (1972) Inhibition of prostaglandin synthetase in brain explains the anti-pyretic activity of paracetamol (4-acetamidophenol) *Nature.* 240: 410–1.  Tripathy D, Grammas P (2009) Acetaminophen protects brain endothelial cells against oxidative stress. *Microvasc Res.*77: 289–96. |

| **Perampanel** |
| --- |
| **Mode of action** |
| Perampanel is a non-competitive antagonist of α-amino-3-hydroxy-5-methyl-4-isoxazolepropionic acid (AMPA) receptors, a major subtype of ionotropic glutamate receptors. By inhibiting AMPA receptors, perampanel inhibits the excitatory neurotransmitter activity of L-glutamate at many of the synapses in the central nervous system. These physiological effects decrease synaptic transmission in the central nervous system, slow neuronal excitation, and lessen seizure activity. |
| **Theoretical effects on mitochondrial function** |
| Sustained AMPA receptor activation has been shown to cause a number defects in mitochondrial function including mitochondrial Ca^2+^ overload, rapid loss of mitochondrial membrane potential, permeability transition pore opening, elevated ROS production, decreased glutathione antioxidant defense, and apoptosis. Given that perampanel inhibits AMPA receptor activation, drug treatment may prevent these toxic mitochondrial effects and improve bioenergetic function. |
| **Known effects on mitochondrial function** |
| No reports on the direct effects of perampanel on mitochondrial function or toxicity have been published to the reviewers knowledge. |
| **Effects measured/seen in mitochondrial patients, mitochondrial cell lines or normal cell lines** |
| To the reviewers knowledge no studies have directly examined the effects of perampanel on mitochondrial function exist. However, conclusions drawn across animal model and clinical case studies could provide insight into how perampanel treatment may translate to patients with mitochondrial disease. ATAD1, a gene that decreases AMPA receptor density, has been suggested to promote the maintenance of mitochondrial membrane quality control mechanisms. In both patients with ATAD1 mutations and ATAD1 knockout mice, increased AMPA receptor density was accompanied by enhanced seizure activity that was reversed by treatment with perampanel. However, it is currently unclear how the role of ATAD1 in mitochondrial quality control may relate to patient phenotypes and whether perampanel treatment can mitigate defects in mitochondrial integrity. It should be noted that common side effects observed with perampanel treatment such as the loss of coordination, weakness, vision loss, and fatigue may be heightened in already compromised mitochondrial disease patients. The current literature suggests Perampanel may be cautiously administered to epileptic mitochondrial disease patients since mitochondrial toxicity has not been definitively determined. |
| **References** |
| [Ahrens-Nicklas RC](https://www.ncbi.nlm.nih.gov/pubmed/?term=Ahrens-Nicklas%20RC%5BAuthor%5D&cauthor=true&cauthor_uid=28180185), [Umanah GK](https://www.ncbi.nlm.nih.gov/pubmed/?term=Umanah%20GK%5BAuthor%5D&cauthor=true&cauthor_uid=28180185), [Sondheimer N](https://www.ncbi.nlm.nih.gov/pubmed/?term=Sondheimer%20N%5BAuthor%5D&cauthor=true&cauthor_uid=28180185) et al (2017) Precision therapy for a new disorder of AMPA receptor recycling due to mutations in ATAD1. *Neurol Genet* 3: e130.  [Chen YC](https://www.ncbi.nlm.nih.gov/pubmed/?term=Chen%20YC%5BAuthor%5D&cauthor=true&cauthor_uid=24843043), [Umanah GK](https://www.ncbi.nlm.nih.gov/pubmed/?term=Umanah%20GK%5BAuthor%5D&cauthor=true&cauthor_uid=24843043), [Dephoure N](https://www.ncbi.nlm.nih.gov/pubmed/?term=Dephoure%20N%5BAuthor%5D&cauthor=true&cauthor_uid=24843043) et al (2014) Msp1/ATAD1 maintains mitochondrial function by facilitating the degradation of mislocalized tail-anchored proteins. *EMBO J* 33: 1548-64.  Frampton JE (2015) Perampanel: A Review in Drug-Resistant Epilepsy. *Drugs* 75: 1657-8.  [Hanada T](https://www.ncbi.nlm.nih.gov/pubmed/?term=Hanada%20T%5BAuthor%5D&cauthor=true&cauthor_uid=21635236), [Hashizume Y](https://www.ncbi.nlm.nih.gov/pubmed/?term=Hashizume%20Y%5BAuthor%5D&cauthor=true&cauthor_uid=21635236), [Tokuhara N](https://www.ncbi.nlm.nih.gov/pubmed/?term=Tokuhara%20N%5BAuthor%5D&cauthor=true&cauthor_uid=21635236) et al (2011) Perampanel: a novel, orally active, noncompetitive AMPA-receptor antagonist that reduces seizure activity in rodent models of epilepsy. *Epilepsia* 52: 1331-40.  [Hikmat O](https://www.ncbi.nlm.nih.gov/pubmed/?term=Hikmat%20O%5BAuthor%5D&cauthor=true&cauthor_uid=28837072), [Eichele T](https://www.ncbi.nlm.nih.gov/pubmed/?term=Eichele%20T%5BAuthor%5D&cauthor=true&cauthor_uid=28837072), [Tzoulis C](https://www.ncbi.nlm.nih.gov/pubmed/?term=Tzoulis%20C%5BAuthor%5D&cauthor=true&cauthor_uid=28837072) et al (2017) Understanding the Epilepsy in POLG Related Disease. *Int J Mol Sci* 18: 1845.  [Joshi DC](https://www.ncbi.nlm.nih.gov/pubmed/?term=Joshi%20DC%5BAuthor%5D&cauthor=true&cauthor_uid=25944722), [Tewari BP](https://www.ncbi.nlm.nih.gov/pubmed/?term=Tewari%20BP%5BAuthor%5D&cauthor=true&cauthor_uid=25944722), [Singh M](https://www.ncbi.nlm.nih.gov/pubmed/?term=Singh%20M%5BAuthor%5D&cauthor=true&cauthor_uid=25944722) et al (2015) AMPA receptor activation causes preferential mitochondrial Ca²⁺ load and oxidative stress in motor neurons. *Brain Res* 1616: 1-9.  [Sánchez-Gómez MV](https://www.ncbi.nlm.nih.gov/pubmed/?term=S%C3%A1nchez-G%C3%B3mez%20MV%5BAuthor%5D&cauthor=true&cauthor_uid=21414921), [Alberdi E](https://www.ncbi.nlm.nih.gov/pubmed/?term=Alberdi%20E%5BAuthor%5D&cauthor=true&cauthor_uid=21414921), [Pérez-Navarro E](https://www.ncbi.nlm.nih.gov/pubmed/?term=P%C3%A9rez-Navarro%20E%5BAuthor%5D&cauthor=true&cauthor_uid=21414921) et al (2011) Bax and calpain mediate excitotoxic oligodendrocyte death induced by activation of both AMPA and kainate receptors. *J Neurosci* 31: 2996-3006. |

| **Phenytoin** |
| --- |
| **Mode of action** |
| Phenytoin protect against seizures by causing voltage-dependent block of [voltage gated sodium channels](https://en.wikipedia.org/wiki/Voltage_gated_sodium_channel). This blocks sustained high frequency repetitive firing of [action potentials](https://en.wikipedia.org/wiki/Action_potentials). This is accomplished by reducing the amplitude of sodium-dependent action potentials through enhancing steady state inactivation. Phenytoin binds preferentially to the inactive form of the sodium channel producing voltage-dependent, use-dependent and time-dependent block of sodium-dependent action potentials. |
| **Theoretical effects on mitochondrial function** |
| Phenytoin have been shown to decrease oxidative stress demonstrated by increase in glutathione reductase activity in patients receiving it . ROS formation was significantly raised when rat hepatocytes were treated with phenytoin. Phenytoin-reactive metabolites might affect the electron-transfer chain, ATP synthesis, and/or mitochondrial membrane. Consistent with these findings, mitochondrial membrane potential in hepatocyte treated with phenytoin collapsed .  Oxidative stress might also be involved in the hepatotoxicity induced by phenytoin. |
| **Effects measured/seen in (mitochondrial) patients, mitochondrial cell lines or normal cell lines** |
| Cognitive impairment is an important side effect in patients treated chronically with phenytoin. Some studies demonstrated in phenytoin-treated rats brain an increase in the oxidative stress parameters. The rat brain MDA and GSH levels were measured. MDA is an end product of free radical generation (Liu et al., 1997) and glutathione plays an important role in protecting cells against oxidative damage as a free radical scavenger . The rat brain MDA levels were significantly increased and the GSH levels were significantly reduced in the phenytoin-treated rats. Phenytoin has been reported to increase both DNA oxidation and homologous recombination in a concentration and time-dependent manner .  This mechanism could be responsible even of hepatotoxicity. |
| **References** |
| Liu L, Wells PG (1994) [In vivo phenytoin-initiated oxidative damage to proteins and lipids in murine maternal hepatic and embryonic tissue organelles: potential molecular targets of chemical teratogenesis.](https://www.ncbi.nlm.nih.gov/pubmed/8171432) *Toxicol Appl Pharmacol*. 125: 247-55.  Reeta KH, Mehla J, Gupta YK (2009) [Curcumin is protective against phenytoin-induced cognitive impairment and oxidative stress in rats.](https://www.ncbi.nlm.nih.gov/pubmed/19765566) *Brain Res*. 1301: 52-60.  Santos NAG, Medina WSG, Martins NM et al (2008) Aromatic antiepileptic drugs and mitochondrial toxicity: effects on mitochondria isolated from rat liver. *Toxicol In Vitro* 22:1143–52.  Santos NA, Medina WS, Martins NM et al (2008) [Involvement of oxidative stress in the hepatotoxicity induced by aromatic antiepileptic drugs.](https://www.ncbi.nlm.nih.gov/pubmed/18783732) *Toxicol In Vitro* 22: 1820-4.  Winn LM, Kim PM, Nickoloff JA (2003) Oxidative stress-induced homologous recombination as a novel mechanism for phenytoin-initiated toxicity. *J Pharmacol Exp Ther* 306: 523–37. |

| **Propofol** |
| --- |
| **Summary** |
| Propofol is the most widely used intravenous anaesthetic agent used in both elective and acute settings for children and adults. Findings from two case series of paediatric patients with mitochondrial disease that underwent muscle biopsy suggested that short duration of general anaesthesia (60 mins (median) (Footitt et al. 2008) and 36 mins (mean) (Driessen et al. 2007), respectively) was safe. Also, no complication was observed in the subgroup of patients who received propofol as both induction and maintenance agents. A case series of nine patients with the m.3243A>G-related mitochondrial disease (eight with MELAS phenotype) who underwent various procedures under GA showed that electrolyte imbalance was frequently observed post-operatively (n=7), and renal impairment was identified in two patients (Gurrieri et al. 2011). However, none of these complications was directly attributed to the use of propofol. Several case reports of patients with mitochondrial disease caused by different genotypes also suggested that propofol appeared to be a safe induction agent (Table ).  However, prolonged use of propofol should be avoided in both children and adults with mitochondrial disease, based on the findings extrapolated from the literature. Propofol infusion syndrome was first described in five paediatric cases (age range four weeks to 6 years) presenting with respiratory infection required mechanical ventilation (Parke et al. 1992). Propofol infusion was administered for over 60 hours in all cases. PRIS was subsequently reported in adult patients who had a severe head injury and been sedated with propofol. In the same study, the risk of propofol infusion syndrome was dose-dependent particularly when the mean dose went above 5mg/kg per hour (Cremer et al. 2001). In a large, multi-centre prospective study, PRIS was reported in approximately 1% of patients (11/1107), but the duration of infusion did not appear to be a significant predictor.(Roberts et al. 2009) Krajcova and colleagues performed the multivariate regression analysis using data derived from 128 reported cases of PRIS, and identified four key factors that were associated with increased mortality risk of PRIS: higher dose, longer duration of infusion, development of fever and the presence of traumatic brain injury (Krajcova et al. 2015) |
| **Mode of action** |
| Propofol acts on the GABA A receptors that exert the inhibitory effects in the central nervous system. It has also been shown to inhibits excitatory glutamate release by a presynaptic mechanism, most likely through an impact on voltage-gated sodium channels (Lingamaneni et al. 2001). Propofol is highly protein-bound and metabolised in the liver. The half-life of propofol is estimated to be between 2 to 24 hours. However, the clinical effects of single dose propofol wear off very rapidly, typically within a few minutes. |
| **Known effects on mitochondrial function** |
| Animal model study   - Branca et al. study on the isolated rat liver mitohcondria – dose dependent reduction of respiratory chain capacity (by decreasing the transmembrane electrical potential) (Branca et al. 1991). - Rigoulet et al. 1996 study on the isolated rat liver mitochondria – inhibition of complex I and modification of complex V (ATPase), causing the proton leak (Rigoulet et al. 1996). - Schenkman et al. 2000 Study on the hearts of 12 adult guinea pigs - Propofol impairs either oxygen utilization or inhibits electron flow along the mitochondrial electron transport chain in the guinea pig cardiomyocyte. Propofol also significantly decreases ventricular performance in the isolated perfused heart in a dose-depdent fashion (Schenkman and Yan 2000). - Quintana et al. 2012 Ndufs4 knock out (KO) mice showed hypersensitivity (2.5 – 3 folds) to the volatile anaesthetic agents (isoflurane and halothane) compared to the WT mice. The KO mice also were hypersensitive (2 folds) to propofol. The KO mice were more resistant to ketamine than the wild type (Quintana et al. 2012). - Vanlender et al. 2015 8 rats were ventilated and sedated with propofol for 20 hours. Sequential biopsy specimens were taken from liver and skeletal muscle and used for determination of respiratory chain activities and propofol concentration. Activities were also measured in skeletal muscle from a patient who died of propofol infusion syndrome. In rats, authors detected a decrease in complex II+III activity starting at low tissue concentration of propofol (20 to 25 µM), further declining at higher concentrations. When measured individually, the activities of complexes II and III remained normal. Skeletal muscle from one patient taken in the acute phase of propofol infusion syndrome also shows a selective decrease in complex II+III activity (z-score: -2.96). Propofol impedes the electron flow through the respiratory chain and coenzyme Q is the main site of interaction with propofol (Vanlander et al. 2015).   Human tissue study   - Propofol could impair fatty acid oxidation based on the acycarnitine profile in a patient that developed PRIS: Reduced mitochondrial entry of long-chain acylcarnitine esters due to inhibition of the transport protein (carnitine palmityl transferase 1, CPT1) (Wolff et al). - Cray et al. 1998 Muscle biopsy of an infant who developed PRIS showed reduced COX activity. Treated successfully with haemofiltration and plasmapheresis (Cray et al. 1998). - Mehta et al. 1999 A child developed propofol infusion syndrome after exposure to the infusion for 5 hours. Muscle biopsy showed reduced CIV biochemical activities 0.004 (0.014 – 0.034) (Mehta et al. 1999). |
| **Effects measured/seen in mitochondrial patients, mitochondrial cell lines or normal cell lines** |
| Several studies investigate side effects of drugs by measuring effects on mitochondrial function in healthy cell lines. Consider if the results of these kinds of studies are fully translatable to the mitochondrial patient, do these studies show which effects will be seen in mitochondrial patients.  Case series and case reports on the use of propofol in patients with mitochondrial disease is summarised in the table. |
| **References** |
| Bolton P, Peutrell J, Zuberi S, Robinson P (2003) Anaesthesia for an adolescent with mitochondrial encephalomyopathy-lactic acidosis-stroke-like episodes syndrome. *Paediatric anaesthesia* 13(5): 453-6.  Branca D, Roberti MS, Lorenzin P, Vincenti E, Scutari G (1991) Influence of the anesthetic 2,6-diisopropylphenol on the oxidative phosphorylation of isolated rat liver mitochondria. *Biochemical pharmacology* 42(1): 87-90.  Cray SH, Robinson BH, Cox PN (1998) Lactic acidemia and bradyarrhythmia in a child sedated with propofol. *Critical care medicine* 26(12): 2087-92.  Cremer OL, Moons KG, Bouman EA, Kruijswijk JE, de Smet AM, Kalkman CJ (2001) Long-term propofol infusion and cardiac failure in adult head-injured patients. *Lancet (London, England)* 357(9250): 117-8.  Driessen J, Willems S, Dercksen S, Giele J, van der Staak F, Smeitink J (2007) Anesthesia-related morbidity and mortality after surgery for muscle biopsy in children with mitochondrial defects. *Paediatric anaesthesia* 17(1): 16-21.  Ducharlet K, Thyagarajan D, Ierino F, McMahon LP, Lee D (2018) Perioperative risk assessment for successful kidney transplant in leigh syndrome: a case report. *BMC nephrology* 19(1): 23.  Footitt EJ, Sinha MD, Raiman JA, Dhawan A, Moganasundram S, Champion MP (2008) Mitochondrial disorders and general anaesthesia: a case series and review. *British journal of anaesthesia* 100(4): 436-41.  Guasch E, Civantos B, Aguilar JM, Torres MD, Gilsanz F (2003) Progressive external ophthalmoplegia and ambulatory remifentanil-propofol based anaesthesia. *Anaesthesia* 58(6): 607-8.  Gurrieri C, Kivela JE, Bojanic K, et al. (2011) Anesthetic considerations in mitochondrial encephalomyopathy, lactic acidosis, and stroke-like episodes syndrome: a case series. *Canadian journal of anaesthesia = Journal canadien d'anesthesie* 58(8): 751-63.  Krajcova A, Waldauf P, Andel M, Duska F (2015) Propofol infusion syndrome: a structured review of experimental studies and 153 published case reports. *Critical care (London, England)* 19: 398.  Lingamaneni R, Krasowski MD, Jenkins A, et al. (2001) Anesthetic properties of 4-iodopropofol: implications for mechanisms of anesthesia. *Anesthesiology* 94(6): 1050-7.  Mehta N, DeMunter C, Habibi P, Nadel S, Britto J (1999) Short-term propofol infusions in children. *Lancet (London, England)* 354(9181): 866-7.  Miyamoto Y, Miyashita T, Takaki S, Goto T (2016) Perioperative considerations in adult mitochondrial disease: A case series and a review of 111 cases. *Mitochondrion* 26: 26-32.  Parke TJ, Stevens JE, Rice AS, et al. (1992) Metabolic acidosis and fatal myocardial failure after propofol infusion in children: five case reports. *BMJ (Clinical research ed)* 305(6854): 613-6.  Quintana A, Morgan PG, Kruse SE, Palmiter RD, Sedensky MM (2012) Altered anesthetic sensitivity of mice lacking Ndufs4, a subunit of mitochondrial complex I. *PLoS One* 7(8): e42904.  Rigoulet M, Devin A, Averet N, Vandais B, Guerin B (1996) Mechanisms of inhibition and uncoupling of respiration in isolated rat liver mitochondria by the general anesthetic 2,6-diisopropylphenol. *European journal of biochemistry* 241(1): 280-5.  Roberts RJ, Barletta JF, Fong JJ, et al. (2009) Incidence of propofol-related infusion syndrome in critically ill adults: a prospective, multicenter study. *Critical care (London, England)* 13(5): R169.  Schenkman KA, Yan S (2000) Propofol impairment of mitochondrial respiration in isolated perfused guinea pig hearts determined by reflectance spectroscopy. *Critical care medicine* 28(1): 172-7.  Terkawi AS, Wani TM, Al-Shuaibi KM, Tobias JD (2012) Anesthetic considerations in Leigh disease: Case report and literature review. *Saudi journal of anaesthesia* 6(2): 181-5.  Vanlander AV, Okun JG, de Jaeger A, et al. (2015) Possible pathogenic mechanism of propofol infusion syndrome involves coenzyme q. *Anesthesiology* 122(2): 343-52.  Vilela H, Garcia-Fernandez J, Parodi E, Reinoso-Barbero F, Duran P, Gilsanz F (2005) Anesthetic management of a patient with MERRF syndrome. *Paediatric anaesthesia* 15(1): 77-9. |

| **Quetiapine** |
| --- |
| **Mode of action** |
| Quetiapine is a second-generation antipsychotic that has affinity for D2, 5-HT2A, H1, alpha 1 and 5-HT1A receptors. Its precise mechanism of action is unknown, but according to the dopamine theory of schizophrenia, antipsychotic effects might be related to the drug’s ability to reduce dopaminergic neurotransmission in the mesolimbic pathway. |
| **Theoretical effects on mitochondrial function** |
| Inhibition of MCI. |
| **Known effects on mitochondrial function** |
| Inhibition of MCI. |
| **Effects measured/seen in mitochondrial patients, mitochondrial cell lines or normal cell lines** |
| No reports in clinical practice/in vivo. Lymphoblastoid cell lines (schizophrenia patients); reduced oxygen consumption and respiratory control ratio. Rodents (mice) possible antioxidant capacity (brain tissue). PC12 cell cultures reduce BAX mediated proapoptotic molecules. Reduced MCI in isolated rat liver mitochondria. |
| **References** |
| Modica-Napolitano JS, Lagace CJ, Brennan WA et al (2003) Differential effects of typical and atypical neuroleptics on mitochondrial function in vitro. *Arch Pharm Res* 26: 951–9.  Scaini G, Quevedo J, Velligan D et al (2018) Second generation antipsychotic-induced mitochondrial alterations: Implications for increased risk of metabolic syndrome in patients with schizophrenia. *J Eur Coll* *Neuropsychopharmacol* 28: 369–80.  Wei Z, Mousseau DD, Richardson JS (2003) Atypical antipsychotics attenuate neurotoxicity of beta-amyloid(25-35) by modulating Bax and Bcl-X(l/s) expression and localization. *J Neurosci Res* 74: 942–7.  Xuan Y, Yan G, Wu R et al (2015) The cuprizone-induced changes in (1)H-MRS metabolites and oxidative parameters in C57BL/6 mouse brain: Effects of quetiapine. *Neurochem Int* 90: 185–92. |

| **Quinolones** |
| --- |
| **Mode of action** |
| Quinolones and fluoroquinolones are chemotherapeutic bactericidal drugs, eradicating bacteria by interfering with [DNA replication](https://en.wikipedia.org/wiki/DNA_replication). Quinolones inhibit the bacterial [DNA gyrase](https://en.wikipedia.org/wiki/DNA_gyrase) or the [topoisomerase IV](https://en.wikipedia.org/wiki/Topoisomerase_IV) enzyme, thereby inhibiting [DNA](https://en.wikipedia.org/wiki/DNA) replication and [transcription](https://en.wikipedia.org/wiki/Transcription_%28genetics%29). Topoisomerase II is also a target for a variety of quinolone-based drugs. High activity against the eukaryotic type II enzyme is exhibited by drugs containing aromatic substituents at their C-7 positions. First and second generation fluoroquinolones selectively inhibit the [topoisomerase II](https://en.wikipedia.org/wiki/Topoisomerase_II) ligase domain, leaving the two nuclease domains intact. This modification, coupled with the constant action of the [topoisomerase II](https://en.wikipedia.org/wiki/Topoisomerase_II) in the bacterial cell, leads to DNA fragmentation via the nucleasic activity of the intact enzyme domains. Third and fourth generation fluoroquinolones are more selective for the [topoisomerase IV](https://en.wikipedia.org/wiki/Topoisomerase_IV) ligase domain, and thus have enhanced gram-positive coverage. |
| **Theoretical effects on mitochondrial function** |
| Ciprofloxacin makes site-specific double-stranded DNA breaks and depletes mtDNA in eukaryotic cells and fluoroquinolones can decrease mtDNA-encoded protein levels and cause oxidative stress. There maybe genetic susceptibility to effects. |
| **Known effects on mitochondrial function** |
| Isolated studies from 1980s onwards suggest fluoroquinolones impair mitochondrial function, and may trigger oxidative stress in mitochondria, inhibiting their function across a range of mammalian cells, as well as in mice. However, at therapeutic concentrations, the fluoroquinolones shown to have very little effect on human DNA. Mitochondrial damage also not the only theory; study in human kidney cells showed fluoroquinolones can bind to iron atoms from the active sites of several enzymes that modify DNA, leading to epigenetic changes that might be related to some of the drugs’ side effects (Marchant, J. When antibiotics turn toxic. *Nature* 555, 431–433 2018). |
| **Effects measured/seen in mitochondrial patients, mitochondrial cell lines or normal cell lines** |
| No reports in clinical practice/in vivo. Oxidative stress (human Achilles tendon cells), up-regulation of antioxidant genes (mouse tissue), mtDNA depletion and mtDNA DSBs (mammalian cells), reduced mtDNA-encoded protein levels. No studies in mitochondrial disease patients or cell lines. |
| **References** |
| Aldred KJ, Schwanz HA, Li G et al (2013) Overcoming target-mediated quinolone resistance in topoisomerase IV by introducing metal-ion-independent drug-enzyme interactions. *ACS Chem Biol* 8: 2660–8.  Badal S, Her YF, Maher LJ (2015) Nonantibiotic Effects of Fluoroquinolones in Mammalian Cells. *J Biol Chem* 290: 22287–97.  Kalghatgi S, Spina CS, Costello JC et al (2013) Bactericidal antibiotics induce mitochondrial dysfunction and oxidative damage in Mammalian cells. *Sci Transl Med* 5: 192ra85.  Lawrence JW, Claire DC, Weissig V et al (1996) Delayed cytotoxicity and cleavage of mitochondrial DNA in ciprofloxacin-treated mammalian cells. *Mol Pharmacol* 50: 1178–88.  Lowes DA, Wallace C, Murphy MP et al (2009) The mitochondria targeted antioxidant MitoQ protects against fluoroquinolone-induced oxidative stress and mitochondrial membrane damage in human Achilles tendon cells. *Free Radic Res* 43: 323–8.  Marchant J (2018) When antibiotics turn toxic. *Nature* 555: 431–3.  Nadanaciva S, Dillman K, Gebhard DF et al (2010) High-content screening for compounds that affect mtDNA-encoded protein levels in eukaryotic cells. *J Biomol Screen* 15: 937–48.  Nadanaciva S, Murray J, Wilson C et al (2011) High-throughput assays for assessing mitochondrial dysfunction caused by compounds that impair mtDNA-encoded protein levels in eukaryotic cells. *Curr Protoc Toxicol* Chapter 3: Unit 3.11.  Wang X, Zhao X, Malik M et al (2010) Contribution of reactive oxygen species to pathways of quinolone-mediated bacterial cell death. *J Antimicrob Chemother* 65: 520–4. |

| **Barbiturates** |
| --- |
| **Mode of action** |
| Risperidone is one of the second generation antipsychotics (also known as atypical antipsychotics) medications. It inhibits dopaminergic D2 receptors and serotonergic 5-HT2A, 2C receptors, and is an agonist to 5-HT1A receptors in the brain (Horacek et al. 2006). |
| **Known effects on mitochondrial function** |
| Animal study  Modica-Napolitano et al. 2003 studied the effects of various antipsychotic medications including risperidone on rat liver mitochondria (Modica-Napolitano et al. 2003):   1. Polarographic measurement of oxygen consumption in freshly isolated mitochondria showed that electron transfer activity at respiratory complex I is inhibited by risperidone; 2. Risperidone also inhibits the NADH-coenzyme Q reductase in freeze-thawed mitochondria (a direct measure of complex I activity); 3. The inhibition of NADH-coenzyme Q reductase activity by the atypicals risperidone and quetiapine was 2-4 fold less than that for the typical neuroleptics. Clozapine and olanzapine had only slight effects on NADH-coenzyme Q reductase activity   Eftekhari et al. 2016 performed two studies on the hepatotoxic effects of risperidone (Eftekhari et al. 2016):   1. In vitro study: Isolated rat hepatocytes were incubated with risperidone and the authors showed that could induce cytotoxicity via rising reactive oxygen species (ROS), mitochondrial potential collapse, lysosomal membrane leakiness, GSH depletion and lipid peroxidation. 2. In vivo study: Liver function enzyme test and histopathological evaluation confirmed RIS-(6 mg/kg) induced damage   Pillai et al. 2007 studied on the long term effect (up to 180 days) of various typicaland atypical anti-psychotic medications (including risperidone) on the expression of the key antioxidant defense enzymes (manganese, MnSOD present in mitochondria) and lipid peroxidation products in rat brain (Pillai et al. 2007):   1. Only the rats treated with haloperidol showed a reduction in MnSOD level after 90 days of treatment; 2. The reduction of MnSOD activity was observed in risperidone and all other anti-psychotics medication after 180 days of treatment; 3. Switching haloperidol to other atypical antipsychotics medication restored the antioxidant enzymes.   Human tissue  Casademont et al. 2007 studied the effects of antipsychotics medications in three groups of patients with schizophrenia (treatment naïve, on typical neuroleptics, and atypical neuroleptics including risperidone, n=21) (Casademont et al. 2007). Absolute enzyme activities of ETC complexes I to IV in peripheral mononuclear cells were spectrophotometrically quantified, and oxygen consumption with substrates of different complexes was measured polarographically. Reduced complex I activity was identified, and most significant change was observed in those treated with typical neuroleptics medications. |
| **Effects measured/seen in mitochondrial patients, mitochondrial cell lines or normal cell lines** |
| Effects measured / seen in mitochondrial patient, mito cell lines or normal cell lines. Several studies investigate side effects of drugs by measuring effects on mitochondrial function in healthy cell lines. Consider if the results of these kinds of studies are fully translatable to the mitochondrial patient, do these studies show which effects will be seen in mitochondrial patients. There is no specific report commenting the safety and efficacy of risperidone in patients with mitochondrial disease, based on my literature search. |
| **References** |
| Casademont J, Garrabou G, Miro O et al (2007) Neuroleptic treatment effect on mitochondrial electron transport chain: peripheral blood mononuclear cells analysis in psychotic patients. *Journal of clinical psychopharmacology* 27(3): 284-8.  Eftekhari A, Ahmadian E, Azarmi Y et al (2016) In vitro/vivo studies towards mechanisms of risperidone-induced oxidative stress and the protective role of coenzyme Q10 and N-acetylcysteine. *Toxicology mechanisms and methods* 26: 520-8.  Horacek J, Bubenikova-Valesova V, Kopecek M et al (2006) Mechanism of Action of Atypical Antipsychotic Drugs and the Neurobiology of Schizophrenia. *CNS Drugs* 20: 389-409.  Modica-Napolitano JS, Lagace CJ, Brennan WA (2003) Differential effects of typical and atypical neuroleptics on mitochondrial function in vitro. *Archives of pharmacal research* 26: 951-9.  Pillai A, Parikh V, Terry AV et al (2007) Long-term antipsychotic treatments and crossover studies in rats: Differential effects of typical and atypical agents on the expression of antioxidant enzymes and membrane lipid peroxidation in rat brain. *Journal of Psychiatric Research* 41(5): 372-86. |

| **Rocuronium** |
| --- |
| **Mode of action** |
| Rocuronium is a non-depolarising neuromuscular blocking drug (post synaptic nicotinic receptors) used in general anaesthesia for endotracheal intubation and surgery. It has a rapid onset of action, with a clinical duration of 10-12 minutes (considered as intermediate duration, compared to suxamethonium) (Hunter 1996). The effect of neuromuscular blockade can be reversed by either neostigmine or sugammadex. |
| **Theoretical effects on mitochondrial function** |
| No literature suggesting rocuronium can cause direct toxicity to mitochondria. |
| **Effects measured/seen in mitochondrial patients, mitochondrial cell lines or normal cell lines** |
| Sharma et al. 2001 reported a 2 year old child requiring repair of a sinus venosus atrial septal defect with known diagnoses of Prader-Willi syndrome and mitochondrial myopathy (complex I and IV deficiencies) (Sharma et al 2001). The patient was given intramuscular ketamine 50mg for the insertion of external jugular venous access. The GA induction agents were propofol 20mg and fentanyl 20 microgram. She received a single dose of rocuronium for muscle relaxation. The anaesthesia was maintained with propofol. She was extubated uneventfully two hours after the surgery.  Saetteke et al. reported a 4-week old, 3.7kg infant underwent muscle biopsy for suspected mitochondrial disease under GA (Saettele et al 2013). Clinically, the infant had hypotonia, failure to thrive, lactic acidosis and abnormal signal abnormalities in bilateral thalami, basal ganglia and frontal white matter. The induction agents were propofol, fentanyl and rocuronium. Propofol was used for maintenance of anaesthesia. She remained intubated and ventilated for respiratory and metabolic and respiratory acidosis post-operatively. She deteriorated with worsening metabolic acidosis and developed bradycardia 72 hours later. She died 2 weeks after admission following the withdrawal of mechanical ventilation. Muscle biopsy findings were consistent with mitochondrial disease (but no detail provided).  Ciccotelli et al. 1997 reported a 41 year old with a diagnosis of NARP underwent Nissen fundoplication with parietal cell vagotomy and cholecystectomy. Because of an anticipated difficult intubation (Mallampati class 3) and severe gastroesophageal reflux, his trachea was intubated using fiberoptic guidance with topical lidocaine anesthesia of the oropharynx and transmucosal block of the superior laryngeal nerves after the patient had been sedated with midazolam, fentanyl, and droperidol. He then underwent an uneventful general anesthetic with thiopental, fentanyl, rocuronium, and nitrous oxide 70% in oxygen. At the end of surgery, neuromuscular blockade was reversed easily within 5 min with neostigmine and glycopyrrolate. Extubated promptly on resumption of spontaneous ventilation and responsiveness to verbal commands (Ciccotelli et al 1997).  Calzavacca et al. 2011 reported a 40 year man affected by Kearns-Sayre syndrome who underwent an elective laparoscopic cholecystectomy. Pre-operative assessment showed normal neurological examination, trifascicular heart block on ECG, normal echocardiogram and normal respiratory function. The GA induction was achieved with propofol, fentanyl and rocuronium. The GA was maintained with sevoflurane (maximum 1.6%) and N_2_O/O_2_ mixture 60/40. The patient was extubated uneventfully 60 minutes later and no reversal of neuromuscular block was necessary (Calzavacca et al 2011). |
| **References** |
| Calzavacca P, Schmidt W, Guzzi M (2011) General anaesthesia for laparoscopic cholecystectomy in a patient with the kearns-sayre syndrome. *Case Rep Anesthesiol.*  Ciccotelli KK, Prak EL, Muravchick S (1997) An adult with inherited mitochondrial encephalomyopathy: report of a case. *Anesth* 87: 1240-2.Hunter JM. (1996) Rocuronium: the newest aminosteroid neuromuscular blocking drug. *B J Anaesth.* 76: 481-3.  Hunter JM. Rocuronium: the newest aminosteroid neuromuscular blocking drug. *British journal of anaesthesia.* Apr 1996;76(4):481-483.  Saettele AK, Sharma A, Murray DJ (2013) Case scenario: Hypotonia in infancy: anesthetic dilemma. *Anesth* 119: 443-6.  Sharma AD, Erb T, Schulman SR et al (2001) Anaesthetic considerations for a child with combined Prader–Willi syndrome and mitochondrial myopathy. *Pediatr Anaesth.* 11: 488-90. |

| **Salicylates** |
| --- |
| **Mode of action** |
| Salicylates are irreversible cyclooxygenase (COX) inhibitors, which at low doses (75 to 81 mg/day) do inhibit COX-1. This effect inhibits platelet generation of thromboxane A2, resulting in an antithrombotic effect. At higher doses (650 mg to 4 g/day) salicylic acid also inhibits COX-2, which blocks prostaglandin production, and has analgesic and antipyretic effects. At higher (near) toxic concentrations (between 4 and 8 g/day) salicylic acid has an anti-inflammatory effect in rheumatic disorders, which may include both prostaglandin-dependent and -independent effects. |
| **Theoretical effects on mitochondrial function** |
| The inhibitory effects of salicylic acid on mitochondrial function is mainly attributed to its actions as a protonophore causing uncoupling of the mitochondrial membrane potential (Nulton-Persson *et al*, 2004). In addition, a wide variety of enzymes, including dehydrogenases, decarboxylases, and aminotransferases, can be inhibited by salicylic acid *in vitro* (Nulton-Persson *et al*, 2004). |
| **Known effects on mitochondrial function** |
| *Clinically*, a mixed respiratory alkalosis and metabolic acidosis is the most frequently observed hallmark associated with mitochondrial dysfunction seen with high-dose salicylic acid toxicity in adults (Pham et al, 2015). However, in children salicylates produce a high anion gap acidosis. Metabolic acidosis occurs during salicylate toxicity due to uncoupling of oxidative phosphorylation and interference with the Krebs cycle (Pham et al, 2015; DuBose, 2007; Kreisberg, 1983), resulting in accumulation of lactic acid and ketoacids in as many as 40% of adult patients with salicylate poisoning (Arena *et al*, 1978; Proudfoot *et al*, 2003). The anion gap is mainly composed of ketoanions and lactate, while salicylate anion seldomly exceeds 3 mEq/L.  Mechanistically, *in vitro studies* have demonstrated  that in isolated liver and kidney mitochondria, salicylic acid acts as an uncoupler of oxidative phosphorylation (Brody & Fouts, 1956; Whitehouse, 1964; Adams & Cobb, 1958; Mehlman & Tobin, 1972; Poćwiardowska, 1976; Haas *et al*, 1985; Tomoda *et al*, 1992; Braun *et al*, 2012) and can inhibit ADP-dependent mitochondrial respiration (Adams & Cobb, 1958; Mehlman & Tobin, 1972; Poćwiardowska, 1976; Keller *et al*, 1992; Tomoda *et al*, 1992; Martens & Lee, 1984) Although a wide variety of enzymes, including dehydrogenases like the competitive inhibition of α-ketoglutarate dehydrogenase by salicylates was observed in isolated cardiomyocyte mitochondria (Nulton-Persson *et al*, 2004)). In addition, salicylic acid inhibits various decarboxylases, and aminotransferases *in vitro* (Smith & Dawkins, 1971), and has been shown to inhibit mitochondrial β-oxidation (Fromenty & Pessayre, 1995) and to induce PTP opening (Trost & Lemasters, 1996). More recently, *in vitro* and *in vivo* (zebrafish larvae) studies demonstrated salicylic acid-induced inhibition of the ferrochelatase-catalyzed heme biosynthesis to be associated with salicylic acid-induced mitochondrial dysfunction (Gupta *et al*, 2013). |
| **Effects measured/seen in mitochondrial patients, mitochondrial cell lines or normal cell lines** |
| Case report, a 10-year old girl with MELAS (Fryer et al, 2016). Three weeks before presentation, daily aspirin therapy was started. Eleven days later, she developed headache, nausea, emesis, blurred vision, and slurred speech. In addition, she suffered from acidosis (blood pH 7.33) and high blood lactate levels (5.5 mmol/L). The dose and indication for asprin use are, however, not provided in this case report. |
| **References** |
| Adams SS, Cobb R (1958) A possible basis for the anti-inflammatory activity of salicylates and other non-hormonal anti-rheumatic drugs. *Nat*, 181: 773.  Arena FP, Dugowson C, Saudek CD (1978) Salicylate-induced hypoglycemia and ketoacidosis in a nondiabetic adult. *Arch Int Med* 138: 1153-4.  Braun FK, Al-Yacoub N, Plötz M et al (2012) Nonsteroidal anti-inflammatory drugs induce apoptosis in cutaneous T-cell lymphoma cells and enhance their sensitivity for TNF-related apoptosis-inducing ligand. *J Invest Dermatol* 132: 429-39.  Brody TM, Fouts W (1956) Action of sodium salicylate and related compounds on tissue metabolism in vitro. *J Pharmacol Exp Ther* 117: 39-51.  DuBose TD (2007) Disorder of Acid-Base Balance. *Brenner and Rector's The Kidney 8th ed.* Philadelphia: Saunders 505–46.  Fromenty B, Pessayre D (1995) Inhibition of mitochondrial beta-oxidation as a mechanism of hepatotoxicity. *Pharmacol Ther* 67: 101-54.  Fryer RH, Bain JM, Darryl C (2016) Mitochondrial encephalomyopathy lactic acidosis and stroke-like episodes (MELAS): a case report and critical reappraisal of treatment options. *Pediatr Neurol* 56: 59-61.  Gupta V, Liu S, Ando H et al (2013) Salicylic acid induces mitochondrial injury by inhibiting ferrochelatase heme biosynthesis activity. *Mol Pharmacol*, mol-113.  Haas R, Parker Jr WD, Stumpf D, Eguren LA (1985) Salicylate-induced loose coupling: protonmotive force measurements. *Biochem Pharmacol* 34: 900-2.  Keller BJ, Yamanaka H, Thurman RG (1992) Inhibition of mitochondrial respiration and oxygen-dependent hepatotoxicity by six structurally dissimilar peroxisomal proliferating agents. *Toxicology* 71: 49-61.  Kreisberg RA, Wood BC (1983) Drug and chemical-induced metabolic acidosis. *Best Pract Res Clin Endocrinol Metab* 12: 391-411.  Martens ME, Lee CP (1984) Reye's syndrome: salicylates and mitochondrial functions. *Biochem Pharmacol* 33: 2869-76.  Mehlman MA, Tobin RB (1972) Oxidative phosphorylation and respiration by rat liver mitochondria from aspirin-treated rats. *Biochem Pharmacol* 21: 3279-85.  Mingatto FE, Santos AC, Uyemura SA et al (1996) In Vitro Interaction of Nonsteroidal Anti-inflammatory Drugs on Oxidative Phosphorylation of Rat Kidney Mitochondria: Respiration and ATP Synthesis. *Arch Biochem Biophys* 334: 303-8.  Nulton-Persson AC, Szweda LI, Sadek HA (2004) Inhibition of cardiac mitochondrial respiration by salicylic acid and acetylsalicylate. *J Cardiovasc Pharmacol* 44: 591-5.  Pham AQT, Xu LHR, Moe OW (2015) Drug-induced metabolic acidosis. *F1000Research*, 4.  Poćwiardowska E (1976) The effects of antipyretics on metabolic processes in rat liver mitochondria. Part II. The action of sodium salicylate, and pyrazolones on oxidation of alpha-ketoglutarate. *Pol J Pharmacol Pharm* 28: 227-31.  Proudfoot AT, Krenzelok EP, Brent J et al (2003) Does urine alkalinization increase salicylate elimination? If so, why? *Toxicol Rev* 22: 129-36.  Smith MJH, Dawkins PD (1971) Salicylate and enzymes. *J Pharm Pharmacol* 23: 729-44.  Tomoda T, Takeda K, Kurashige T et al (1992) Experimental study on Reye's syndrome: Inhibitory effect of interferon alfa on acetylsalicylate-induced injury to rat liver mitochondria. *Metabolism* 41: 887-92.  Trost LC, Lemasters JJ (1996) The mitochondrial permeability transition: a new pathophysiological mechanism for Reye's syndrome and toxic liver injury. *J Pharmacol Exp Ther* 278: 1000-5.  Whitehouse MW (1964) Biochemical properties of anti-inflammatory drugs—III.: Uncoupling of oxidative phosphorylation in a connective tissue (cartilage) and liver mitochondria by salicylate analogues: Relationship of structure to activity. *Biochem Pharmacol* 13: 319-36. |

| **Tetracyclines** |
| --- |
| **Mode of action** |
| Class of antibiotics that targets bacterial translation - inhibit protein synthesis by preventing the attachment of aminoacyl-tRNA to the ribosomal acceptor (A) site. |
| **Theoretical effects on mitochondrial function** |
| Work in the 1960s described that tetracyclines inhibit translation of proteins encoded by mtDNA but not by nuclear DNA.  Tetracyclines inhibit mitochondrial beta-oxidation.  Minocycline has a cytoprotective effect following ischaemia-reperfusion injury (prevents apoptosis). |
| **Effects measured/seen in mitochondrial patients, mitochondrial cell lines or normal cell lines** |
| \| **Type of Study** \| **Model Used** \| **Effect Measured** \| **Effect seen/ Mechanism** \| **Ref** \| \| --- \| --- \| --- \| --- \| --- \| \| **In vitro and in vivo** \| Mouse liver and human liver mitochondria;  Live mouse \| Effect of tetracycline on palmitic acid oxidation by mouse liver and human liver mitochondria \| addition of tetracycline 0.25, 0.5, 1 or 2 mM inhibited by 15, 38, 56 and 65%, respectively, the formation of beta-oxidation products; even bigger effect with human liver mitochondria; in vivo, tetracycline, 0.25 or 1 mmole per kg, inhibited by 53 and 84%, respectively, the exhalation of [14C]CO2 during the first 3 hours following the administration of a tracer dose of [U-14C]palmitic acid. Increased hepatic triglycerides and liver histology showed microvesicular steatosis at 6 and 24 hr. \| Frenaux et al 1988 \| \| **In vitro** \| Primary hepatocyte cultures prepared from male beagle dog liver \| specific increase in intracellular triglyceride following a 24-h exposure to noncytotoxic levels of tetracycline \| inhibition of mitochondrial lipid metabolism is primary mechanism leading to steatosis in dog hepatocytes following tetracycline exposure \| Amacher and Martin 1997 \| \| **In vitro** \| 62 rat intact hearts exposed to ischaemia/reperfusion and cell cultures of neonatal and adult rat ventricular myocytes \| Minocycline significantly reduced necrotic and apoptotic cell death, both in neonatal and adult myocytes, not only when given prior to hypoxia (p < 0.001), but also at reoxygenation (p < 0.05) \| Minocycline inhibits caspase activation and reactivation, increases the ratio of XIAP to smac/DIABLO, and reduces the mitochondrial leakage of cytochrome C and smac/DIABLO \| Scarabelli et al 2004 \| \| **In vitro and vivo** \| Cell lines;  Worms and flies \| Doxycycline disturbed mitochondrial proteostasis and function and disrupted global cellular transcriptional  Profiles in cell lines, and  impairs development and mitochondrial  function in worms and flies \| Tetracyclines induce a mitonuclear protein imbalance through their effects on mitochondrial translation - Even at low concentrations, tetracyclines induce mitochondrial proteotoxic stress, leading to changes in nuclear gene expression and altered mitochondrial dynamics and function \| Moullan et al 2015 \|   **Known effects in human patients with mitochondrial disease**:   \| **Type of Study** \| **Model Used** \| **Effect Measured** \| **Effect seen/ Mechanism** \| **Ref** \| \| --- \| --- \| --- \| --- \| --- \| \| **In vivo** \| 47y woman with CPEO \| Tetracycline 500 mg daily - One year later horizontal eye  movements improved above baseline level to 140% for the left eye and 120% for the right eye \| Authors postulated that improvement in ocular motility that we documented could have occurred from tetracycline blocking apoptosis through inhibition of cytochrome c and caspase-3 release in the mitochondria \| Omar and Johnson 2007 \| \| **In vivo** double-blind randomized pilot study \| 16 patients with PEO \| double-blind randomized pilot study (followed by an adjunctive open-label phase) was to evaluate whether tetracycline (500 mg/day × 14 days/month × 3 months) \| results do not formally support any effect of tetracycline on eye motility in PEO \| Mancuso et al 2011 \| |
| **References** |
| Amacher DE, Martin BA (1997) Tetracycline-induced steatosis in primary canine hepatocyte cultures. *Fundam Appl Toxicol.* 40: 256-63.  Fréneaux E, Labbe G, Letteron P et al (1988) Inhibition of the mitochondrial oxidation of fatty acids by tetracycline in mice and in man: possible role in microvesicular steatosis induced by this antibiotic. *Hepatology.* 8: 1056-62.  Mancuso M, Orsucci D, Calsolaro V et al (2011) Tetracycline treatment in patients with progressive external ophthalmoplegia. *Acta Neurol Scand*. 124: 417-23.  Moullan N, Mouchiroud L, Wang X et al (2015) Tetracyclines Disturb Mitochondrial Function across Eukaryotic Models: A Call for Caution in Biomedical Research. *Cell Rep* 10: 1681-1691.  Omar A, Johnson LN (2007) Tetracycline delays ocular motility decline in chronic progressive external ophthalmoplegia. *Neurology.* 68: 1159-60.  Scarabelli TM, Stephanou A, Pasini E et al (2004) Minocycline inhibits caspase activation and reactivation, increases the ratio of XIAP to smac/DIABLO, and reduces the mitochondrial leakage of cytochrome C and smac/DIABLO. J *Am Coll Cardiol*. 43: 865-74. |

| **Topiramate** |
| --- |
| **Mode of action** |
| The drug has five recognized actions:  1) sodium channel blocker  2) potentiates gamma-aminobutyric acid  3) glutamate antagonist  4) inhibits isoenzymes of carbonic anhydrase  5) high-voltage-activated calcium channels  There is evidence that topiramate may alter the activity of its targets by modifying their phosphorylation state instead of by a direct action. The effect on sodium channels could be of particular relevance for seizure protection. Although topiramate does inhibit high-voltage-activated calcium channels, the relevance to clinical activity is uncertain. Effects on specific GABA-A receptor isoforms could also contribute to the antiseizure activity of the drug. Topiramate selectively inhibits cytosolic (type II) and membrane associated (type IV) forms of carbonic anhydrase. The action on carbonic anhydrase isoenzymes may contribute to the drug’s side-effects, including its propensity to cause metabolic acidosis and calcium phosphate kidney stones. |
| **Theoretical effects on mitochondrial function** |
| Inhibiting high-voltage-activated calcium channels, topiramate may protect mitochondria against an high intracellular content of calcium; some literature data suggest that mitochondrial Ca2+ can stimulate and control the rate of oxidative phosphorylation, induce the mitochondrial permeability transition (MPT) and perhaps apoptotic cell death, and modify the shape of cytosolic Ca2+ pulses or transients. On the other hand, topiramate, inhibiting carbonic anhydrase, could induce metabolic acidosis; the development of acidosis with topiramate may be influenced by carbonic anhydrase polymorphisms. |
| **Known effects on mitochondrial function** |
| Topiramate is an inhibitor of the mitochondrial zinc enzyme human carbonic anhydrase when using the dansylamide competition-binding assay; according to other studies, however, topiramate is only a moderate inhibitor of carboanhydrase isoenzymes. On the other hand, when studying topiramate in the pilocarpine rat model of epilepsy, it has been shown that topiramate has a neuroprotective effect by inhibiting the mitochondrial transition pore. |
| **Effects measured/seen in mitochondrial patients, mitochondrial cell lines or normal cell lines** |
| Topiramate inhibits isoenzymes of carbonic anhydrase, inducing a type 2 renal tubule acidosis (RTA). The distal tubule of the nephron is unable to reclaim filtered bicarbonate, allowing increased urinary excretion, resulting in lowered serum bicarbonate levels. Although typically asymptomatic, the risk of clinically significant acidosis is increased with higher drug dosing or when the dosage is increased. When the drug is discontinued, the RTA resolves and the serum bicarbonate returns to baseline. Treatment includes discontinuing the drug and supportive care including intravenous fluids and sodium bicarbonate.  Very recent data, however, indicate that non ambulatory and neurologically impaired children are at high risk of developing kidney stones with topiramate. In children affected by chronic kidney disease an impaired renal tubular acidification but without overt metabolic acidosis (bicarbonate 18.0mmol/L or more) causes a tendency towards short stature that improves on long term alkali therapy. Hence propensity for kidney stone formation and somatic growth retardation deserve consideration in pediatric patients on long-term treatment with topiramate.  Some study showed a neuroprotective role of topiramante, for example in models of focal cerebral ischemia and epilepsy; the neuroprotective role of topiramate seems to be related to its inhibitory effect on the mitochondrial permeability transition pore. In vitro investigation of topiramate on respiration of rat hippocampal slices ruled out any direct effects on mitochondrial oxidative phosphorylation.  No study exist on mito cell lines or published data on mito patients. |
| **References** |
| Belotti EA, Taddeo I, Ragazzi M et al (2009) Chronic impact of topiramate on acid-base balance and potassium in childhood. *Eur J Paediatr Neurol*. 14: 445-8.  Dodgson SJ, Shank RP, Maryanoff BE et al (2000) Topiramate as an inhibitor of carbonic anhydrase isoenzymes. *Epilepsia.* 41 Suppl 1: S35-9.  Goyal M, Grossberg RI, O’Riordan MA et al (2009) Urolithiasis with topiramate in nonambulatory children and young adults. *Pediatr Neurol* 40: 289-94.  Kudin AP, Debska-Vielhaber G, Vielhaber S et al (2004) The mechanism of neuroprotection by topiramate in an animal model of epilepsy. *Epilepsia*. 45: 1478-87.  Mirza NS, Alfirevic A, Jorgensen A et al (2011) Metabolic acidosis with topiramate and zonisamide: an assessment of its severity and predictors. *Pharmacogenet Genomics*. 21: 297-302.  Mirza N, Marson AG, Pirmohamed M (2009) Effect of topiramate on acid-base balance: extent, mechanism and effects. *Br J Clin Pharmacol.* 68: 655-61.  Patrick P, Price TO, Diogo AL et al (2015) Topiramate Protects Pericytes from Glucotoxicity: Role for Mitochondrial CA VA in Cerebromicrovascular Disease in Diabetes. *J Endocrinol Diabetes*. 2.  Shiber JR. (2010) Severe non-anion gap metabolic acidosis induced by topiramate: a case report. *J Emerg Med*. 38: 494-6.  Takeoka M, Holmes GL, Thiele E (2001) Topiramate and metabolic acidosis in pediatric epilepsy. *Epilepsia*. 42: 387-92.  Yorns WR Jr, Hardison HH (2013) Mitochondrial dysfunction in migraine. *Semin Pediatr Neurol*. 20: 188-93. |

| **Valproic acid** |
| --- |
| **Mode of action** |
| It’s anticonvulsant effect has been attributed to the blockade of [voltage-gated sodium channels](https://en.wikipedia.org/wiki/Voltage-gated_sodium_channel) and increased brain levels of [gamma-aminobutyric acid](https://en.wikipedia.org/wiki/Gamma-Aminobutyric_acid)(GABA) The GABAergic effect is also believed to contribute towards the anti-manic properties of valproate.  In animals, sodium valproate raises cerebral and cerebellar levels of the inhibitory synaptic neurotransmitter, GABA, possibly by inhibiting GABA degradative enzymes, such as [GABA transaminase](https://en.wikipedia.org/wiki/GABA_transaminase), [succinate-semialdehyde dehydrogenase](https://en.wikipedia.org/wiki/Succinate-semialdehyde_dehydrogenase) and by inhibiting the re-uptake of GABA by neuronal cells. Prevention of neurotransmitter-induced hyperexcitability of nerve cells, via [Kv7.2 channel](https://en.wikipedia.org/wiki/KvLQT2) and [AKAP5](https://en.wikipedia.org/wiki/AKAP5), may also contribute to its mechanism. Also, it has been shown to protect against a seizure-induced reduction in [phosphatidylinositol (3,4,5)-trisphosphate](https://en.wikipedia.org/wiki/Phosphatidylinositol_(3,4,5)-trisphosphate) (PIP3) as a potential therapeutic mechanism.  An important aspect is the it also has [histone deacetylase-inhibiting effects](https://en.wikipedia.org/wiki/Histone_deacetylase_inhibitor). The inhibition of histone deacetylase, by promoting more transcriptionally active chromatin structures, likely presents the epigenetic mechanism for regulation of many of the neuroprotective effects attributed to valproic acid. Intermediate molecules mediating these effects include [VEGF](https://en.wikipedia.org/wiki/VEGF), [BDNF](https://en.wikipedia.org/wiki/BDNF), and [GDNF](https://en.wikipedia.org/wiki/GDNF). |
| **Theoretical effects on mitochondrial function** |
| Using multi”omics Study” wasdemonstrated that upon transient methylation changes of mitochondrial genes during VPA-treatment, increasing complexities of gene-interaction networks across time were demonstrated, which normalized during washout. In relation to persistently decreased ATP production, authors observed a decreased expression of mitochondrial complex I and III–V genes. Persistent transcripts and proteins were related to citric acid cycle and β-oxidation. |
| **Known effects on mitochondrial function** |
| VPA caused decrease in oxygen consumption rates and mitochondrial membrane potential. VPA exposure led to depleted ATP levels in HepG2 cells incubated in galactose medium suggesting dysfunction in mitochondrial ATP production. In addition, VPA exposure increased levels of mitochondrial reactive oxygen species (ROS), but adversely decreased protein levels of mitochondrial superoxide dismutase SOD2, suggesting oxidative stress caused by impaired elimination of mitochondrial ROS .Increased cell death and decrease in cell number was detected under both metabolic conditions.  VPA seems to interfere with mitochondrial biogenesis. In cells form POLG patients VPA administration caused a significant increase in the expression of POLG and several regulators of mitochondrial biogenesis. It was further supported by elevated mtDNA copy numbers. The effect of VPA on mitochondrial biogenesis was observed in both control and patient cell lines, but the capacity of mutant *POLG* to increase the expression of mitochondrial genes and to increase mtDNA copy numbers was less effective. |
| **Effects measured/seen in mitochondrial patients, mitochondrial cell lines or normal cell lines** |
| The most common effect of valproate in mitochondrial patients, particular in patients with *POLG* mutations, is a severe hepatotoxicity. |
| **References** |
| Fromenty B, Pessayre D (1995) [Inhibition of mitochondrial beta-oxidation as a mechanism of hepatotoxicity.](https://www.ncbi.nlm.nih.gov/pubmed/7494860) *Pharmacol Ther* 67: 101-54.  Jafarian I, Eskandari MR, Mashayekhi V et al (2013) [Toxicity of valproic acid in isolated rat liver mitochondria.](https://www.ncbi.nlm.nih.gov/pubmed/23819490) *Toxicol Mech Methods* 23: 617-23.  Komulainen T, Lodge T, Hinttala R (2015) [Sodium valproate induces mitochondrial respiration dysfunction in HepG2 in vitro cell model.](https://www.ncbi.nlm.nih.gov/pubmed/25745980) *J Toxicol* 331: 47-56.  Li S, Guo J, Ying Z et al (2015) V[alproic acid-induced hepatotoxicity in Alpers syndrome is associated with mitochondrial permeability transition pore opening-dependent apoptotic sensitivity in an induced pluripotent stem cell model.](https://www.ncbi.nlm.nih.gov/pubmed/25605636) *Hepatology* 61: 1730-9.  Nazıroğlu M, Yürekli VA (2013) [Effects of antiepileptic drugs on antioxidant and oxidant molecular pathways: focus on trace elements.](https://www.ncbi.nlm.nih.gov/pubmed/23584684) *Cell Mol Neurobiol* 33: 589-99.  Sitarz KS, Elliott HR, Karaman BS et al (2014) [Valproic acid triggers increased mitochondrial biogenesis in POLG-deficient fibroblasts.](https://www.ncbi.nlm.nih.gov/pubmed/24725338) *Mol Genet Metab* 112: 57-63.  Woltersa JEJ, Van Bredaa SGJ, Grossmann J (2018) Integrated ‘omics analysis reveals new drug-induced mitochondrial perturbations in human hepatocytes. *Toxicol Lett* 289: 1–13.  Yao KW, Mao LF, Luo MJ et al (1994) [The relationship between mitochondrial activation and toxicity of some substituted carboxylic acids.](https://www.ncbi.nlm.nih.gov/pubmed/8168171) *Chem Biol Interact* 90: 225-34. |

| **Volatile anesthetics** |
| --- |
| **Mode of action** |
| Volatile anesthetics (VA) are non-flammable inhalation anesthetic agents approved for both induction and maintenance of general anesthesia. There are several volatile anesthetics, we here review halothane (2-bromo-2-chloro-1,1,1-trifluoroethane), isoflurane (1-chloro-2,2,2-trifluoroethyl difluoromethyl ether) and sevoflurane (1,1,1,3,3,3-hexafluoro-2-fluoromethoxy propane).  The precise mechanism of action of VA remains unknown. The loss of perception of sensations and unconsciousness effect is believed to be the consequence of changes in neuronal lipid membrane matrix thickness or other lipophilic sites in different CNS areas. Based in part on correlations between lipophilicity and anesthetic potency, studies suggest potential interference with the hydrophobic portion of neuronal ion channel membrane proteins which in turn can affect the gating properties of these channels. It is speculated that the disruption of these channels is responsible for the inotropic action of VA by reducing Ca^2+^ availability, decreasing responsiveness of the contractile proteins to Ca^2+^, and inhibiting mitochondrial function.  VA are hepatically metabolized by the cytochrome P450 system, both oxidatively and reductively. A portion is also excreted through lung exhalation and renal filtration (Eckenhoff and Shuman 1990; Humphrey et al 2002; Sonner and Cantor 2013).  The toxic effect of VA stem mainly from the increased cell permeability and cell destruction (Berman and Tattersall 1982). There is a parsympathomimetic effect on the cardiovascular system which may sensitize the myocardium to the effect of catecholamines (Tolas et al 1967).  VA can cause dose-dependent hemodynamic effects with decrease in mean arterial pressure, cardiac output, left ventricular function, and systemic vascular resistance.  Isoflurane can lead to a skeletal muscle hypermetabolic state requiring high oxygen demand triggered and symptoms of muscle rigidity, tachycardia, tachypnea, cyanosis, arrhythmias, and unstable blood pressure. This hypermetabolic state may also manifest with body temperature elevation, increase CO2 absorption, decrease PaO2 and pH and hyperkalemia. Isoflurane can also cause profound respiratory depression.  Halothane, which requires higher doses can cause hepatitis which may be more of a hypersensitivity reaction than a direct toxic action (Dienstag 1980), but, since halothane produces several potentially hepatotoxic metabolites, the exact mechanism is still uncertain. Halothane is no longer commercially available in the US. |
| **Theoretical effects on mitochondrial function** |
| Volatile agents only depress mitochondria at doses higher than their clinical used concentrations. VA have been shown to inhibits oxidative phosphorylation and decrease ATP production in vitro. |
| **Known effects on mitochondrial function** |
| Effect on normal mitochondria:  - Inhibit NADH oxidation (complex I) in isolated liver/heart mitochondria. - Inhibit NADH:DBQ oxidoreductase activity in a dose-dependent fashion  - Produce reversible, dose-dependent changes in cellular autofluorescence, consistent with an increase in concentration of the reduced forms of nicotinamide adenine nucleotides and flavoproteins. - Inhibit *gas-1* (general anesthetic sensitive) gene (encodes 49-KDa subunit of complex 1 of the respiratory chain) decreasing the function of complex 1. - Inhibits succinate oxidation and succinate dehydrogenase activity (complex II)  - Induce flavoprotein oxidation through opening of the mitoK(ATP) channel.  - Clinical concentrations mildly depolarize pre-synaptic mitochondria inhibiting respiration by reversed function of the ATP synthase (complex V, partly caused by activation of mitoKATP) - Inhibitory effects upon norepinephrine-induced glucose uptake in neonatal cardiomyocytes decrease glucose uptake, lowers intracellular calcium and diminishes myocardial contractility (Pagel et al 1993; Lee et al 2015).  Effects on dysfunctional mitochondria:  - Studies in model organisms have shown that when complex I is abnormal, sensitivity to volatile anesthetics is markedly increased.  -Higher doses are needed for anesthesia compared to other (newer generation) volatile anesthetic.  - In C. elegans, isoflurane inhibited transfer of electrons from complex I to coenzyme Q, while complexes II, III and IV were resistant to inhibition. Complex V is only minimally affected.  - Blocking complex I and V in the respiratory chain before administration of sevoflurane or isoflurane caused changes in the mitochondrial membrane potential.  - There was decrease in ATP synthesis by mitochondria with VA use (Bains et al 2006). |
| **Effects measured/seen in mitochondrial patients, mitochondrial cell lines or normal cell lines** |
| Cell lines  VA promote a reduced redox state and oxidative phosphorylation by direct attenuation of NADH dehydrogenase (complex I) and cytochrome bc1 (complex III). Low levels of ATP entering the matrix through VDAC/ANT results in reduced ATP hydrolysis and maintain cell ATP levels during reperfusion. This is suspected to contribute to the cardioprotection of VA by diminishing the need for glycolysis, shifts cellular calcium exchange and decreasing lactic acidosis (Agarwal et al 2014)  Animal models 1) Several studies have assessed the effects of VA in animal models (c. elegans, nematodes, drosophilia, mice, rats) specifically looking into oxidative stress and inflammation in cardiac and lung disease models. VA have been shown to procure a protective effect on rat ventricular myocyte during hypoxic, oxidative stress and neutrophil-induced inflammatory response including in models of ischemia-reperfusion. Both sevoflurane and isoflurane were found to decrease oxidative markers, increase antioxidant enzymes and regulate apoptosis-related genes.  These effects were reproduced in models of coronary artery disease, diabetes, stroke and sepsis where pretreatment with VA was reported to decrease IL-6, monocyte chemoattractant protin-1 (MCP-1) and increase survival rates up to 83% (Lee et al 2015).  2) Repeated use of VA resulted in greater synaptic loss relative to a single 2-h exposure. Both single and repeated VA exposures resulted in equivalent reductions in the fraction of presynaptic terminals containing mitochondria. This suggests a “threshold effect” for VA anesthesia with sensitization to synaptic neurotoxicity with subsequent anesthetic use (Amrock et al 2015)  3) Sedation with midazolam followed by anesthesia with nitrous oxide and isoflurane in mice pups caused 30% upregulation of reactive oxygen species, downregulation of superoxide dismutase and impaired mitochondrial dynamic with excessive mitochondrial fission (Boscolo et al 2013).  4) Mice pups exposed to short term VA during the critical synaptogenic period induces mitochondrial hyperactivity and transient imbalance of excitatory/inhibitory synaptic transmission. This however did not lead to long-lasting behavioral changes in mice (Chung et al 2017).  Humans  1) In 13 patients undergoing elective surgeries (abdominal, ENT, orthopedic.), the use of volatile anesthetic was associated with mixed effects including presence or absence of DNA damage, inflammatory response or oxidative stress in general (Lee et al 2015).  2) One complicated case in a 13-month-old with suspected energy metabolism disorder (no molecular diagnosis, elevated lactate and pyruvate and decreased activity of complex I, III and IV) was reported. She underwent Nissen fundoplication for persistent vomiting, complicated by cardiomegaly, pneumonia and cholecystitis for which she received urgent surgery. Anesthesia was induced with thiopental and fentanyl and maintained with isoflurane in nitrous oxide and oxygen. Although surgery was well tolerated, patient developed post-operative hypertonia, bulging fontanel and developed agitation, arching and posturing. Brain MRI 9 days after surgery revealed marked increase in T2 signal intensity in the white matter, including the globus pallidi and cerebellum which appeared to be new (Casta et al 1997).  3) 16 patients with various biochemical ETC dysfunctions underwent procedures using sevoflurane (skin Bx, muscle bx). Patients with complex I deficiency seem to have a higher sensitivity to the volatile anesthetic dose and required a much lower concentration to archive anesthesia (Morgan et al 2002; Allen et al 2017)  4) A review of published mitochondrial cases undergoing anesthesia with several compounds showed that patients who used volatile anesthetics had no side effects reported regardless of whether they had a biochemical ETC deficiency or a specific molecular diagnosis. There was also no difference in patients who have used the volatile anesthetic for induction or maintenance of the anesthesia (Footitt et al 2008).  5) Arrythmias in KSS cases where halothane was used have been reported (Lauwers et al 1994) but safe VA use was also reported (Calzavacca et al 2011)  6) Children with complex I deficiency seem to be more sensitive to VA and require lower dosing (Morgan et al 2002).  7) Many patients with mitochondrial disease have used sevoflurane anesthesia without any complication (Footitt et al 2008; Smith et al 2017). |
| **References** |
| Agarwal B, Stowe DF, Dash RK et al (2014) Mitochondrial targets for volatile anesthetics against cardiac ischemia-reperfusion injury. *Front Physiol* 5: 341.  Allen C, Perkins R, Schwahn B (2017) A retrospective review of anesthesia and perioperative care in children with medium-chain acyl-CoA dehydrogenase deficiency. *Paediatr Anaesth* 27: 60-5.  Amrock LG, Starner ML, Murphy KL et al (2015) Long-term effects of single or multiple neonatal sevoflurane exposures on rat hippocampal ultrastructure. *Anesthesiology* 122: 87-95.  Ayoub S, Berson G, Rock E et al (1991) Mitochondrial nucleotide translocase from skeletal muscle of halothane sensitive pigs: an electrophoretic study. *Cell Biochem Funct* 9: 193-9.  Bains R, Moe MC, Larsen GA et al (2006) Volatile anaesthetics depolarize neural mitochondria by inhibiton of the electron transport chain. *Acta Anaesthesiol Scand* 50: 572-9.  Bains R, Moe MC, Vinje ML (2009) Sevoflurane and propofol depolarize mitochondria in rat and human cerebrocortical synaptosomes by different mechanisms. *Acta Anaesthesiol Scand* 53: 1354-60.  Berman MC, Kewley CF, Kench JE (1974) Contribution of inhibition of NADH-dehydrogenase to the cardiotoxic effects of halothane. *J Mol Cell Cardiol* 6: 39-47.  Berman P, Tattersall M (1982) Self-poisoning with intravenous halothane. *Lancet* 1: 340.  Boscolo A, Milanovic D, Starr JA et al (2013) Early exposure to general anesthesia disturbs mitochondrial fission and fusion in the developing rat brain. *Anesthesiology* 118: 1086-97.  Branca D, Roberti MS, Lorenzin P et al (1991) Influence of the anesthetic 2,6-diisopropylphenol on the oxidative phosphorylation of isolated rat liver mitochondria. *Biochem Pharmacol* 42: 87-90.  Branca D, Toninello A, Scutari G et al (1986) Involvement of long-chain acyl CoA in the antagonistic effects of halothane and L-carnitine on mitochondrial energy-linked processes. *Biochem Biophys Res Commun* 139: 303-7.  Branca D, Varotto ML, Vincenti E (1988) The inhibition of calcium efflux from rat liver mitochondria by halogenated anesthetics. *Biochem Biophys Res Commun* 155: 978-83.  Calzavacca P, Schmidt W, Guzzi M (2011) General anaesthesia for laparoscopic cholecystectomy in a patient with the kearns-sayre syndrome. *Case Rep Anesthesiol* 2011: 806086.  Casta A, Quackenbush EJ, Houck CS et al (1997) Perioperative white matter degeneration and death in a patient with a defect in mitochondrial oxidative phosphorylation. *Anesthesiology* 87: 420-5.  Chow SY, Woon KL (2015) General anesthesia for adults with mitochondrial myopathy. *A A Case Rep* 4: 52-7.  Chung W, Ryu MJ, Heo JY et al (2017) Sevoflurane Exposure during the Critical Period Affects Synaptic Transmission and Mitochondrial Respiration but Not Long-term Behavior in Mice. *Anesthesiology* 126: 288-99.  Dienstag JL (1980) Halothans hepatitis: allergy or idiosyncrasy? *N Engl J Med* 303: 102-4.  Driessen J, Willems S, Dercksen S (2007) Anesthesia-related morbidity and mortality after surgery for muscle biopsy in children with mitochondrial defects. *Paediatr Anaesth* 17: 16-21.  Eckenhoff RG, Shuman H (1990) Subcellular distribution of an inhalational anesthetic in situ. *Proc Natl Acad Sci U S A* 87: 454-7.  Falk MJ, Kayser EB, Morgan PG (2006) Mitochondrial complex I function modulates volatile anesthetic sensitivity in C. elegans. *Curr Biol* 16: 1641-5.  Falk MJ, Rosenjack JR, Polyak E, et al (2009) Subcomplex Ilambda specifically controls integrated mitochondrial functions in Caenorhabditis elegans. *PLoS One* 4: e6607.  Farag E, Argalious M, Narouze S et al (2002) The anesthetic management of ventricular septal defect (VSD) repair in a child with mitochondrial cytopathy. *Can J Anaesth* 49: 958-62.  Ferri A, Nencini M, Cozzolino M et al (2008) Inflammatory cytokines increase mitochondrial damage in motoneuronal cells expressing mutant SOD1. *Neurobiol Dis* 32: 454-60.  Fletcher JE, Mayerberger S, Tripolitis L et al (1991) Fatty acids markedly lower the threshold for halothane-induced calcium release from the terminal cisternae in human and porcine normal and malignant hyperthermia susceptible skeletal muscle. *Life Sci* 49: 1651-7.  Footitt EJ, Sinha MD, Raiman JA et al (2008) Mitochondrial disorders and general anaesthesia: a case series and review. *Br J Anaesth* 100: 436-41.  Grist EM, Baum H (1974) A possible mechanism for the halothane-induced inhibition of mitochondrial respiration: binding of endogenous calcium to NADH dehydrogenase. *FEBS Lett* 48: 41-4.  Groeben H, Meier S, Tankersley CG et al (2004) Influence of volatile anaesthetics on hypercapnoeic ventilatory responses in mice with blunted respiratory drive. *Br J Anaesth* 92: 697-703.  Hall GM, Kirtland SJ, Baum H (1973) The inhibition of mitochondrial respiration by inhalational anaesthetic agents. *Br J Anaesth* 45: 1005-9.  Hanley PJ, Loiselle DS (1998) Mechanisms of force inhibition by halothane and isoflurane in intact rat cardiac muscle. *J Physiol* 506 ( Pt 1): 231-44.  Hanley PJ, Ray J, Brandt U et al (2002) Halothane, isoflurane and sevoflurane inhibit NADH:ubiquinone oxidoreductase (complex I) of cardiac mitochondria. *J Physiol* 544: 687-93.  Hartman PS, Ishii N, Kayser EB et al (2001) Mitochondrial mutations differentially affect aging, mutability and anesthetic sensitivity in Caenorhabditis elegans. *Mech Ageing Dev* 122: 1187-201.  Humeidan ML, Dalia J, Traetow WD (2016) Anesthetic considerations for renal transplant surgery in patients with mitochondrial myopathy, encephalopathy, lactic acidosis, and stroke-like episodes syndrome: a case report. *J Clin Anesth* 34: 344-7.  Humphrey JA, Sedensky MM, Morgan PG (2002) Understanding anesthesia: making genetic sense of the absence of senses. *Hum Mol Genet* 11: 1241-9.  Jiang MT, Nakae Y, Ljubkovic M et al (2007) Isoflurane activates human cardiac mitochondrial adenosine triphosphate-sensitive K+ channels reconstituted in lipid bilayers. *Anesth Analg* 105: 926-32.  Kayser EB, Morgan PG, Sedensky MM (1999) GAS-1: a mitochondrial protein controls sensitivity to volatile anesthetics in the nematode Caenorhabditis elegans. *Anesthesiology* 90: 545-54.  Kishikawa JI, Inoue Y, Fujikawa M et al (2018) General anesthetics cause mitochondrial dysfunction and reduction of intracellular ATP levels. *PLoS One* 13: e0190213.  Kohro S, Hogan QH, Nakae Y et al (2001) Anesthetic effects on mitochondrial ATP-sensitive K channel. *Anesthesiology* 95: 1435-40.  Kohro S, Hogan QH, Nakae Y et al (2003) Repeated or prolonged isoflurane exposure reduces mitochondrial oxidizing effects. *Anesthesiology* 98: 275-8.  Kudo M, Aono M, Lee Y et al (2001) Effects of volatile anesthetics on N-methyl-D-aspartate excitotoxicity in primary rat neuronal-glial cultures. *Anesthesiology* 95: 756-65.  Kudoh A, Matsuki A (2000) Halothane and sevoflurane decrease norepinephrine-stimulated glucose transport in neonatal cardiomyocyte. *Anesth Analg* 91: 1151-9.  Lauwers MH, Van Lersberghe C, Camu F (1994) Inhalation anaesthesia and the Kearns-Sayre syndrome. *Anaesthesia* 49: 876-8.  Lee MJ, Harris RA, Wakabayashi T et al (1971) The inhibition of mitochondrial energized processes by fluorescein mercuric acetate. *J Bioenerg* 2: 13-31.  Lee YM, Song BC, Yeum KJ (2015) Impact of Volatile Anesthetics on Oxidative Stress and Inflammation. *Biomed Res Int* 2015: 242709.  Ljubkovic M, Mio Y, Marinovic J et al (2007) Isoflurane preconditioning uncouples mitochondria and protects against hypoxia-reoxygenation. *Am J Physiol Cell Physiol* 292: C1583-90.  Merin RG, Kumazawa T, Honig CR (1975) Halothane decreases actomyosin ATPase activity: a possible mechanism of the negative inotropic effect. *Recent Adv Stud Cardiac Struct Metab* 5: 405-12.  Miller RN, Hunter FE, Jr. (1971) Is halothane a true uncoupler of oxidative phosphorylation? *Anesthesiology* 35: 256-61.  Miro O, Barrientos A, Alonso JR et al (1999) Effects of general anaesthetic procedures on mitochondrial function of human skeletal muscle. *Eur J Clin Pharmacol* 55: 35-41.  Mitchelson KR, Hird FJ (1973) Effect of pH and halothane on muscle and liver mitochondria. *Am J Physiol* 225: 1393-8.  Moe MC, Bains R, Vinje ML (2004) Sevoflurane depolarizes pre-synaptic mitochondria in the central nervous system. *Acta Anaesthesiol Scand* 48: 562-8.  Morgan PG, Hoppel CL, Sedensky MM (2002) Mitochondrial defects and anesthetic sensitivity. *Anesthesiology* 96: 1268-70.  Muravyeva M, Sedlic F, Dolan N et al (2013) Preconditioning by isoflurane elicits mitochondrial protective mechanisms independent of sarcolemmal KATP channel in mouse cardiomyocytes. *J Cardiovasc Pharmacol* 61: 369-77.  Nahrwold ML, Cohen PJ (1973) The effects of forane and fluroxene on mitochondrial respiration: correlation with lipid solubility and in-vivo potency. *Anesthesiology* 38: 437-44.  Nahrwold ML, Lecky JH, Cohen PJ (1974) The effect of halothane on mitochondrial permeability to NADH. *Life Sci* 15: 1261-5.  Nakae Y, Kwok WM, Bosnjak ZJ et al (2003) Isoflurane activates rat mitochondrial ATP-sensitive K+ channels reconstituted in lipid bilayers. *Am J Physiol Heart Circ Physiol* 284: H1865-71.  Nelson TE (1991) Effect of halothane on human skeletal muscle sarcoplasmic reticulum calcium-release channel. *Adv Exp Med Biol* 301: 21-30.  Ohlson KB, Shabalina IG, Lennstrom K et al (2004) Inhibitory effects of halothane on the thermogenic pathway in brown adipocytes: localization to adenylyl cyclase and mitochondrial fatty acid oxidation. *Biochem Pharmacol* 68: 463-77.  Pagel PS, Kampine JP, Schmeling WT et al (1993) Reversal of volatile anesthetic-induced depression of myocardial contractility by extracellular calcium also enhances left ventricular diastolic function. *Anesthesiology* 78: 141-54.  Pravdic D, Hirata N, Barber L et al (2012) Complex I and ATP synthase mediate membrane depolarization and matrix acidification by isoflurane in mitochondria. *Eur J Pharmacol* 690: 149-57.  Quintana A, Morgan PG, Kruse SE et al (2012) Altered anesthetic sensitivity of mice lacking Ndufs4, a subunit of mitochondrial complex I. *PLoS One* 7: e42904.  Rafique MB, Cameron SD, Khan Q et al (2013) Anesthesia for children with mitochondrial disorders: a national survey and review. *J Anesth* 27: 186-91.  Ramadasan-Nair R, Hui J, Zimin PI et al (2017) Regional knockdown of NDUFS4 implicates a thalamocortical circuit mediating anesthetic sensitivity. *PLoS One* 12: e0188087.  Redshaw C, Stewart C (2014) Anesthetic agents in patients with very long-chain acyl-coenzyme A dehydrogenase deficiency: a literature review. *Paediatr Anaesth* 24: 1115-9.  Roelofs S, Manjeri GR, Willems PH et al (2014) Isoflurane anesthetic hypersensitivity and progressive respiratory depression in a mouse model with isolated mitochondrial complex I deficiency. *J Anesth* 28: 807-14.  Rottenberg H (1983) Uncoupling of oxidative phosphorylation in rat liver mitochondria by general anesthetics. *Proc Natl Acad Sci U S A* 80: 3313-17.  Rusy BF, Komai H (1987) Anesthetic depression of myocardial contractility: a review of possible mechanisms. *Anesthesiology* 67: 745-66.  Sanchez V, Feinstein SD, Lunardi N et al (2011) General Anesthesia Causes Long-term Impairment of Mitochondrial Morphogenesis and Synaptic Transmission in Developing Rat Brain. *Anesthesiology* 115: 992-1002.  Shapiro F, Athiraman U, Clendenin DJ et al (2016) Anesthetic management of 877 pediatric patients undergoing muscle biopsy for neuromuscular disorders: a 20-year review. *Paediatr Anaesth* 26: 710-21.  Smith A, Dunne E, Mannion M et al (2017) A review of anaesthetic outcomes in patients with genetically confirmed mitochondrial disorders. *Eur J Pediatr* 176: 83-8.  Sonner JM, Cantor RS (2013) Molecular mechanisms of drug action: an emerging view. *Annu Rev Biophys* 42: 143-67.  Sosunov SA, Ameer X, Niatsetskaya ZV et al (2015) Isoflurane anesthesia initiated at the onset of reperfusion attenuates oxidative and hypoxic-ischemic brain injury. *PLoS One* 10: e0120456.  Stadnicka A, Marinovic J, Ljubkovic et al (2007) Volatile anesthetic-induced cardiac preconditioning. *J Anesth* 21: 212-9.  Sui B, Zhang GM, Yu WF et al (1999) Experimental research on phospholipids variation of halothane on liver mitochondria. *World J Gastroenterol* 5: 28-30.  Tolas AG, Allen GD, Ward RJ et al (1967) Comparison of effects of methods of induction of anesthesia on cardiac rhythm. *J Oral Surg* 25: 54-9.  Wallace JJ, Perndt H, Skinner M (1998) Anaesthesia and mitochondrial disease. *Paediatr Anaesth* 8: 249-54.  Wu W, Zhou X, Liu P et al (2014) Isoflurane reduces hypoxia/reoxygenation-induced apoptosis and mitochondrial permeability transition in rat primary cultured cardiocytes. *BMC Anesthesiol* 14: 17.  Yamamoto T, Miyazawa N, Yamamoto S et al (2017) Anesthetic Management in Mitochondrial Encephalomyopathy: A Case Report. *Anesth Prog* 64: 235-9.  Yang H, Liang G, Hawkins BJ et al (2008) Inhalational anesthetics induce cell damage by disruption of intracellular calcium homeostasis with different potencies. *Anesthesiology* 109: 243-50.  Zhang Y, Dong Y, Wu X et al (2010) The mitochondrial pathway of anesthetic isoflurane-induced apoptosis. *J Biol Chem* 285: 4025-37.  Zhao P, Ji G, Xue H et al (2014) Isoflurane postconditioning improved long-term neurological outcome possibly via inhibiting the mitochondrial permeability transition pore in neonatal rats after brain hypoxia-ischemia. *Neuroscience* 280: 193-203.  Zimin PI, Woods CB, Kayser EB et al (2018) Isoflurane disrupts excitatory neurotransmitter dynamics via inhibition of mitochondrial complex I. *Br J Anaesth* 120: 1019-32. |
